# Social Prescribing: Systematic Review of the Effectiveness of Psychosocial Community Referral Interventions in Primary Care

**DOI:** 10.5334/ijic.6472

**Published:** 2022-08-19

**Authors:** Hendrik Napierala, Karen Krüger, Doreen Kuschick, Christoph Heintze, Wolfram J. Herrmann, Felix Holzinger

**Affiliations:** 1 Charité – Universitätsmedizin Berlin, corporate member of Freie Universität Berlin and Humboldt Universität zu Berlin, Institute of General Practice and Family Medicine, Charitéplatz 1, 10117 Berlin, Germany

**Keywords:** social prescribing, community referral intervention, systematic review, integrated care

## Abstract

**Introduction:**

Social prescribing (SP) aims to provide targeted psychosocial support and close the gap between medical and non-medical services. This review assesses the effectiveness of community-based SP interventions.

**Methods:**

We performed a systematic review and qualitative synthesis of interventional studies of community referral interventions focused on facilitating psychosocial support. We considered health-related endpoints, other patient reported outcomes, or health care utilization. Six databases, grey literature, and additional trials registers were searched. Results were screened in a two-step process, followed by data extraction, each by two independent reviewers. If data permitted such, effect sizes were calculated. Risk of bias was assessed with the EPHPP and the Cochrane RoB2 tools.

**Results:**

We identified 68 reports from 53 different projects, three were controlled studies. Uncontrolled studies with shorter time frames frequently reported positive effects. This could largely not be seen in controlled settings and for longer follow-up periods. Designs, populations, and outcomes evaluated were heterogeneous with high risk of bias for most studies.

**Discussion and conclusion:**

Current evidence suggests positive effects of SP on a variety of relevant endpoints. Due to quality deficits in the available studies, scope for conclusions concerning clinical relevance and sustainability is limited. Further methodologically rigorous controlled trials are needed.

## Abstract (de)

**Einleitung:**

Social prescribing (SP) soll gezielte psychosoziale Unterstützung ermöglichen und die Lücke zwischen medizinischen und nicht-medizinischen Angeboten schließen. Diese Übersichtsarbeit untersucht die Wirksamkeit von gemeindebasierten SP-Interventionen.

**Methoden:**

Es erfolgte eine systematische Übersichtsarbeit und qualitative Synthese von Interventionsstudien zu gemeindebasierten Überweisungs-Interventionen, die auf eine Förderung psychosozialer Unterstützung abzielen. Es wurden gesundheitsrelevante Endpunkte, andere patientenberichtete Outcomes, sowie die Inanspruchnahme von Gesundheitsleistungen berücksichtigt. Die Suche erfolgte in sechs Datenbanken, grauer Literatur, sowie zusätzlich in Studienregistern. Die Ergebnisse wurden in einem zweistufigen Prozess gesichtet, gefolgt von der Datenextraktion. Beide Schritte erfolgten durch zwei unabhängige Reviewer. Bei ausreichend verfügbaren Daten wurden Effektstärken errechnet. Das Verzerrungspotenzial wurde mit den EPHPP und Cochrane RoB2 Instrumenten bewertet.

**Ergebnisse:**

Es wurden 68 Berichte aus 53 unterschiedlichen Projekten identifiziert, darunter waren drei kontrollierte Studien. Nicht-kontrollierte Studien mit kürzeren Betrachtungszeiträumen berichteten häufig positive Effekte. In kontrollierten Settings und über längere Beobachtungszeiten konnte dies überwiegend nicht gezeigt werden. Betrachtete Designs, Populationen und Endpunkte waren heterogen, das Verzerrungspotential in den meisten Studien hoch.

**Diskussion und Schlussfolgerungen:**

Die derzeitige Evidenz legt positive Effekte von SP auf eine Reihe relevanter Endpunkte nahe. Aufgrund von Qualitätsdefiziten in den verfügbaren Studien können die klinische Relevanz und die Langfristigkeit der Effekte nur eingeschränkt beurteilt werden. Zusätzliche methodisch hochwertige Studien sind erforderlich.

## Introduction

Psychosocial problems play a major role in primary care [1] and have a considerable impact on manifestation and course of mental and somatic conditions [2,3,4]. Psychosocial issues typically seen in primary care include loneliness and restrictions in social participation of elderly multi-morbid patients, problems in the family and at work, as well as financial difficulties [1]. Psychosocial problems are also associated with considerable economic costs, e.g. caused by sick leave and long-term absence from work [5, 6].

While General Practitioners (GPs) can target only few problems during their consultations [7], there is an extensive range of social care and counseling in many countries. Consequently, there may frequently be no formalized connections between psychosocial counseling services and primary care [8]. Depending on local circumstances, the diversity of offers in the non-medical sector may confuse providers. For an individual, choosing the right contact point might be challenging.

Social prescribing (SP) is an innovative and promising approach for providing vulnerable groups with psychosocial support and bridge the gap between medical and psychosocial services [9]. It equips GPs with a non-medical referral option which can accompany existing treatments to improve health and well-being. SP is usually implemented by involving a “link worker” to whom patients with psychosocial problems are referred (= issued a “social prescription”). The link worker identifies non-medical problems and initiates subsequent contact with appropriate local services [10]. The SP concept combines two key forms of integrated care: horizontal integration with its focus on multidisciplinary support, as well as people-centered integration aimed at empowerment and self-management [11].

SP has been implemented in pilot trials, especially in the United Kingdom (UK), and published evaluations have demonstrated improvement with respect to relevant outcomes, such as quality of life, anxiety and depression [12,13,14,15], reduction in healthcare utilization [16,17,18] and workload reduction for primary care physicians [19], as well as earlier return to work [15]. Nevertheless, the methodological spectrum and manner of reporting are heterogeneous, ranging from formally publicized randomized controlled trials [20] to non-peer-reviewed before-and-after studies. Previous systematic reviews have either focused on specific outcomes [21] or were limited to studies in the UK [22,23], thus leaving important questions unanswered.

Therefore, this systematic review aims to assess the effectiveness of SP for facilitating psychosocial support with an international focus.

## Methods

### Protocol

The protocol was published a priori in the PROSPERO registry (registration number: CRD42020182562 [24]). The protocol development was supervised by an independent advisory board including experts from primary care and public health as well as patient representatives.

### Eligibility criteria

The target population consisted of adults with an actual or assumed need for psychosocial support or counseling. Interventions were eligible if they consisted of a community referral intervention aimed at psychosocial support or counseling and if the referral was initiated by an outpatient medical provider (primary care providers, other physicians, nurses, outpatient clinics etc.). In this context, referrals to a facilitator, coordinator or institution assessing needs and initiating appropriate measures were considered pertinent. We included controlled trials as well as uncontrolled studies with a before-and-after design. We considered all health-related endpoints and surrogate parameters, any other patient reported outcomes, as well as any measures of health care utilization. Measurements needed to be assessed at least at two different time points and/or in two different groups with no limit to the length of follow-up.

### Information sources and search strategy

We searched the following electronic bibliographic databases: MEDLINE, Embase, SocIndex, Social Care Online, CINAHL, and the Cochrane Library (Cochrane Database of Systematic Reviews, Cochrane Central Register of Controlled Trials). We conducted a search on ClinicalTrials.gov and the International Clinical Trials Registry Platform (ICTRP). We did not apply constraints concerning language or country of origin. The time frame was limited to studies published since 2000, as SP as a concept was established in the late 90s and early 2000s [25,26] with evaluation methods and general rigor of study conduct in health services research concurrently advancing [27]. Supplementary search strategies included analysis of bibliographies of studies and reviews identified by the electronic search (citation tracking), as well as an internet search with defined keywords for identification of “grey literature”. Such resources beyond formal scientific publishing were also retrieved from pertinent information repositories: Open Grey, Grey Literature Report and Grey Guide Repository. All search algorithms used can be found in the supplement (**Appendix 1**). The initial search was conducted on May 5^th^ 2020, an update search was performed on February 1^st^ 2021.

### Study selection and data extraction

After removal of duplicates, we performed a two-step screening process to select studies meeting inclusion criteria. Firstly, two reviewers independently assessed all titles and abstracts. Secondly, two reviewers independently assessed all eligible full texts. In both steps, disagreement was resolved through discussion, and where necessary by consulting a third researcher. A standardized data extraction sheet was developed and piloted. Two reviewers extracted data independently. Discrepancies were resolved by discussion. *Zotero* [28] was used for collecting and de-duplicating publications and *Covidence* [29] for selecting studies and extracting data.

### Risk of bias

We used the Effective Public Health Practice Project’s Quality Assessment Tool for Quantitative Studies (EPHPP) for study-level quality appraisal [30,31]. Additionally, we assessed the quality of randomized controlled trials with the Cochrane Risk of Bias tool for randomized trials (RoB 2) [32]. Two reviewers assessed risk of bias independently. Risk of bias was visualized using the *robvis* package [33].

### Synthesis of results

Findings were summarized descriptively. For purposes of judicious data analysis and reporting, outcomes had to be classified in thematic groups and prioritized based on the scope of available data. Thematic classification was based on the Common Outcomes Framework, focusing on the impact on the person and on the health and care system [34]. The six groups were: mental health, mental wellbeing, quality of life and general health, loneliness, self-efficacy, and health care utilization. Concerning length of follow-up for longitudinal outcome assessment, time frames were classified into short (0–3 months), intermediate (>3–6 months) and long term (>6 months). For continuous outcome parameters with sufficient information and data quality, we calculated standardized mean differences (SMD) as measures of effect size. We used test statistics or p values and degrees of freedom to approximate SMD using the *effectsize* package [35] in *R* [36]. When SMDs could be computed, visualization was performed with the *meta* package [37]. We refrained from calculating and presenting a pooled effect measure as to not exceed the body of evidence’s potential. Subgroup analyses were neither feasible.

## Results

### Study selection

**Figure 1:**
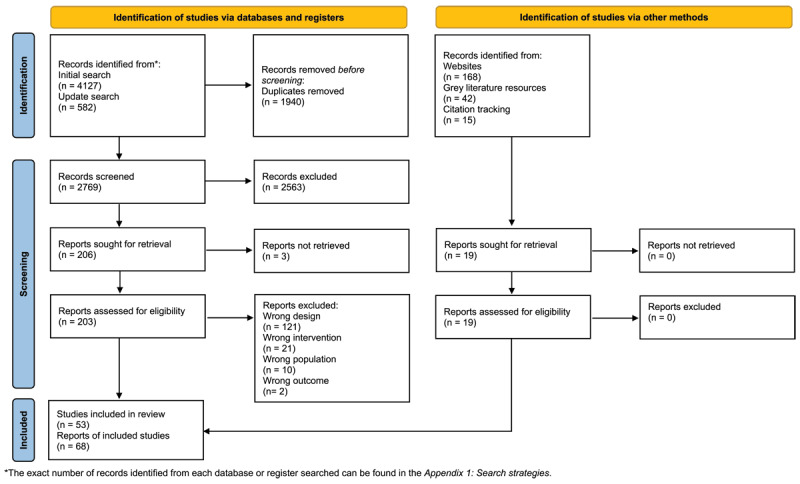
Revised PRISMA Flow diagram. *Note*: The diagram shows the iterative steps of literature screening and selection. Numbers refer to the reports identified. For some interventional studies, more than one report was located. The bottommost box shows total numbers of included reports and underlying SP projects.

We identified 4934 records and included 53 studies with 68 publications in our review. The study selection process is outlined in the revised PRISMA flow diagram (Figure 1) [38]. **Appendix 2** provides a list of publications excluded in the full text screening stage, as well as reasons for exclusion.

### Study characteristics

**Figure 2:**
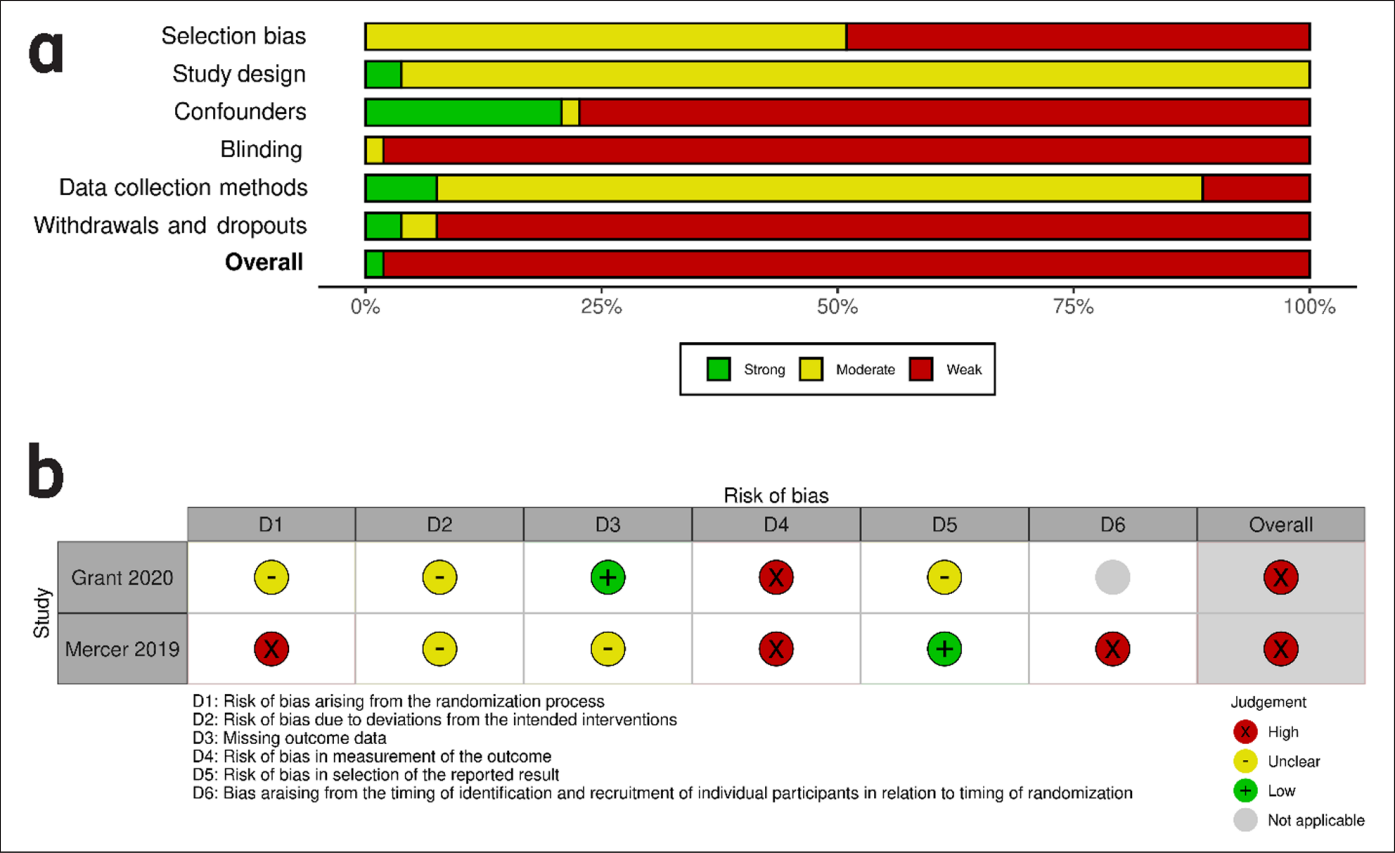
**a:** Risk of bias within studies: EPHPP for non-randomized studies, summary plot. **b:** Risk of bias within studies: RoB2 for randomized studies, traffic light plot. *Note*: Figure 2a: Stacked bars in the summary plot represent percentages of studies with a corresponding rating for individual EPHPP domains and the overall quality judgment. Figure 2b: RoB 2 assessment for randomized studies only. Rows show individual domain and overall ratings for each study, visualized as traffic light symbols.

**Figure 3:**
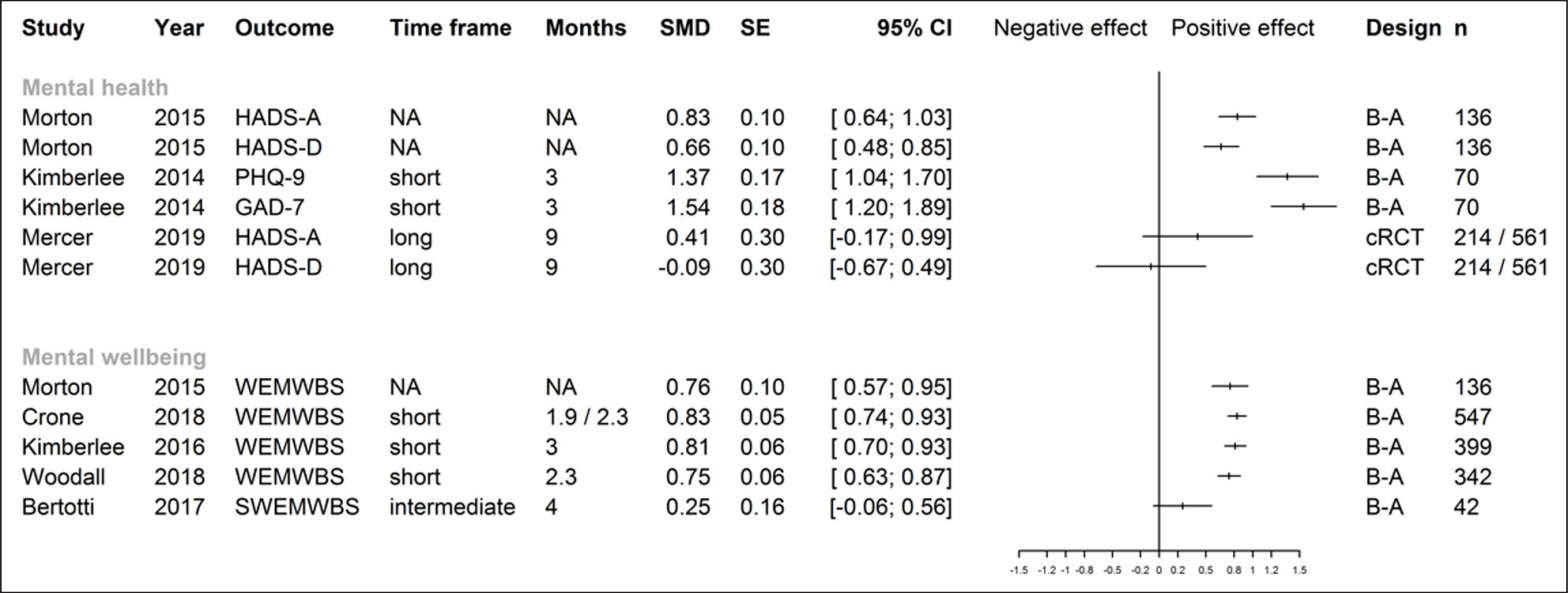
Effect sizes for mental health and mental wellbeing. *Note*: HADS-A Hospital Anxiety and Depression Scale – Anxiety; HADS-D Hospital Anxiety and Depression Scale – Depression; PHQ-9 Patient Health Questionnaire-9; GAD-7 Generalized Anxiety Disorder Scale-7; WEMWBS Warwick Edinburgh Mental Wellbeing Scale; SWEMWBS Short Warwick Edinburgh Mental Wellbeing Scale; Time frame: short (0–3 months), intermediate (>3–6 months) and long term (>6 months); Follow-up time in months: in case of original study reporting follow-up in weeks, these were converted to 30-day months; Kimberlee 2016 [76]: follow-up time of 3 months was intended, but length unclear in half of cases; Woodall 2018 [90]: mean follow-up time; If different numbers of months are shown, follow-up length was different for sub-populations; NA not addressed in original report; B–A before-after study; cRCT cluster randomized controlled trial; n number of patients analyzed for outcome, numbers refer to intervention/control in case of controlled studies; SMD and SE rounded by software package to two digits, confidence intervals were calculated by R package “meta” [37] from SMD/SE values calculated by R package “effect size” [35]. For Mercer 2019 [92,93], effect sizes were taken from the original publication (adjusted SMD).

**Figure 4:**
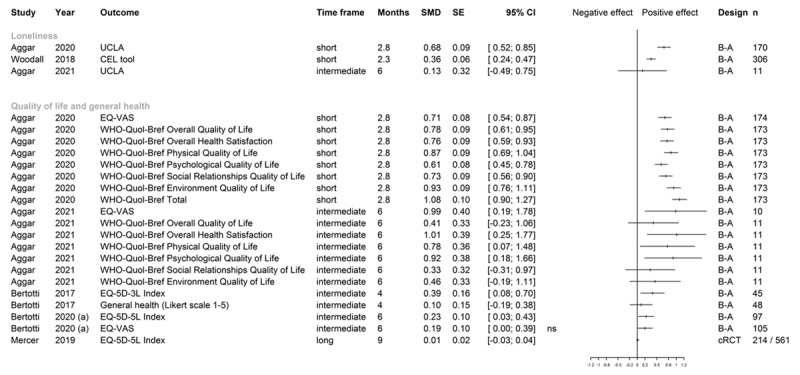
Effect sizes for loneliness, quality of life, and general health. *Note*: UCLA University of California Los Angeles Loneliness Scale; CEL Campaign to End Loneliness ; EQ-VAS European Quality of Life Visual Analogue Scale; WHO-Quol-Bref, WHO Quality of Life short form; EQ-5D-3L/5L European Quality of Life 5 Dimensions 3 Level/5 Level; Time frame: short (0–3 months), intermediate (>3–6 months) and long term (>6 months); Follow-up time in months: in case of original study reporting follow-up in weeks, these were converted to 30-day months; B-A before-after study; cRCT cluster randomized controlled trial; n number of patients analyzed for outcome, numbers refer to intervention/control in case of controlled studies; SMD and SE rounded by software package to two digits, confidence intervals were calculated by R package “meta” [37] from SMD/SE values calculated by R package “effect size” [35]. In cases of 0.00 as a confidence interval bound, the graph shows whether the original study reported a significant (s) or non-significant (ns) result, as the plotting function only shows two digits. For Mercer 2019 [92,93], effect sizes were taken from the original publication (adjusted SMD).

All included studies were conducted in high-income countries, with 51 studies originating from the UK and two from Australia [15,39]. There were 50 uncontrolled studies with a before-and-after design [12,13,14,15,16,17,18,19,39,40,41,42,43,44,45,46,47,48,49,50,51,52,53,54,55,56,57,58,59,60,61,62,63,64,65,66,67,68,69,70,71,72,73,74,75,76,77,78,79,80,81,82,83,84,85,86,87,88,89,90,91], one of these included a module with a controlled design for health care utilization outcomes [84]. Three studies included control groups: one randomized controlled trial [92], one quasi-experimental cluster randomized controlled trial [93], and one non-randomized controlled trial [94,95,96]. Fifteen evaluations were peer-reviewed before publishing, five were partly peer-reviewed (not all publications of the project were peer-reviewed) and 33 were not peer-reviewed. Most reports (n = 41, ~80%) had been published in the last five years, with about half of these dating back to the years 2019 and 2020 (n = 22). Fifteen SP evaluations focused on populations with special characteristics besides their existing psychosocial needs (Mental health issues: n = 6 [12,39,52,53,58,70,80,88], long-term conditions: n = 4 [49,50,51,54,57,60,61,97], geriatric age group: n = 2 [19,73], work-related injuries: n = 1 [15], high risk for cardiovascular disease: n = 1 [84] and sensory impairment: n = 1 [87]).The remaining studies had included unselected/mixed populations or did not provide detailed information about the target group. Sample sizes were highly heterogeneous, ranging from n = 12 [87] to n = 10643 [64]. All studies included into this review reported on a combined total number of n = 50237 patients (n = 41627 receiving SP, n = 8610 controls). One study did not report the total of recruited patients [77]. Only few publications described efforts of accounting for potential biases (Figures 2, 3, 4). Overall, the population across the field of included SP studies can be characterized as adult, of a wide age range, with a predominance of female participants (**Appendix 3**). Many studies included populations with high levels of social deprivation, unemployment, frequently living alone, and often afflicted with long-term conditions. Referral reasons reported were manifold, including social isolation, mental health issues, high primary care utilization, struggles with significant life changes, financial or housing problems, and conflicts at work or in the family, including e.g. special challenges of caregivers. The vast majority of studies was conducted in a primary care setting (**Appendix 3**). Some of the SP projects located the link worker directly in the primary care providers’ practices, while this function was situated in the community in most study setups. According to the concept proposed by Richard Kimberlee [98], SP programs can be delineated into different models based on the interventions’ components and how comprehensively they address the entirety of patients’ needs. Such have been labeled according to intensity as light, medium or holistic. Applying this concept, 30 programs in our study field could be described as holistic, 17 as medium and six as light evaluations. However, classification was difficult for some studies as to partially missing information, and a few programs comprising several alternative pathways (**Appendix 3**). Maximum length of observance of participants after baseline ranged between immediately after the end of intervention [12,15,42,61,69,77,78,82,83] and 18 months [16], but many studies did assess outcomes at more than one time point during follow-up. Regarding the small field of controlled studies, the non-randomized controlled trial reported by Carnes et al. [94,95,96] included two control groups. Assessment of differences in patient outcomes (e.g. quality of life) was based on comparison with a matched control group from six GP practices in the area, while another matched control group from the same GP practices was used for comparative evaluation of GP consultation rates. The control group in the randomized controlled trial by Grant et al. received routine GP care [20]. Mercer et al. [92,93] included randomly chosen patients from comparator practices located in the same area (Glasgow), which were additionally selected on basis of their catering to deprived patient populations. Comparator practices were not allocated a link worker. A detailed overview of all included evaluations can be found in the study characteristics table (**Appendix 3**).

### Risk of bias within studies

Only one study met the criteria prerequisite for a “strong” rating with the EPHPP instrument [60]. All other studies were rated as “weak” (Figure 2a, **Appendix 4**). Overall, potential selection bias, lack of blinding, as well as high attrition rates (in combination with missing information on drop-out reasons) were the main causes for concern regarding risk of bias within studies. As to their randomized controlled designs, two project evaluations [20,92,93] could be additionally assessed with the revised RoB 2 tool (Figure 2b).

## Results of individual studies

### Mental health

**Figure 5:**
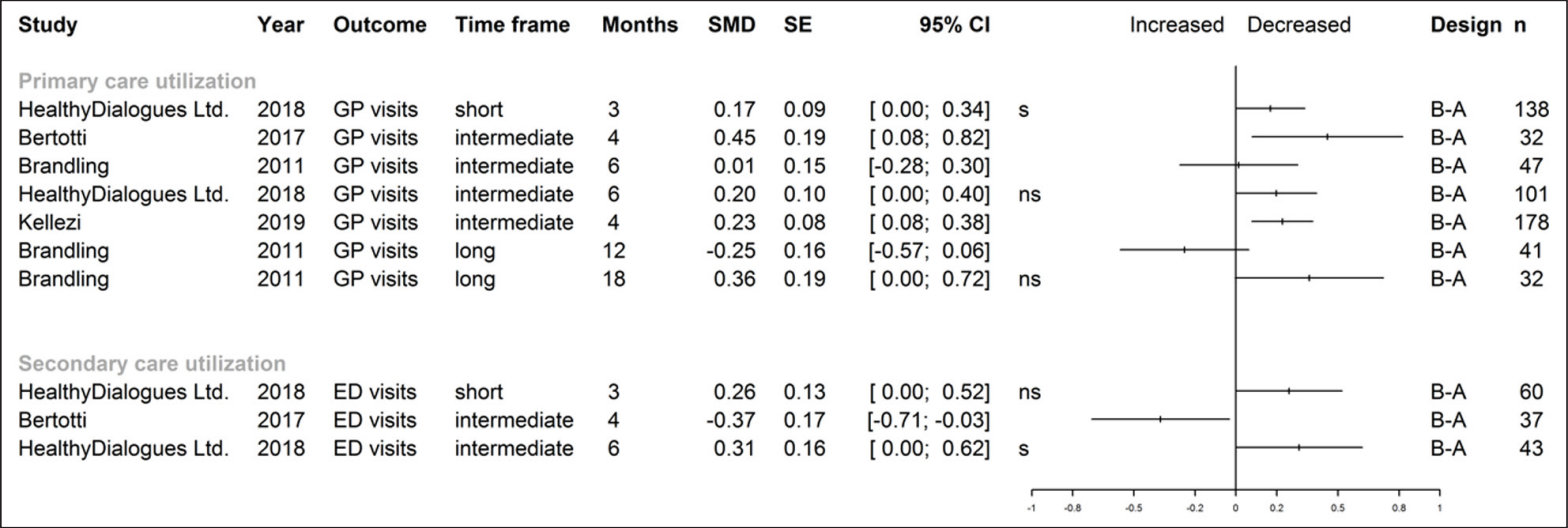
Effect sizes for health care utilization. *Note*: GP general practitioner; ED emergency department; Time frame: short (0–3 months), intermediate (>3–6 months) and long term (>6 months); Follow-up time in months: in case of original study reporting follow-up in weeks, these were converted to 30-day months; B-A before-after study; n number of patients analyzed for outcome; SMD and SE rounded by software package to two digits, confidence intervals were calculated by R package “meta” [37] from SMD/SE values calculated by R package “effect size” [35]. In cases of 0.00 as a confidence interval bound, the graph shows whether the original study reported a significant (s) or non-significant (ns) result, as the plotting function only shows two digits.

The most frequent instrument of assessment was the Hospital Anxiety and Depression scale (HADS), while a few studies used the Generalized Anxiety Disorder Scale-7 (GAD-7) and/or the Patient Health Questionnaire-9 (PHQ-9). SMD for one controlled [92,93] and two uncontrolled studies [12,74,75] could be either computed or extracted (Figure 5). Positive effects were found in uncontrolled studies with shorter time frames, as indicated by the SMDs calculated for the Kimberlee et al. [76] and Morton et al. studies [12], as well as in several uncontrolled studies for which standardized effect sizes could not be computed [13,16,40,41,43,44,56,59,60,61,69,70,80,81,87,91]. This could not be equally shown for controlled settings and longer follow-up periods, as illustrated by the effect sizes for the study by Mercer et al. [92,93]. Grant et al. [20] showed significant improvement in anxiety symptoms (HADS-A) but not in depressive symptoms (HADS-D) versus controls in an intermediate time frame. Carnes et al. [94,95,96] could also not show any long-term group differences for either measure. Regarding further data on mental health outcomes, two uncontrolled studies [78,80] showed unclear improvements on the GAD-7 and PHQ-9 scales in unclear or short follow-up periods.

### Mental wellbeing

For mental wellbeing, the (Short) Warwick Edinburgh Mental Wellbeing Scale (SWEMWBS/WEMWBS) was the most frequently used outcome assessment tool. No controlled studies assessing mental wellbeing could be identified. SMDs could be computed for five uncontrolled studies [12,14,17,76,90] (Figure 3). Four evaluations showed large effects in a short follow-up period, and one evaluation with a small sample size showed no effect for SWEMWBS scores in an intermediate timeframe. This is accompanied by a number of uncontrolled studies with short or intermediate timeframes reporting improvements of mental wellbeing measures with altogether unclear clinical relevance [13,16,40,41,43,44,56,59,60,61,63,64,69,70,80,81,87,91].

### Loneliness

Different scales were used to measure loneliness: University of California at Los Angeles (UCLA) Loneliness Scale, De Jong Gierveld Loneliness Scale, and Campaign to End Loneliness Tool. SMDs could be computed from three uncontrolled studies [15,39,90] (Figure 4). Two evaluations showed medium to large positive effects in a short or unknown follow-up period, one further evaluation showed no differences between pre and post in an intermediate time frame. We found eight further measures from six uncontrolled studies [13,43,70,71,72,79,84] with short to intermediate follow-up lengths, showing improvement with unclear clinical importance from pre to post intervention without information on the effect size.

### Quality of life and general health

While most studies relied on different variations of the EQ-5D, two studies employed the WHO-Quol-Bref to measure quality of life [15,39]. For overall health, a general health Likert scale [17,94,95,96] and COOP/WONCA [20] were used. All three controlled studies and eleven uncontrolled before and after studies measured quality of life and general health at baseline and again at up to eight months after referral. SMDs for one controlled [92,93] and four uncontrolled studies [15,17,39,44,92,93] could be calculated or extracted (Figure 4). Positive effects were observed in a further number of uncontrolled studies [40,43,45,56,59,71,72,90]. This effect could not be shown in two controlled studies [92,93,94,95,96] for an intermediate timeframe. Grant et al. [20] indicated significant improvement in general health. This could not be seen in one uncontrolled [17] and one controlled study [94,95,96] for longer timeframes.

### Self-efficacy

The Patient Activation Measure (PAM) and General Self-Efficacy Scale (GSE) were mainly used to report changes in self-efficacy [12,17,60,84]. We could compute the SMD for the GSE from Morton et al. [12], reporting a large positive effect in an unclear time frame (SMD = 0.85, 95% CI [0.65, 1.05]). Polley et al. and Elston et al. [60,84] reported clinically relevant increases in the PAM score, while Bertotti et al. [17] could not show any difference.

### Health care utilization

Most frequent outcomes reported for health care resources use were longitudinal changes in utilization of primary care providers and emergency departments, reported as mean numbers of consultations for a period pre vs. post intervention/baseline. Results of studies for which effect sizes could be calculated (all were uncontrolled, Figure 5) suggest small to moderate effects in terms of reducing visits in primary or emergency hospital care. If longer time periods were observed, most pre-post studies permitting calculation of effect sizes did not show any significant changes in utilization.

Concerning primary care utilization, controlled studies painted a mixed picture. Carnes et al. [94,95,96] reported significant longitudinal consultation reductions for SP participants, while controls had significantly more visits in a pre-post comparison of annual utilization. In the study by Grant et al. [20], GP utilization in the post-randomization period was identical for intervention and control groups. Another study [84] including a health care utilization module with matched non-responder controls, showed a significant decline in the mean number of primary care visits in a three-month period for the intervention group, while there was no significant longitudinal change in controls. The cRCT by Mercer et al. [92,93] did only report GP utilization data at baseline. Regarding the considerable number of uncontrolled studies on primary care utilization for which data quality did not permit determination of effect sizes, reductions of unclear relevance were reported by many [40,43,56,62,69,70,74,75,76,85], but not all [66,68].

As to emergency department utilization trends in controlled studies, only the case-control study by Carnes et al. [94,95,96] reported relevant data, with a decline in visits in cases and an increase in controls for retrospective 3-month intervals from baseline and from the follow-up point at 8 months. Reported findings from a considerable number of pre-post studies likewise did not permit determination of effect sizes, and results were inconsistent, with pre-post reductions in visits registered by some [40,49,50,54,57,81,97], but not by others [43,55,60,79]. Extractable data was heterogeneous (means, percentages, absolute numbers), with measures of dispersion, confidence intervals, or tests of significance frequently missing (e.g. [55,56,76,77,81]).

## Discussion

### Summary of findings

In our systematic review, we found 68 reports from 53 different projects, with scarce evidence from controlled evaluations. Most frequently studied outcomes represent the domains of mental health and wellbeing, loneliness, quality of life, general health, self-efficacy, and health care utilization. Uncontrolled studies with shorter time frames showed mostly positive effects, while effects reported from studies with control groups or longer follow-up periods were smaller or inconsistent.

### Evaluation of the evidence

The finding that controlled studies show positive effects less frequently calls for caution. SP effects could be non-specific consequences of being enrolled in a study, e.g. the feeling that someone cares. Regression towards the mean [99] might also be a problem because baseline values of certain outcomes (e.g. (S)WEMWBS) are quite low compared to the general public [40,76]. Effects seen could also be partially due to confounding, e.g. unknown concomitant interventions, as well as other sources of bias, e.g. selective dropout or social desirability [100].

As effect sizes could only be calculated for few studies and necessary information was often missing, clinical relevance of the evidence is difficult to judge. The results give reason to question sustainability, with longer-term studies tending to show smaller effects. The positive trends disappearing over time may be due to the complex problems of the target group: people referred to SP may have a plethora of problems related to e.g. low socioeconomic status, chronic illness or age. Temporarily alleviating one or two of the problems by counseling might be less beneficial in the long term, if the underlying destitute social situation remains [101,102].

### Results in context

Our review is generally in line with preceding related works regarding the shortcomings of the available evidence and the need for further high-quality research, while providing the first evidence synthesis on SP quantifying effect sizes and applying methodologically rigorous standards of conduct and reporting. Two evidence syntheses by Bickerdike et al. [23] and Chatterjee et al. [103] have exclusively reviewed evidence from the NHS context, with studies from other settings not eligible for inclusion. Results of these works are presented narratively without attempts at comparative quantification of effects. In the Bickerdike review, 15 evaluations of SP programmes were included, primary outcomes were any measures of health and well-being or usage of health services. All included studies were rated as of high risk of bias, which corresponds to our assessment. Areas of methodological criticism encompassed lack of controls, missing data, and short follow-up, as well as non-standardized measuring instruments and unsatisfactory consideration of potential confounders. Authors consequently judged overall evidence as to the effectiveness of SP as inconclusive. Our findings also largely reflect the Chatterjee et al. review [103], which rated findings as overall positive while putting comparatively less emphasis on methodological shortcomings of the evidence. The risk of bias was only cursorily addressed in this review and not assessed systematically. Reinhardt et al. [21] have recently investigated the effectiveness of SP with loneliness as the outcome of interest, and collaterally addressed the impact on health care utilization. While not formally assessing risk of bias, authors stressed that available studies predominantly lacked control groups. They reported positive effects on loneliness-related score measures and health services usage reductions. Results were not synthesized quantitatively, and no effect sizes were reported. A further current systematic review by Portuguese researchers [104] published after the conclusion of our literature search included 13 studies on the effectiveness of SP. Most reports identified by this review are likewise represented in our literature pool, and authors judged evidence as weak, requiring further research. While our review project goes beyond this recent evidence synthesis regarding the scope of the literature search and the efforts to quantify effect sizes, conclusions essentially are in line with ours.

### Strengths and limitations

For this systematic review, we employed a very comprehensive search strategy considering a wide range of bibliographic databases, performed citation tracking, as well as an extensive screening of the internet and of pertinent repositories for grey literature. By not relying exclusively on formally publicized evidence, this approach fits the special traits of SP research, which so far has been rather centered on pragmatic pilot intervention trials with ensuing report-style evaluations, and in this regard differs markedly from the rigorous conventions of conduct and reporting in clinical research. While the resulting body of literature is more challenging to synthesize and interpret, rejection of the evidence from non-controlled studies and non-peer-reviewed publications would have greatly narrowed the view on SP as it is practiced. Our international search focus also ensured that no evidence was missed on grounds of setting, even if results indicate that SP research is still overwhelmingly UK-centric. Lastly, our approach to compare evidence from different studies by calculation of effect sizes is novel in the field, with preceding reviews limited to mere descriptive summaries.

However, quantification was only possible for a minority of studies, and the necessity to refrain from meta-analysis is certainly a central limitation. While this would have been computationally feasible for the studies with SMD, it would certainly not have been scientifically permissible considering the heterogeneity of the study field. We found no comparative studies of different interventional approaches in the SP context aimed at identifying the most promising concept. As to the great heterogeneity and complexity, it was not possible to differentiate between specific SP models or components [97], although it would be plausible if some schemes would be more effective than others. Data did likewise not permit a substantiated distinction of different levels of intervention integration, with most studies for which effect sizes could be calculated classified as either medium or holistic. We also cannot tell whether other approaches (e.g. integration of social support services into primary care [100]), might be more promising than implementation of SP via a link worker. Neither were social prescribing schemes not involving outpatient medical providers considered in this review. Lastly, SP research is a rapidly evolving field, as indicated by the recentness of a major share of the publications included. Any literature review thus can only provide a snapshot and we may very well learn much more about SP effectiveness in the years to come.

## Conclusion

The evidence located suggests positive effects of SP on a variety of relevant endpoints. Due to prevalent quality deficits in the available studies, scope for conclusions concerning clinical relevance and sustainability is limited. In this context, evidence quality rather than quantity is the problem. SP seems to be a promising integrated care approach for psychosocial problems in the primary care setting. For the UK, NHS policymakers have decided to gradually roll out SP services nationwide [105], which has the potential to broaden the evidence base. Evaluating the effectiveness of complex interventions is a major challenge [106] and requires considerable effort and resources. Recent efforts to address these issues by special techniques, e.g. difference-in-differences analyses using secondary data, are commendable [107]. However, randomized controlled trials with long-term follow-up and efforts to minimize attrition would be the most desirable and valuable approach. In this context, the use of adaptive designs and pragmatic trials might facilitate conducting successful RCTs in the complex field of social prescribing. Additional possible approaches for enhancing feasibility of randomized designs could be e.g. randomization and evaluation of communities and/or practice sites instead of individual patients, or trials including waiting list or stepped wedge designs.

## Additional Files

The additional files for this article can be found as follows:

10.5334/ijic.6472.s1 Appendix 1. Search strategies.

10.5334/ijic.6472.s2 Appendix 2. Studies excluded in full text screening.

10.5334/ijic.6472.s3 Appendix 3. Study characteristics table.

10.5334/ijic.6472.s4 Appendix 4. EPHPP: traffic light plot.
