# Supplementary material for: Social Prescribing: Systematic Review of the Effectiveness of Psychosocial Community Referral Interventions in Primary Care
Source: Int J Integr Care. 2022 Aug 19;22(3):11. doi: 10.5334/ijic.6472 (PMC9389950; doi:10.5334/ijic.6472)
Supplement: Appendix 1. — Search strategies. [file ijic-22-3-6472-s1.pdf]

Appendix 1: Search strategies

Initial search				
**Database**	**Search string, limits**	**Search time frame**	**Search date**	**Results (number of citations)**
**OVID MEDLINE**	**1. social prescri*.mp.<br>2. community referral*.mp.<br>3. social.ti,ab.<br>4. psychosocial.ti,ab.<br>5. referral.ti,ab.<br>6. primary care.ti,ab.<br>7. 3 or 4<br>8. 5 and 6 and 7<br>9. 1 or 2 or 8<br>10. 9 and 2000:2020.(sa_year).**	**2000-2020**	**07.05.2020**	**841**
**OVID EMBASE**	**1. social prescri*.mp.<br>2. community referral*.mp.<br>3. social.ti,ab.<br>4. psychosocial.ti,ab.<br>5. referral.ti,ab.<br>6. primary care.ti,ab.<br>7. 3 or 4<br>8. 5 and 6 and 7<br>9. 1 or 2 or 8<br>10. 9 and 2000:2020.(sa_year).**	**2000-2020**	**07.05.2020**	**1409**
**EBSCO CINAHL**	**(TI "social prescri*" OR AB "social prescri*") OR (TI "community referral*" OR AB "community referral*") OR (((TI psychosocial OR AB psychosocial) OR (TI social OR AB social)) AND (TI referral* OR AB referral*) AND (TI "primary care" OR AB "primary care"))**	**2000-2020**	**07.05.2020**	**793**
**EBSCO SocIndex**	**(TI "social prescri*" OR AB "social prescri*") OR (TI "community referral*" OR AB "community referral*") OR (((TI psychosocial OR AB psychosocial) OR (TI social OR AB social)) AND (TI referral* OR AB referral*) AND (TI "primary care" OR AB "primary care"))**	**2000-2020**	**07.05.2020**	**111**
**Social Care Online**	**social prescribing OR community referral**	**2000-2020**	**07.05.2020**	**397**
**Cochrane:**<br>- **CDSR**<br>- **CENTRAL**	**1. social NEXT prescri*:ti,ab,kw<br>2. community NEXT referral*:ti,ab,kw<br>3. "social":ti,ab,kw<br>4. "psychosocial":ti,ab,kw<br>5: "referral": ti,ab,kw<br>6. "primary care":ti,ab,kw<br>7. 3 or 4<br>8. 5 and 6 and 7<br>9. 1 or 2 or 8<br>10. 9 and Publication Year from (2000 to 2020)**	**2000-2020**	**07.05.2020**	**12 reviews<br>368 trials<br>380 total**
**Google Scholar**	**intitle:"social prescribing"<br><br>Limits: no patents / citations, time frame 2000-2020**	**2000-2020**	**18.05.2020**	**196**
**Update search**				
**Database**	**Search string, limits**	**Search time frame**	**Search date**	**Results (number of citations)**
**OVID MEDLINE**	**1. social prescri*.mp.<br>2. community referral*.mp.<br>3. social.ti,ab.<br>4. psychosocial.ti,ab.<br>5. referral.ti,ab.<br>6. primary care.ti,ab.<br>7. 3 or 4<br>8. 5 and 6 and 7<br>9. 1 or 2 or 8<br>10. 9 and 2020:2021.(sa_year).**	**2020-2021**	**01.02.2021**	**170**
**OVID EMBASE**	**1. social prescri*.mp.<br>2. community referral*.mp.<br>3. social.ti,ab.<br>4. psychosocial.ti,ab.<br>5. referral.ti,ab.<br>6. primary care.ti,ab.<br>7. 3 or 4<br>8. 5 and 6 and 7<br>9. 1 or 2 or 8<br>10. 9 and 2020:2021.(sa_year).**	**2020-2021**	**01.02.2021**	**197**
**EBSCO CINAHL**	**(TI "social prescri*" OR AB "social prescri*") OR (TI "community referral*" OR AB "community referral*") OR (((TI psychosocial OR AB psychosocial) OR (TI social OR AB social)) AND (TI referral* OR AB referral*) AND (TI "primary care" OR AB "primary care"))**	**May 2020- Feb 2021**	**01.02.2021**	**72**
**EBSCO SocIndex**	**(TI "social prescri*" OR AB "social prescri*") OR (TI "community referral*" OR AB "community referral*") OR (((TI psychosocial OR AB psychosocial) OR (TI social OR AB social)) AND (TI referral* OR AB referral*) AND (TI "primary care" OR AB "primary care"))**	**May 2020- Feb 2021**	**01.02.2021**	**5**
**Social Care Online**	**social prescribing OR community referral**	**2020-2021**	**01.02.2021**	**40**
**Cochrane:**<br>- **CDSR**<br>- **CENTRAL**	**1. social NEXT prescri*:ti,ab,kw<br>2. community NEXT referral*:ti,ab,kw<br>3. "social":ti,ab,kw<br>4. "psychosocial":ti,ab,kw<br>5: "referral": ti,ab,kw<br>6. "primary care":ti,ab,kw<br>7. 3 or 4<br>8. 5 and 6 and 7<br>9. 1 or 2 or 8<br>with Cochrane Library publication date from May 2020 to Feb 2021, in Cochrane Reviews and Trials**	**May 2020- Feb 2021**	**01.02.2021**	**30 trials**
**Google Scholar**	**intitle:"social prescribing"<br><br>Limits: no patents / citations, time frame 2020-2021**	**2020-2021**	**01.02.2021**	**68**
