# Supplementary material for: Social Prescribing: Systematic Review of the Effectiveness of Psychosocial Community Referral Interventions in Primary Care
Source: Int J Integr Care. 2022 Aug 19;22(3):11. doi: 10.5334/ijic.6472 (PMC9389950; doi:10.5334/ijic.6472)
Supplement: Appendix 2. — Studies excluded in full text screening. [file ijic-22-3-6472-s2.pdf]

Supplement X: List with all articles excluded after assessment for eligibility

Exclusion reason: Wrong design

1. Abbasi K. There's something about social prescribing. Journal of the Royal Society of Medicine. 2019;112(11): 451–451. https://doi.org/10.1177/0141076819887837.

2. Abernethy H. Promoting Emotional Wellbeing through Social Prescribing. In: Innovations in Stress and Health. Springer; 2011. p. 107–152. https://link.springer.com/chapter/10.1057/9780230321007_6 [Accessed 2nd May 2022].

3. ACTRN12618001970235. The OPTIMISE project: collaborative improvement of primary health care delivery to the Australian refugee community. 2018. https://www.cochranelibrary.com/central/doi/10.1002/central/CN-01949072/full [Accessed 2nd May 2022].

4. ACTRN12620001183976. Stepped Wedge Cluster Randomised Trial of Social Prescribing of Forest Therapy for Adults with Mental Illness. 2020. https://www.cochranelibrary.com/central/doi/10.1002/central/CN-02184588/full [Accessed 2nd May 2022].

5. Alderwick HAJ, Gottlieb LM, Fichtenberg CM, Adler NE. Social Prescribing in the U.S. and England: Emerging Interventions to Address Patients' Social Needs. American Journal of Preventive Medicine. 2018;54(5): 715–718. https://doi.org/10.1016/j.amepre.2018.01.039.

6. Allen C. Social prescribing for primary care in the UK: costs and health benefits are uncertain. 2020. https://evidenceaid.org/resource/social-prescribing-for-primary-care-in-the-uk-costs-and-health-benefits-are-uncertain/ [Accessed 2nd May 2022].

7. Andrew T, Moriarty J, Levin E, Webb S. Outcome of referral to social services departments for people with cognitive impairment. International Journal of Geriatric Psychiatry. 2000;15(5): 406–414. https://doi.org/10.1002/(sici)1099-1166(200005)15:5<406::aid-gps122>3.0.co;2-f.

8. Applebee L, Freeman S, Kinnear K, Parratt J, Omar Y. Response to evaluating student perceptions and awareness of social prescribing...Santoni C, Chiva Giurca B, Li TM, et al. Evaluating student perceptions and awareness of social prescribing. Education for Primary Care, 2019; 30(6): 361-367. Education for Primary Care. 2020;31(1): 60–60. https://doi.org/10.1080/14739879.2019.1689180.

9. Attard J. Unlocking the value of VCSE organisations for improving population health and wellbeing: the commissioners role. London; 2017. https://www.healthylondon.org/wp-content/uploads/2017/10/Unlocking-the-value-of-VCSE-organisations-for-improving-population-health-and-wellbeing.pdf [Accessed 2nd May 2022].

10. Baddeley B, Sornalingam S, Cooper M. Social prescribing in general practice. InnovAiT: Education and inspiration for general practice. 2018;11(2): 119–121. https://doi.org/10.1177/1755738016667967.

11. Baker K, Irving A. Co-producing Approaches to the Management of Dementia through Social Prescribing. Social Policy & Administration. 2016;50(3): 379–397. https://doi.org/10.1111/spol.12127.

12. Banyard R., Jones H.T., Hampshaw S., Dunn A. Primary care. Social skills. Health Serv J. 2002;112(5820): 24–25.

13. Barnes DE. Social prescribing. InnovAiT: Education and inspiration for general practice. 2020;13(11): 638–641. https://doi.org/10.1177/1755738020950340.

14. Bauer D, Batson R, Hayden W, Counts MM. Integrating Behavioral Health Services Within a Primary Care Center in a Rural Setting. Families in Society: Journal of Contemporary Social Services. 2005;86(1): 63–70. https://doi.org/10.1606/1044-3894.1878.

15. Bhanu C. Social prescribing: why a prescription? British Journal of General Practice. 2019; https://bjgp.org/content/69/678/6/tab-e-letters#social-prescribing-why-a-prescription-

16. Bhardwa S. Social prescribing pioneers. Independent Nurse. 2015; 2–2.

17. Bird W, Adamo G, Pitini E, Gray M, Jani A. Reducing chronic stress to promote health in adults: the role of social prescriptions and social movements. Journal of the Royal Society of Medicine. 2020;113(3): 105–109. https://doi.org/10.1177/0141076819890547.

18. Bleacher H, English A, Leblanc W, Dickinson LM. Associations Between Patients' Unmet Social Needs and Self-Reported Health Confidence at One Primary Care Clinic. Journal of Primary Care & Community Health. 2020;11: 1–8. https://doi.org/10.1177/2150132720921329.

19. Bodell S, Lawler C, Martin R. Responding to the social prescribing agenda with occupational science. 2019. https://www.rcot.co.uk/file/3830/download?token=7w5739E7 [Accessed 2nd May 2022].

20. Boyum S, Kreuter MW, McQueen A, Thompson T, Greer R. Getting help from 2-1-1: a statewide study of referral outcomes. Journal of Social Service Research. 2016;42(3): 402–411. https://doi.org/10.1080/01488376.2015.1109576.

21. Bragg R, Egginton-Metters I, Leck C, Wood C. Expanding delivery of care farming services to health and social care commissioners. Natural England commissioned reports. Natural England Commissioned Reports, Number 194; 2015. http://publications.naturalengland.org.uk/publication/5628503589388288 [Accessed 2nd May 2022].

22. Brandling J, House W. Social prescribing in general practice: adding meaning to medicine. British Journal of General Practice. 2009;59(563): 454–456. https://doi.org/10.3399/bjgp09X421085.

23. Brandling J, House W. Investigation into the feasibility of a social prescribing service in primary care: a pilot project. University of Bath and Bath and North East Somerset NHS Primary Care Trust, Bath, U. K.; 2007. https://purehost.bath.ac.uk/ws/portalfiles/portal/426828/Brandling_SocialPrescribingFeasabilityReport.pdf [Accessed 2nd May 2022].

24. Brown M, Friedli L, Watson S. Prescriptions for pleasure. Mental Health Today. 2004; 20–23.

25. Buck D, Ewbanl L. What is social prescribing?. The King´s Fund. https://www.kingsfund.org.uk/publications/social-prescribing [Accessed 2nd May 2022].

26. Campaign to End Loneliness. The missing million: a practical guide to identifying and talking about loneliness. London; 2016. http://www.campaigntoendloneliness.org/wp-content/uploads/CEL-Missing-Millions-Guide_final.pdf [Accessed 2nd May 2022].

27. Cawston P. Social prescribing in very deprived areas. British Journal of General Practice. 2011;61(586): 350–350. https://doi.org/10.3399/bjgp11X572517.

28. Charlton RA, Crompton CJ, Roestorf A, Torry C. Social prescribing for autistic people: A framework for service provision. AMRC Open Research. 2020;2(19): 19. https://doi.org/10.12688/amrcopenres.12901.2.

29. Chartered Society of Physiotherapy. New social prescribing framework for physiotherapy staff hailed as a valuable tool for members. Frontline (20454910). https://www.csp.org.uk/news/2019-07-12-new-social-prescribing-framework-physiotherapy-staff-applauded-csp [Accessed 2nd May 2022].

30. Christie T. Social prescribing - are drugs or people the better cure? Journal of holistic healthcare. 2018;15(3): 59.

31. Clare ICH, Wade KA, Ranke N, Whitson S, Lillywhite A, Jones E, et al. Specialist community teams for adults with learning disabilities: referrals to a countywide service in England. Tizard Learning Disability Review. 2019;24(2): 41–49. https://doi.org/10.1108/TLDR-05-2018-0015.

32. Clements-Cortés A, Yip J. Social Prescribing for an Aging Population. Activities, Adaptation & Aging. 2020;44(4): 327–340. https://doi.org/10.1080/01924788.2019.1692467.

33. Cohen D. NCT04461405: INTEGRATE-D: a Pilot Test to Support Integration of Medical and Psychosocial Care for People With Type II Diabetes. 2020. https://clinicaltrials.gov/ct2/show/NCT04461405 [Accessed 2nd May 2022].

34. Costa A, Mourão S, Santos O, Alarcão V, Virgolino A, Nogueira P, et al. I-DECIDE: A Social Prescribing and Digital Intervention Protocol to Promote Sexual and Reproductive Health and Quality of Life among Young Cape Verdeans. International Journal of Environmental Research and Public Health. 2021;18(3): 850. https://doi.org/10.3390/ijerph18030850.

35. Crompton A. The ‘front door’ to adult social care. Cardiff; 2019. https://www.audit.wales/sites/default/files/Front-door-to-adult-social-care-english_11.pdf [Accessed 2nd May 2022].

36. Davies S. Social prescribing offers options and support. Primary Health Care. 2019;29(3): 12–12. https://doi.org/10.7748/phc.29.3.12.s13.

37. Dayson C, Fraser A, Lowe T. A comparative analysis of Social Impact Bond and conventional financing approaches to health service commissioning in England: the case of social prescribing. Journal of Comparative Policy Analysis: Research and Practice. 2020;22(2): 153–169. https://doi.org/10.1080/13876988.2019.1643614.

38. Dayson C, Painter J, Bennett E. Social prescribing for patients of secondary mental health services: emotional, psychological and social well-being outcomes. Journal of Public Mental Health. 2020;19(4): 271–279. https://doi.org/10.1108/JPMH-10-2019-0088.

39. Dowden A. How social prescribing can benefit patients and prescriber. Prescriber. 2019;30(4): 21–24. https://doi.org/10.1002/psb.1754.

40. Drennan V. Evaluating the use of social prescribing coordinators in general practices. Primary Health Care. 2018;28(1): 13–13. https://doi.org/10.7748/phc.28.1.13.s11.

41. Drinkwater C., Wildman J., Moffatt S. Social prescribing. BMJ (Online). 2019;364: l1285. https://doi.org/10.1136/bmj.l1285.

42. Ernst E. Social prescribing. Perfusion. 2018;31(1): 1.

43. Fancourt D, Opher S, de Oliveira C. Fixed-Effects Analyses of Time-Varying Associations between Hobbies and Depression in a Longitudinal Cohort Study: Support for Social Prescribing? Psychotherapy & Psychosomatics. 2020;89(2): 111–113. https://doi.org/10.1159/000503571.

44. Farenden C, et al. Community navigation in Brighton and Hove: evaluation of a social prescribing pilot. Hove; 2015. https://ihub.scot/media/1656/cn-full-evaluation-nov-2015.pdf

45. Farmer N. Prescribing pills or people: the perplexity of social prescriptions. The British Student Doctor Journal. 2019;3(1): 36–41. https://doi.org/10.18573/bsdj.70.

46. Fixsen A, Polley M. Social prescribing for stress related disorders and brain health. Stress and Brain Health: In Clinical Conditions. 2020;152: 237. https://doi.org/10.1016/bs.irn.2019.11.005.

47. Friedli L. Developing social prescribing and community referrals for mental health in Scotland. Edinburgh; 2007. https://www.webarchive.org.uk/wayback/archive/3000/https://www.gov.scot/Resource/Doc/924/0054752.pdf [Accessed 2nd May 2022].

48. Friedli L, Watson S. Social prescribing for mental heath: briefing for the Northern Centre for Mental Health. Journal of Public Mental Health. 2004;3(2): 46–47.

49. Frostick C, Bertotti M. Social prescribing in general practice. British Journal of General Practice. 2019;69(688): 538–539. https://doi.org/10.3399/bjgp19X706157.

50. Galway K, Forbes T, Mallon S, Santin O, Best P, Neff J, et al. Adapting Digital Social Prescribing for Suicide Bereavement Support: The Findings of a Consultation Exercise to Explore the Acceptability of Implementing Digital Social Prescribing within an Existing Postvention Service. Int J Environ Res Public Health. 2019;16(22): 4561. https://doi.org/10.3390/ijerph16224561.

51. Gibbons AR, Howarth M, Lythgoe A. Social prescribing in Greater Manchester. 2019. https://www.salfordcvs.co.uk/system/files/GM%20Social%20Prescribing%20Research%20-%20Exec%20Summary.pdf [Accessed 2nd May 2022].

52. Goel R, Bloch G. “Prescribing Income”: A Multi-level Approach to Treating Social Determinants for Health Providers. In: Under-Served: Health Determinants of Indigenous, Inner-City, and Migrant Populations in Canada. Toronto: Canadian Scholars; 2018. p. 177. https://books.google.de/books?id=uLZwDwAAQBAJ&pg=PA177&lpg=PA177&dq=%E2%80%9CPrescribing+Income%E2%80%9D:+A+Multi-level+Approach+to+Treating+Social+Determinants+for+Health+Providers&source=bl&ots=CsK6gl1dVP&sig=ACfU3U3koZup6kxNhkXFHtyGnhano0yjaw&hl=de&sa=X&ved=2ahUKEwjn5a3a4cD3AhX_SPEDHaLGB40Q6AF6BAgCEAM#v=onepage&q=%E2%80%9CPrescribing%20Income%E2%80%9D%3A%20A%20Multi-level%20Approach%20to%20Treating%20Social%20Determinants%20for%20Health%20Providers&f=false [Accessed 2nd May 2022].

53. Goodare H. Making social prescriptions mainstream. Journal of the Royal Society of Medicine. 2020;113(1): 4–4. https://doi.org/10.1177/0141076819894873.

54. Gottlieb L., Cottrell E.K., Park B., Clark K.D., Gold R., Fichtenberg C. Advancing social prescribing with implementation science. J. Am. Board Fam. Med. 2018;31(3): 315–321. https://doi.org/10.3122/jabfm.2018.03.170249.

55. Gray M, Adamo G, Pitini E, Jani A. Precision social prescriptions to promote active ageing in older people. Journal of the Royal Society of Medicine. 2020;113(4): 143–147. https://doi.org/10.1177/0141076819865888.

56. Hamilton-West K.E., Gadsby E., Hotham S. Improving the evidence base for social prescribing. BMJ (Online). 2019;364: l744. https://doi.org/10.1136/bmj.l744.

57. Hart O. I008 How social prescribing can support high quality healthcare. 2019. https://academic.oup.com/rheumatology/article/58/Supplement_3/kez109.007/5444486 [Accessed 2nd May 2022].

58. Hindle L. A new framework for social prescribing for AHPS. 2019. https://www.csp.org.uk/system/files/documents/2019-07/a_new_framework_for_social_prescribing_for_ahps_article.pdf [Accessed 2nd May 2022].

59. House W. Whither social prescribing. Journal of holistic healthcare. 15(3): 62.

60. Howarth ML, Donovan H. Social prescribing: the whys, wherefores and implications for nurses & prescribers. Journal of Prescribing Practice. 2019;1(2). https://doi.org/10.12968/jprp.2019.1.2.94.

61. Howarth ML, Lister C. Social prescribing in cardiology: rediscovering the nature. British Journal of Cardiac Nursing. 2019;14(8). https://doi.org/10.12968/bjca.2019.0036.

62. Husk K, Elston J, Gradinger F, Callaghan L, Asthana S. Social prescribing: where is the evidence? British Journal of General Practice. 2019;69(678): 6–7. https://doi.org/10.3399/bjgp19X700325.

63. Hutt P. Social prescribing: A new medicine? InnovAiT: Education and inspiration for general practice. 2017;10(2): 90–95. https://doi.org/10.1177/1755738016682266.

64. Innovation Unit. Wigan community link worker service evaluation. London; 2016. http://www.innovationunit.org/wp-content/uploads/2017/05/Wigan-CLW-service-evaluation.pdf

65. Islam MM. Social Prescribing—An Effort to Apply a Common Knowledge: Impelling Forces and Challenges. Frontiers in Public Health. 2020;8. https://doi.org/10.3389/fpubh.2020.515469.

66. Jani A, Bertotti M, Lazzari A, Drinkwater C, Addarii F, Conibear J, et al. Investing resources to address social factors affecting health: the essential role of social prescribing. Journal of the Royal Society of Medicine. 2020;113(1): 24–27. https://doi.org/10.1177/0141076819865864.

67. Jani A, Gray M. Making social prescriptions mainstream. Journal of the Royal Society of Medicine. 2019;112(11): 459–461. https://doi.org/10.1177/0141076819848304.

68. Jani A, Pitini E, Jungmann S, Adamo G, Conibear J, Mistry P. A social prescriptions formulary: bringing social prescribing on par with pharmaceutical prescribing. Journal of the Royal Society of Medicine. 2019;112(12): 498–502. https://doi.org/10.1177/0141076819877555.

69. Jungmann S, Mistry P, Conibear T, Gray M, Jani A. Using technology - enabled social prescriptions to disrupt healthcare. Journal of the Royal Society of Medicine. 2020;113(2): 59–63. https://doi.org/10.1177/0141076819877541.

70. Kastner M. NCT04437238: Pilot Evaluation of 'KeepWell' Using a Hybrid Effectiveness-Implementation Pragmatic Randomized Controlled Trial. 2020. https://clinicaltrials.gov/ct2/show/NCT04437238 [Accessed 2nd May 2022].

71. Keenaghan C, Sweeney J, McGowan B. Care options for primary care: the development of best practice guidance on social prescribing for primary care teams. 2012. https://www.drugsandalcohol.ie/18852/1/social-prescribing-2012.pdf [Accessed 2nd May 2022].

72. Kimberlee R. What is social prescribing? Advances in Social Sciences Research Journal. 2015;2(1). https://doi.org/10.14738/assrj.21.808.

73. Kimberlee RH. Whither social prescribing? Journal of holistic healthcare. 2018;15(3): 52.

74. Laing K, Steer M, Lawson S, Penn L, O'Brien N, Wildman J. "It was the turning point in my life" How Ways to Wellness social prescribing is improving the health and wellbeing of people with long term conditions. Newcastler University Institute of Health & Society; 2017. https://golab.bsg.ox.ac.uk/documents/Laing_et_al._2017a.pdf [Accessed 2nd May 2022].

75. Lee A, Sundar S. Social prescribing: an essential but neglected component of the undergraduate medical curriculum. Education for Primary Care. 2018;29(6): 385–385. https://doi.org/10.1080/14739879.2018.1514534.

76. Lindsay E. Social prescribing: Leg Clubs - a collaborative example. Br J Community Nurs. 2016;21 Suppl 9(9815827): S40-1. https://doi.org/10.12968/bjcn.2016.21.Sup9.S40.

77. Mahase E. Social prescribing: is it working? BMJ. 2020;368((Mahase) BMJ): m950. https://doi.org/10.1136/bmj.m950.

78. Malone S, Hayes M. Integrated Care Partnerships Northern Ireland - Leading Integration; Delivering Better Outcomes. International Journal of Integrated Care (IJIC). 2017;17: 1–2. https://doi.org/10.5334/ijic.3544.

79. McHale S, Pearsons A, Neubeck L, Hanson CL. Green Health Partnerships in Scotland; Pathways for Social Prescribing and Physical Activity Referral. International journal of environmental research and public health. 2020;17(18): 6832. https://doi.org/10.3390/ijerph17186832.

80. Mendes A. Becoming your own social prescriber. British Journal of Cardiac Nursing. 2021;16(1): 1–1. https://doi.org/10.12968/bjca.2021.0013.

81. Mesman R, Ranke S, Groenewoud S, Heijnders M. [Essential components of Social Prescribing]. Huisarts en Wetenschap. 2020;63(10): 38–45. https://doi.org/10.1007/s12445-020-0850-z.

82. Morris D, Thomas P, Ridley J, Webber M. Community-Enhanced Social Prescribing: Integrating Community in Policy and Practice. International Journal of Community Well-Being. 2020; 1–17. https://doi.org/10.1007/s42413-020-00080-9.

83. Mtemachani T. Commissioning social prescribing. Practice Management. 2018;28(10): 30–33. https://doi.org/10.12968/prma.2018.28.10.30.

84. Munford LA, Wilding A, Bower P, Sutton M. Effects of participating in community assets on quality of life and costs of care: longitudinal cohort study of older people in England. BMJ Open. 2020;10(3): e033186. https://doi.org/10.1136/bmjopen-2019-033186.

85. Nam EW. Social prescribing: Overcome social isolation and depression of COVID-19 outbreak era. Korean Journal of Health Education and Promotion. 2020;37(1): 113–116. https://doi.org/10.14367/kjhep.2020.37.1.113.

86. National Institute for Health and Care Research. 'Care navigation' is being widely adopted in primary care, but in varying ways. National Institute for Health and Care Research.

https://discover.dc.nihr.ac.uk/content/signal-000868/care-navigation-is-being-widely-adopted-in-primary-care-but-in-varying-ways [Accessed 2nd May 2022].

87. Nicola S. Social prescribing within primary care. https://www.macmillan.org.uk/dfsmedia/1a6f23537f7f4519bb0cf14c45b2a629/1813-10061/sharing-good-practice-social-prescribing [Accessed 2nd May 2022].

88. Nunn S. Psychological well-being, physical health and social prescribing in the context of social research. Thorax. 2020;75(7): 536–536. https://doi.org/10.1136/thoraxjnl-2020-214873.

89. Ogden J. Social prescribing in a time of Covid-19 and social isolation…. Progress in Neurology and Psychiatry. 2020;24(3): 4–5. https://doi.org/10.1002/pnp.670.

90. Phizackerley D. Social prescribing: right idea, wrong name? Drug & Therapeutics Bulletin. 2019;57(8): 130. https://doi.org/10.1136/dtb.2019.000046.

91. Pitini E, Adamo G, Gray M, Jani A. Resetting priorities in precision medicine – The role of social prescribing. Journal of the Royal Society of Medicine. 113(8): 310–313. https://doi.org/10.1177/0141076820910325.

92. Polley M, Chatterjee H, Clayton G. Social Prescribing: community-based referral in public health. Perspectives in Public Health. 2017;138(1): 18–19. https://doi.org/10.1177/1757913917736661.

93. Pryke R. Exploring social prescribing options. InnovAiT: Education and inspiration for general practice. 2020; 1755738019888543. https://doi.org/10.1177/1755738019888543.

94. Rafferty C, Santoni C, Beck P, Subramanian A, Chilaka J, Kirtley D, et al. A national conference on social prescribing. The clinical teacher. 2020; https://doi.org/10.1111/tct.13215.

95. Randall K. Social prescribing in practice. Practice Nurse. 2015;45(9): 34–34.

96. Reath J, Trankle S. An early evaluation of an integrated, recovery orientated approach to severe and persisting mental health problems. International Journal of Integrated Care (IJIC). 2016;16(6): 1–2. https://doi.org/10.5334/ijic.3016.

97. Redmond M, Sumner RC, Crone DM, Hughes S. 'Light in dark places': exploring qualitative data from a longitudinal study using creative arts as a form of social prescribing. Arts & Health: International Journal for Research, Policy & Practice. 2019;11(3): 232–245. https://doi.org/10.1080/17533015.2018.1490786.

98. Rhea K.E., Kanne G., Alford C., Black M., Cooper A., Disco M., et al. The duke inter-agency care team: A bridge to geriatric community resources. J. Am. Geriatr. Soc. 2018;66(Supplement 2): S213. https://doi.org/10.1111/jgs.15376.

99. Robinson A. Social prescribing: coffee mornings, singing groups, and dance lessons on the NHS. BMJ. 2018;363: k4857. https://doi.org/10.1136/bmj.k4857.

100. Rojatz D, Haas S, Holzweber L, Nowak P, Rath S, Atzler B. Social Prescribing: Good-Practice-Maßnahme aus dem Starter-Paket zu Krankheitsprävention, Gesundheitsförderung und Gesundheitskompetenz mit und für österreichische Primärversorgungseinheiten aufbereiten. Das Gesundheitswesen. 2020;82(05). https://doi.org/10.1055/s-0040-1709004.

101. Roland M, Everington S, Marshall M. Social prescribing-transforming the relationship between physicians and their patients. The New England journal of medicine. 2020;383(2): 97–99. https://doi.org/10.1056/NEJMp1917060.

102. Salisbury H. Helen Salisbury: Social prescribing and the No 17 bus. BMJ. 2019;364: l271. https://doi.org/10.1136/bmj.l271.

103. Sangraula M, Turner E, Luitel N, van 't Hof E, Shrestha P, Ghimire R, et al. Feasibility of Group Problem Management Plus (PM+) to improve mental health and functioning of adults in earthquake-affected communities in Nepal. 2020;29: e130. https://doi.org/10.1017/S2045796020000414.

104. Santoni C, Chiva Giurca B, Li TM, Mulligan H, Chilaka J, Lazzereschi L, et al. Evaluating student perceptions and awareness of social prescribing. Education for Primary Care. 2019;30(6): 361–367. https://doi.org/10.1080/14739879.2019.1669223.

105. Savage RD, Stall NM, Rochon PA. Reply to Social Prescribing: Creating Pathways Towards Better Health and Wellness...Mulligan K, Bhatti S, Rayner J, et al. Social Prescribing: Creating Pathways Towards Better Health and Wellness. Journal of the American Geriatrics Society (J AM GERIATR S. Journal of the American Geriatrics Society. 2020;68(2): 432–433. https://doi.org/10.1111/jgs.16253.

106. Sims J, Iphofen R. An integrated care pathway for the problems drinker in the community. Drug and Alcohol Professional. 2003;3(1): 24–31.

107. Soraghan CJ, Boyle G, Dominguez-Villoria L, Feighan J, Robinson D. Challenges of implementing a social prescription service in the clinic: Social prescribing in the LAMP project. In: 2015. p. 1–6. https://doi.org/10.1109/ISTAS.2015.7439434.

108. Sriranganathan G. Social prescribing in primary care: An alternative treatment option. Health Science Inquiry. 2014;5(1): 67-page 68.

109. Stevenson C, Wilson I, McNamara N, Wakefield J, Kellezi B, Bowe M. Social prescribing: A practice in need of a theory. British Journal of General Practice. 2019; https://bjgp.org/content/social-prescribing-practice-need-theory#:~:text=The%20more%20urgent%20requirement%20is,activities%20will%20work%20for%20whom.

110. Torjesen I. Social prescribing could help alleviate pressure on GPs. BMJ. 2016;352: i1436. https://doi.org/10.1136/bmj.i1436.

111. Triebwasser JE. NCT04660032: Nudge to Drive Transitions of Care. 2020. https://clinicaltrials.gov/ct2/show/NCT04660032 [Accessed 2nd May 2022].

112. Tyrer P, Boardman J. Refining social prescribing in the UK. The Lancet Psychiatry. 2020;7(10): 831–832. https://doi.org/10.1016/S2215-0366%2820%2930129-2.

113. Wallace AS, Luther B, Jia-Wen Guo, Ching-Yu Wang, Sisler S, Bob Wong, et al. Implementing a Social Determinants Screening and Referral Infrastructure During Routine Emergency Department Visits, Utah, 2017-2018. Preventing Chronic Disease. 2020;17: 1–12. https://doi.org/10.5888/pcd17.190339.

114. Wallace C, Wallace S, Lloyd-Jones N, Davies M, Elliott M, Ganesh S, et al. Enhancing student wellbeing through social prescribing. 2020. http://www.wsspr.wales/resources/WGU%20GCM%20REPORT%2028.08.20%20FINAL.pdf [Accessed 2nd May 2022].

115. Ward A, Asif A, Cattermole R, Chima J, Ebbatson T, Mahi I, et al. Social prescribing by students: the design and delivery of a social prescribing scheme by medical students in general practice. Education for Primary Care. 2020;31(5): 318–322. https://doi.org/10.1080/14739879.2020.1799437.

116. White JM. Social prescribing: the perspectives of service users, providers and prescribers. Glasgow Caledonian University; 2012. https://ethos.bl.uk/OrderDetails.do?uin=uk.bl.ethos.570733 [Accessed 2nd May 2022].

117. White J, Kinsella K, South J. An evaluation of social prescribing health trainers in South and West Bradford. 2010. https://d76388a5-a-62cb3a1a-s-sites.googlegroups.com/site/healthtrainersengland/bradford/BradfordSPHTreport2010.pdf?attachauth=ANoY7cqa6-Rag2oBRn_49atZ8Uq5BVI6yP4z351iFVYl-pm0i1v4zWSj9kCVFqLvaDA1wvQKCKCwImRDiWGwQjzlXTYiEvWUNdeT8VuxXGFd5LwnXmTqLqHjgSccOWUNxDsJhz7iJuTT7S_CpF90Kj9QIPKvIu567iNduVGre0_9ciNnFa7E59HP7imHbqrOXJ42Z0a0iP-4WX1gFwAvJpUueIc7KOMLjTDh_9UlR6v2EAnoXPdN4Z4ESDnHT_VQQBNTn2fZHNXP&attredirects=0 [Accessed 2nd May 2022].

118. Wilkinson EK, Lees A, Weekes S, Duncan G, Meads G, Tapson K. A collaborative, multi-sectoral approach to implementing a social prescribing initiative to alleviate social isolation and enhance well-being amongst older people. Journal of Integrated Care. 2020; https://doi.org/10.1108/JICA-02-2020-0004.

119. Wilson K. Social Prescribing and the Cultural Determinants of Wellbeing in the UK. The Art of Good Health and Wellbeing: 7th Annual International Arts and Health Conference; 2015 Nov 17; Sydney. https://dokumen.tips/documents/social-prescribing-and-the-cultural-determinants-of-wellbeing-in-2016-03-09-social.html [Accessed 2nd May 2022].

120. Yalçın ÖN, Moreno S, DiPaola S. Social Prescribing Across the Lifespan with Virtual Humans. In: 2020. p. 1–3. https://doi.org/10.1145/3383652.3423897.

121. Younan HC, Junghans C, Harris M, Majeed A, Gnani S. Maximising the impact of social prescribing on population health in the era of COVID-19. Journal of the Royal Society of Medicine. 2020;113(10): 377–382. https://doi.org/10.1177/0141076820947057.

Exclusion reason: Wrong intervention

1. Balcazar H., Alvarado M., Hollen M.L., Gonzalez-Cruz Y., Pedregon V. Evaluation of Salud Para Su Corazon (Health for your Heart) -- National Council of La Raza Promotora Outreach Program. Prev Chronic Dis. 2005;2(3): A09.

2. British Red Cross. Tackling loneliness and isolation: findings from the evaluation of our Connecting Communities service. London: British Red Cross; 2019. https://reachandconnect.net/sites/reachandconnect.net/files/Tackling-loneliness-and-isolation-connecting-communities.pdf [Accessed 2nd May 2022].

3. Chan W, Whitford D, Conroy R, Gibney D, Hollywood B. A multidisciplinary primary care team consultation in a socio-economically deprived community: an exploratory randomised controlled trial. BMC health services research. 2011;11: 15. https://doi.org/10.1186/1472-6963-11-15.

4. Eikelenboom N, van Lieshout J, Jacobs A, Verhulst F, Lacroix J, van Halteren A, et al. Effectiveness of personalised support for self-management in primary care: a cluster randomised controlled trial. British journal of general practice. 2016;66(646): e354-61. https://doi.org/10.3399/bjgp16X684985.

5. Fullwood Y. Blended evaluation of Phase 2 of the Age UK Personalised Integrated Care Programme: final evaluation report. London; 2018. https://www.ageuk.org.uk/globalassets/age-uk/documents/programmes/personalised-integrated-care-programme/picp-phase-2-blended-evaluation-report.pdf

6. Harflett N. Ageing Better Isle of Wight: final evaluation report. Bath; 2020. https://www.ndti.org.uk/assets/files/AB-IOW-final-report.pdf [Accessed 2nd May 2022].

7. Howarth M, Rogers M, Withnell N, McQuarrie C. Growing spaces: an evaluation of the mental health recovery programme using mixed methods. Journal of Research in Nursing. 2018;23(6): 476–489. https://doi.org/10.1177/1744987118766207.

8. Ismail K, Stewart K, Ridge K, Britneff E, Freudenthal R, Stahl D, et al. A pilot study of an integrated mental health, social and medical model for diabetes care in an inner-city setting: Three Dimensions for Diabetes (3DFD). Diabetic Medicine. 2020;37(10): 1658–1668. https://doi.org/10.1111/dme.13918.

9. Martín-Borràs C, Giné-Garriga M, Puig-Ribera A, Martín C, Solà M, Cuesta-Vargas A. A new model of exercise referral scheme in primary care: is the effect on adherence to physical activity sustainable in the long term? A 15-month randomised controlled trial. BMJ open. 2018;8(3): e017211. https://doi.org/10.1136/bmjopen-2017-017211.

10. mel research. Evaluation of Leicestershire Local Area Coordination: final report. Birmingham; 2016. https://lacnetwork.org/wp-content/uploads/2018/02/Leicestershire-LAC-Evaluation-Final-Report.pdf [Accessed 2nd May 2022].

11. Minshall C, Castle D, Thompson D, Pascoe M, Cameron J, McCabe M, et al. A psychosocial intervention for stroke survivors and carers: 12 month outcomes of a randomized controlled trial. 2020; https://doi.org/10.1080/10749357.2020.1738677.

12. Miranda J, Siddique J, Belin T, Green BL, Krupnick JL, Chung J, et al. One-Year Outcomes of a Randomized Clinical Trial Treating Depression in Low-Income Minority Women. Journal of Consulting & Clinical Psychology. 74(1): 99–111.

13. Molodynski A, Bolton J, Guest L. Is liaison psychiatry a separate specialty? Comparison of referrals to a liaison psychiatry service and a community mental health team. Psychiatric Bulletin. 2005;29(9): 342–345.

14. Munford LA, Panagioti M, Bower P, Skevington SM. Community asset participation and social medicine increases qualities of life. Social Science & Medicine. 2020;259. https://doi.org/10.1016/j.socscimed.2020.113149.

15. Peabody. Health at home: a new health and wellbeing model for social housing tenants. London; 2018. https://www.housinglin.org.uk/_assets/Resources/Housing/OtherOrganisation/peabody_health-at-home-report_final_optimised.pdf [Accessed 2nd May 2022].

16. Pretty J, Barton J. Nature-based interventions and mind-body interventions: Saving public health costs whilst increasing life satisfaction and happiness. International journal of environmental research and public health. 2020;17(21): 1–23. https://doi.org/10.3390/ijerph17217769.

17. Shannon GR, Wilber KH, Allen D. Reductions in Costly Healthcare Service Utilization: Findings from the Care Advocate Program. Journal of the American Geriatrics Society. 2006;54(7): 1102–1107.

18. Simpson S, Corney R, Fitzgerald P, Beecham J. A randomised controlled trial to evaluate the effectiveness and cost-effectiveness of counselling patients with chronic depression. Health technology assessment (winchester, england). 2000;4(36): 1–83.

19. Tinder foundation. Improving digital health skills in communities: findings from the evaluation of years 1 and 2 of the Widening Digital Participation programme. Sheffield; 2016.

https://nhs.goodthingsfoundation.org/wp-content/uploads/2016/07/Improving_Digital_Health_Skills_Report_2015.pdf [Accessed 2nd May 2022].

20. Tsaroucha A, Kingston P, Stewart T, Walton I, Corp N. Assessing the effectiveness of the 'human givens' approach in treating depression: a quasi experimental study in primary care. Mental Health Review Journal. 2012;17(2): 90–103. https://doi.org/10.1108/13619321211270416.

21. Vest JR, Menachemi N, Grannis SJ, Ferrell JL, Kasthurirathne SN, Zhang Y, et al. Impact of Risk Stratification on Referrals and Uptake of Wraparound Services That Address Social Determinants: A Stepped Wedged Trial. American Journal of Preventive Medicine. 2019;56(4): e125–e133. https://doi.org/10.1016/j.amepre.2018.11.009.

Exclusion reason: Wrong population

1. Bertotti M, Frostick C, Sharpe D, Temirov O. A two-year evaluation of the Young People Social Prescribing (YPSP) pilot. Institute for Connected Communities (ICC), University of East London; 2020. https://repository.uel.ac.uk/download/5c63906437d90e4093a320b51355232d12648ab00d6df93137b1aefa74dfc892/851983/SP%20for%20young%20people%20evaluation%20final%20report%20for%20publication.pdf [Accessed 2nd May 2022].

2. Bird E.L., Biddle M.S.Y., Powell J.E. General practice referral of 'at risk' populations to community leisure services: applying the RE-AIM framework to evaluate the impact of a community-based physical activity programme for inactive adults with long-term conditions. BMC Public Health. 2019;19(1): 1308. https://doi.org/10.1186/s12889-019-7701-5.

3. Giurca BC. Social prescribing student champion scheme: a novel peer-assisted-learning approach to teaching social prescribing and social determinants of health. Education for Primary Care. 2018;29(5): 307–309. https://doi.org/10.1080/14739879.2018.1483211.

4. Harris M. Health Improvement and Prevention Study. 2007. https://anzctr.org.au/Trial/Registration/TrialReview.aspx?ACTRN=12607000423415 [Accessed 2nd May 2022].

5. Jones C, Hartfiel N, Brocklehurst P, Lynch M, Edwards RT. Social return on investment analysis of the health precinct community hub for chronic conditions. International journal of environmental research and public health. 2020;17(14): 1–11. https://doi.org/10.3390/ijerph17145249.

6. Panagioti M, Reeves D, Meacock R, Parkinson B, Lovell K, Hann M, et al. Is telephone health coaching a useful population health strategy for supporting older people with multimorbidity? An evaluation of reach, effectiveness and cost-effectiveness using a 'trial within a cohort'. BMC Medicine. 2018;16(1): N.PAG-N.PAG. https://doi.org/10.1186/s12916-018-1051-5.

7. Pardo A., Violan M., Cabezas C., Garcia J., Minarro C., Rubinat M., et al. Effectiveness of a supervised physical activity programme on physical activity adherence in patients with cardiovascular risk factors. Apunts Med. Esport. 2014;48(182): 37–44. https://doi.org/10.1016/j.apunts.2014.02.001.

8. Prior F., Coffey M., Robins A., Cook P. Long-Term Health Outcomes Associated With an Exercise Referral Scheme: An Observational Longitudinal Follow-Up Study. J Phys Act Health. 2019;16(4): 288–293. https://doi.org/10.1123/jpah.2018-0442.

9. Søgaard R, Sørensen J, Waldorff F, Eckermann A, Buss D, Phung K, et al. Early psychosocial intervention in Alzheimer's disease: cost utility evaluation alongside the Danish Alzheimer's

Intervention Study (DAISY). BMJ Open. 2014;4(1): e004105. https://doi.org/10.1136/bmjopen-2013-004105.

10. Tewari A., Kallakuri S., Devarapalli S., Jha V., Patel A., Maulik P.K. Process evaluation of the systematic medical appraisal, referral and treatment (SMART) mental health project in rural India. BMC Psychiatry. 2017;17(1): 385. https://doi.org/10.1186/s12888-017-1525-6.

Exclusion reason: Wrong outcome

1. Golubinski V, Wild EM, Winter V, Schreyögg J. Once is rarely enough: can social prescribing facilitate adherence to non-clinical community and voluntary sector health services? Empirical evidence from Germany. BMC public health. 2020;20(1): 1–9. https://doi.org/10.1186/s12889-020-09927-4.

2. Maughan DL, Cooke M, Patel A, Parveen T, Braithwaite I, Cook J, et al. Primary-care-based social prescribing for mental health: an analysis of financial and environmental sustainability. Primary Health Care Research & Development (Cambridge University Press / UK). 2016;17(2): 114–121. https://doi.org/10.1017/S1463423615000328.
