# Supplementary material for: Social Prescribing: Systematic Review of the Effectiveness of Psychosocial Community Referral Interventions in Primary Care
Source: Int J Integr Care. 2022 Aug 19;22(3):11. doi: 10.5334/ijic.6472 (PMC9389950; doi:10.5334/ijic.6472)
Supplement: Appendix 3. — Study characteristics table. [file ijic-22-3-6472-s3.pdf]

Supporting Table 2: Study characteristics

<table>
<tr><th>Project</th><th>Methods</th><th>Participants</th><th>Setting</th><th>Interventions</th><th>Outcomes</th><th>Notes</th></tr>
<tr>
<td>Aggar 2020 (15)</td>
<td>Uncontrolled before-and-after study, mixed-methods

Study length:

21 months</td>
<td>Inclusion criteria:

- people aged 18 to 65 years who had been unable to return to work after a work-related injury acquired between six months and three years prior, or who had returned to work on reduced hours or duties, and were living in the general community within the area of greater Sydney, Australia

- assessment by a general practitioner as experiencing psychosocial difficulties and likely to benefit from increased social participation

Exclusion criteria:

- receiving acute inpatient treatment, having significant cognitive impairment, or participating in an alternative program for injured workers

Baseline characteristics:

<table>
<tr><td colspan="2">N</td><td>200</td></tr>
<tr><td>Age</td><td>Mean (SD)</td><td>51 (10.15)</td></tr>
<tr><td>Gender</td><td>Female %</td><td>43.7</td></tr>
<tr><td>Ethnicity</td><td>Neither Aboriginal nor Torres Strait Islander %</td><td>95.7</td></tr>
<tr><td>Living arrangement</td><td>-</td><td>-</td></tr>
<tr><td>Employment status</td><td>Full-time %</td><td>4</td></tr>
<tr><td rowspan="2">Other</td><td>Time in workforce > 10 years % (n=168)</td><td>76.7</td></tr>
<tr><td>Injury-related time off work >2 years % (n=166)</td><td>37.4</td></tr>
</table>

Population: Special (unable to work due to work-related injury)</td>
<td>Primary care: general practice

Country:

Australia (Sydney)</td>
<td>
<table>
<tr><td>Name</td><td>-</td></tr>
<tr><td>Why</td><td>Aims of promoting social and economic participation, increasing psychological wellbeing, and decreasing health service use for injured workers with psychosocial needs</td></tr>
<tr><td>What</td><td>It involved holistic needs assessment, customized care planning, linkage and referral to appropriate locally-based health and social services, enrolment in social and therapeutic activities, follow-up contact

- included art and craft, yoga and relaxation, equine therapy, social groups

- referrals were made to external organizations for services such as financial or relationship counselling, mental health support groups, housing, and other assistance</td></tr>
<tr><td>Who provided</td><td>Qualified and experienced link workers (typically a social worker or similar)</td></tr>
<tr><td>How</td><td>-</td></tr>
<tr><td>Where</td><td>-</td></tr>
<tr><td>When and how much</td><td>12 weeks program</td></tr>
<tr><td>Tailoring</td><td>Personalized through holistic needs assessment</td></tr>
</table>

Intervention: Holistic</td>
<td>Patient outcomes collected by link workers at the program start and upon completion:

- World Health Organization Quality of Life (WHO-QOL-BREF)
- Camberwell Assessment of Need Short Appraisal Schedule (CANSAS)
- EQ-5D-5L Health Thermometer
- Kessler Psychological Distress Scale (K10)
- UCLA 3-item Loneliness Scale
- Pain intensity: Pain Scale
- self-reported current ability to work in paid employment
- Confidence in being able to return to work in the future
- medically approved hours of work
- number of people that participants could count on
- satisfaction with social support, participants that never participated in social activities

Health service utilization collected by link workers at the program start and upon completion:

- having spent time in hospital in the previous three months and at follow-up, frequency of contact with health services
- - Participant claims data including capacity for work at three time Points (Time 1: baseline, Time 2: 12 weeks after baseline/post-intervention; Time 3: 24 weeks after baseline/12 weeks post-intervention)</td>
<td>Peer-Review: Yes

Funding source:

icare Foundation (Insurance and Care NSW)

declaration of interest:

none declared</td>
</tr>
</table>

<table>
<tr>
<td>Aggar 2021 (39)</td>
<td>Uncontrolled before-and-after study

Study length:

-</td>
<td>Inclusion criteria:

- 18–65 years

- living in the community in the Sydney Local Health District

- diagnosed with serious mental illness likely to last 6 months or longer (serious mental illness is one that is severe and persistent, with complex needs)

Exclusion criteria:

- receiving current acute inpatient treatment

- significant cognitive impairment

Baseline characteristics:
<table>
<tr><td>N</td><td></td><td>24</td></tr>
<tr><td>Age</td><td>Mean (SD)</td><td>45 (15)</td></tr>
<tr><td>Gender</td><td>Female %</td><td>69</td></tr>
<tr><td>Ethnicity</td><td>-</td><td>-</td></tr>
<tr><td>Living arrangement</td><td>-</td><td>-</td></tr>
<tr><td>Employment status</td><td>-</td><td>-</td></tr>
<tr><td>Other</td><td>-</td><td>-</td></tr>
</table>

Population: Special (serious mental illness)</td>
<td>Primary care

Country:

Australia (Sydney)</td>
<td>
<table>
<tr><td>Name</td><td>Social prescribing program</td></tr>
<tr><td>Why</td><td>Increase the services provided in the community, improving QoL and Well-being, health self-efficacy, and social economic participation</td></tr>
<tr><td>What</td><td>Arts and crafts group, additional supports and adjustments pre- and post-activities (e.g. arrangements for transportation)</td></tr>
<tr><td>Who provided</td><td>Link worker, groups were led by a practicing artist/instructor and co-facilitated by a mental health social worker</td></tr>
<tr><td>How</td><td>Participants self-presented to their GP and where referred to Plus Social program: assessment, information, referral to activities</td></tr>
<tr><td>Where</td><td>Assessed at home</td></tr>
<tr><td>When and how much</td><td>Weekly 2-3 h for 10 weeks</td></tr>
<tr><td>Tailoring</td><td>- personalized referrals to activities</td></tr>
</table>

Intervention: Holistic</td>
<td>Patient outcomes at baseline (pre-intervention) and 12- or 24-weeks follow-up, assessment at home via social worker:

- Quality of Live: World Health Organisation Quality of Live tool (WHO-QOL-BREF)
- Welfare Needs and Support: Camberwell Assessment of Need Short Appraisal Schedule (CANSAS)
- Health Status and Health Self‑efficacy: EuroQol Health Thermometer (EQ5D)
- Psychosocial Distress: Kessler Psychological Distress Scale (K10)
- Loneliness and Social Participation: UCLA 3-item loneliness Scale
- economic participation (one item measured participation in paid employment (yes/no) in the previous 2 weeks)

Process of care measures at baseline (pre-intervention) and 12- or 24-weeks follow-up, assessment at home via social worker:

- Hospital admissions* (One item measured number of hospital admissions (for any reason) in the previous 6 months</td>
<td>Peer-Review:

Yes

Funding source:

Primary and Community Care Services, NSW, Australia

Declaration of interest:

Author CA has received research grants from Primary and Community Care Services, NSW, Australia. Author JBa is

the CEO of Primary and Community Care Services, NSW, Australia.</td>
</tr>
</table>

<table>
<tr>
<td>Apteligen 2015 (40)</td>
<td>Uncontrolled before-and-after study, mixed-methods

Study length:

21 months</td>
<td>Inclusion criteria:

- those who are not in receipt of care, but at risk of needing it
- those who are in receipt of Council low level support
- people who are socially isolated and who are on the cusp of needing more health and social care interventions

Exclusion criteria:

- meet substantial need threshold and are in receipt of more formal adult social care

Baseline characteristics:
<table>
<tr><td colspan="2">N</td><td>2522</td></tr>
<tr><td>Age</td><td>75 years or over %</td><td>62</td></tr>
<tr><td>Gender</td><td>Female %</td><td>61</td></tr>
<tr><td>Ethnicity</td><td>White british n</td><td>2016</td></tr>
<tr><td>Living arrangement</td><td>-</td><td>-</td></tr>
<tr><td>Employment status</td><td>-</td><td>-</td></tr>
<tr><td>other</td><td>Area where help is needed: “social isolation” n</td><td>630</td></tr>
</table>
Population: General</td>
<td>Primary care

Country:

UK (Buckinghamshire, South East England)</td>
<td>
<table>
<tr><td>Name</td><td>Prevention Matters</td></tr>
<tr><td>Why</td><td>Facilitate access to frontline community services and groups in Buckinghamshire, activating resources, also referred to as ‘social assets’, which are intended to contribute positively to reducing demand for adult social care</td></tr>
<tr><td>What</td><td>Volunteer Hub: recruits volunteers and allocates them to volunteer hosting organisations

Time Credits, which is an approach to getting people involved in community activities in exchange for non-cash rewards worth the equivalent to the time they spend contributing to community activities</td></tr>
<tr><td>Who provided</td><td>14 Community Practice Workers (CPWs) are aligned to the seven GP localities in Buckinghamshire, CPWs are supported by seven Community Links Officers</td></tr>
<tr><td>How</td><td>Step 1: Identification of prevention need, Step 2: referral, Step 3: assessment, Step 4: participation in community services and groups, Step 5: review; builds on a referral system, helps users access frontline community services and groups, including making sure that they are comfortable with their choice

- the needs of the program users are not met by the program itself, but by the frontline community services and groups to which users are referred through the program

- after assessment, the CPW agrees an action plan with the user

- all action plans involve a further referral to a frontline community service or group (e.g. to participate in a lunch club once a month)

- users engage with the CPWs again at the six- and 12-month reviews, after which the user exits the program</td></tr>
<tr><td>Where</td><td>Home visits</td></tr>
<tr><td>When and how much</td><td>initial assessment and again after six and 12 months for a review</td></tr>
<tr><td>Tailoring</td><td>Personalized</td></tr>
</table>
Intervention: Holistic</td>
<td>Patient outcomes at baseline and minimum 6 months follow-up:

- Euroquol Group 5
- Dimension questionnaire (EQ-5D)
- Warwick Edinburgh Mental Wellbeing Scale (WEMWBS)
- Scores in relation to whether program users have enough social contact or feel socially isolated
- -frequency of contact
- Scores in relation to whether program users
- do things they enjoy or value with their time
- number of days within the last week in which they had walked for at least 10 minutes
- -the amount of time they had spent walking on one of those days (hours and minutes)
- general perception of their overall quality of life as a whole (likert scale 1-5)

Process of care measures at baseline and minimum 6 months follow-up:

- -GP visits (monthly for those who visited a GP)
- A&E attendance
- hospital admissions
- Potential cost-benefits*
- Unit cost*
- Cost-benefits*</td>
<td>Peer-Review: no

Funding source:

Buckinghamshire County Council

Declaration of interest:

Not mentioned</td>
</tr>
<tr>
<td>Baines 2015 (41)</td>
<td>Uncontrolled before-and-after study, mixed-methods

Study length: unclear</td>
<td>Inclusion criteria:

- persons who had experienced a recent change in their circumstances (e.g. bereavement or diagnosis of a chronic condition)

- aged over 18

Exclusion criteria:

-patients with high risk or acute mental health conditions were outside the scope of the project

Baseline characteristics:
<table>
<tr><td colspan="2">N</td><td>39</td></tr>
<tr><td>Age</td><td>Range</td><td>25-91</td></tr>
<tr><td>Gender</td><td>Female %</td><td>56.7</td></tr>
</table></td>
<td>Primary care: GP surgeries

Country: UK (Rugby, Warwickshire, West Midlands)</td>
<td>
<table>
<tr><td>Name</td><td>Rugby Social Prescribing Project (Rugby SPP)</td></tr>
<tr><td>Why</td><td>Assist people in addressing underlying societal causes or manage compounding factors of ill-health by unlocking and aligning the many resources and community assets that exist</td></tr>
<tr><td>What</td><td>Mainly referral by GP (from 4 surgeries), choose between two routs: 1. Wellness-Coordinator = intensive support, meeting and assignment to Health Buddies (support and assessment), 2. Navigator = information and signposting only, 707 voluntary and community sector organisations and activities are available for signposting</td></tr>
<tr><td>Who provided</td><td>6 trained volunteer advisors (Navigators), 6 volunteers trained as Health Buddies to assist on a one-to-one basis over a 6 week period</td></tr>
<tr><td>How</td><td>One-to-one, follow up via phone, e-mail</td></tr>
<tr><td>Where</td><td>-</td></tr>
<tr><td>When and How much</td><td>6-week period, Navigator: up to 3 occasions by phone, email or post</td></tr>
<tr><td>Tailoring</td><td>Personalized</td></tr>
</table></td>
<td>Patient outcomes at baseline and 4-weeks follow-up assessed by Health Buddy clients:

- -Warwick Edinburgh Mental Wellbeing Scale (WEMBS)</td>
<td>Peer-Review: No

Funding source:

Conventry & Rugby Clinical Commissioning Group

Declaration of interest: not mentioned</td>
</tr>
</table>

<table>
<tr>
<td></td>
<td></td>
<td>
<table>
<tr><td>Ethnicity</td><td>-</td><td>-</td></tr>
<tr><td>Living arrangement</td><td>-</td><td>-</td></tr>
<tr><td>Employment status</td><td>-</td><td>-</td></tr>
<tr><td>other</td><td>-</td><td>-</td></tr>
</table>
Population: General
</td>
<td></td>
<td>
<table>
<tr><td>Modifications</td><td>Until March 2015 no direct contact with the service provider</td></tr>
</table>
Intervention: Holistic
</td>
<td></td>
<td></td>
</tr>
<tr>
<td>Baker 2017 (42)</td>
<td>Uncontrolled before-and-after study, mixed-methods

Study length: 12 months</td>
<td>
Inclusion criteria: -
Exclusion criteria: -

Baseline characteristics:
<table>
<tr><td colspan="2">N</td><td>15</td></tr>
<tr><td>Age</td><td>Range</td><td>39-84</td></tr>
<tr><td>Gender</td><td>Female %</td><td>80</td></tr>
<tr><td>Ethnicity</td><td>White British %</td><td>100</td></tr>
<tr><td>Living arrangement</td><td>-</td><td>-</td></tr>
<tr><td>Employment status</td><td>-</td><td>-</td></tr>
<tr><td>other</td><td>-</td><td>-</td></tr>
</table>
Population: General
</td>
<td>Primary care

Country:

UK (Wiltshire, South West England)</td>
<td>
<table>
<tr><td>Name</td><td>Artlift Wiltshire Move on Groups (MoG)</td></tr>
<tr><td>Why</td><td>Patients are referred for a range of reasons including: to reduce stress, anxiety or depression; to improve self-esteem or confidence; to increase social networks; alleviate symptom of chronic pain or illness; distract from behaviour related health issues, and improve overall wellbeing and health</td></tr>
<tr><td>What</td><td>It is a primary care based art intervention where health professionals refer NHS patients for an art programme

- the intervention involves attending art sessions delivered by artists working with activities such as words/poetry, ceramics, drawing, mosaics and painting

- patients who have completed Artlift programmes are encouraged to continue with their art activities</td></tr>
<tr><td>Who provided</td><td>Artists</td></tr>
<tr><td>How</td><td>Face-to-face</td></tr>
<tr><td>Where</td><td>Five diverse locations across Wiltshire, primary care setting</td></tr>
<tr><td>When and

How much</td><td>8 weeks, sessions once a week for two hours, upon programme completion patients can be re-referred by a healthcare professional to access another eight week course</td></tr>
<tr><td>Tailoring</td><td>Personalized</td></tr>
</table>
Intervention: Light
</td>
<td>Patient outcomes at baseline, pre-re-referral, post-re-referral, after intervention and follow-up via questionnaire:

- - Warwick Edinburgh Mental Wellbeing Scale (WEMWBS)</td>
<td>Peer-Review: no

Funding source: -

Declaration of interest: not mentioned</td>
</tr>
</table>

<table>
<tr>
<td>Bertotti 2017 (17)</td>
<td>Uncontrolled before-and-after study, mixed-methods

Study length:

11 months</td>
<td>Inclusion criteria:

- patients with a wide range of health and non-health issues including social and economic concerns

Exclusion criteria:-

Baseline characteristics:
<table>
<tr><td colspan="2">N</td><td>100</td></tr>
<tr><td>Age</td><td>Group 45-64 %</td><td>44</td></tr>
<tr><td>Gender</td><td>Female %</td><td>63</td></tr>
<tr><td>Ethnicity</td><td>White %</td><td>44</td></tr>
<tr><td>Living arrangements</td><td>With others %</td><td>59</td></tr>
<tr><td>Employment status n=100</td><td>Employed %</td><td>14</td></tr>
<tr><td>other</td><td>Age left full time education: age 17 and upwards %</td><td>49</td></tr>
</table>
Population: General</td>
<td>Primary Care, Secondary care

Country:

UK (London Borough of Waltham Forest, Greater London)</td>
<td>
<table>
<tr><td>Name</td><td>Social Prescribing Service in Waltham Forest</td></tr>
<tr><td>Why</td><td>Facilitate access to a range of support services that will enable individuals to significantly improve their health and wellbeing

- increase the role of the CVS (Community and voluntary Sector) in the provision of services and evaluate aspects of the service model prior to wider adoption across the borough

- release specialist capacity across the system, so that individuals with the most intense health and social care needs can receive the care they require despite funding constraints

- increase ‘whole system’ efficiency by preventing deterioration in the service user’s condition and by reducing duplication of care between organisations and professions

- provide ‘seamless’ care by placing the service user at the centre of decision-making and designing packages of interventions around their needs irrespective of provider</td></tr>
<tr><td>What</td><td>Social Prescribing Service: part of the Better Care Together Programme</td></tr>
<tr><td>Who provided</td><td>Two social prescribers based at Waltham Forest Borough Council (WFBC)</td></tr>
<tr><td>How</td><td>Social prescriber contact patients over the phone, discussing their needs and aspirations and recommending arange of solutions available in the statutory or community sector</td></tr>
<tr><td>Where</td><td>Telephone only</td></tr>
<tr><td>When and How Much</td><td>Three phone calls to each patient: first phone call to understand clients’ needs and aspirations, second phone call to enable social prescribers to recommend a package of support from statutory or community organisations and a third phone call after eight weeks to assess the patient progress in accessing support services</td></tr>
<tr><td>Tailoring</td><td>Personalized</td></tr>
</table>
Intervention: Medium</td>
<td>Patient outcomes at baseline (pre-intervention) and 4-months follow-up via telephone survey:

- General health (Likert scale 1-5)
- Well-being (Likert scale 0-6)
- EQ-5D-3L
- Warwick Edinburgh Mental Wellbeing
- Scale (WEMWBS)
- Patient Activation Measure (PAM)
- volunteering*
- employment*

Process of care measures at baseline and 4-months follow-up via monitoring data:

- GP visits
- A&E visits

Economic evaluation:

- - SROI</td>
<td>Peer-Review: no

Funding source:

Waltham Forest Borough Council and Waltham Forest Clinical Commissioning Group

Declaration of interest:

Not mentioned</td>
</tr>
<tr>
<td>Bertotti 2020 (a) (43)</td>
<td>Uncontrolled before-and-after study, mixed-methods

Study length:

21 months</td>
<td>Inclusion criteria:

Patients over 18 years presenting with one of the following referral criteria:

• Type-2 Diabetes

• Low level mental health

• Social Isolation

• Carers

Exclusion criteria:-

Baseline characteristics:
<table>
<tr><td colspan="2">N</td><td>182</td></tr>
<tr><td>Age</td><td>< 65 years %</td><td>51.3</td></tr>
<tr><td>Gender</td><td>Female %</td><td>67.4</td></tr>
<tr><td>Ethnicity</td><td>White british %</td><td>40.4</td></tr>
</table></td>
<td>Primary care

Country:

UK (London Borough of Redbridge, Greater London)</td>
<td>
<table>
<tr><td>Name</td><td>Social prescribing service in Redbridge</td></tr>
<tr><td>Why</td><td>Rising tide of long-term conditions, growing health inequalities, improve the health and well-being of patients</td></tr>
<tr><td>What</td><td>To build an action plan towards personal goals and further support from community and statutory services if appropriate; accompanying them to a service, chaperoning, research and language support</td></tr>
<tr><td>Who provided</td><td>Four Social Prescribing Advisors (SPAs) in, to work alongside the two SP Coordinators and four Health and Wellbeing Buddies and Integrative Psychotherapist</td></tr>
<tr><td>How</td><td>GPs refer patients who meet the criteria to a Social Prescribing Coordinator for assessment, the patient is then allocated a Health and Wellbeing Buddy (HWB), they build an action plan towards personal goals and further support from community and statutory services if appropriate</td></tr>
<tr><td>Where</td><td>Via phone, face-to-face</td></tr>
<tr><td>When and How Much</td><td>up to five one-to-one sessions in a 12-week period (although this can be flexible)</td></tr>
<tr><td>Tailoring</td><td>Personalized action plans</td></tr>
</table></td>
<td>Patient outcomes at baseline (pre intervention) and 6-months follow-up collected and inputted by Health and Wellbeing Buddies (HWBs) and Social

Prescribing Advisors (SPA):

- Personal Well-being by the Office for
- National Statistics
- (ONS)
- Visual Analog Scale (EQ-VAS)
- EQ-5D-5L
- Short Warwick Edinburgh Mental Wellbeing
- Scale (SWEMWBS)
- Campaign to End Loneliness tool
- -Measure Yourself Concerns and Wellbeing tool (MYCAW)*
- -social capital* (social networks and support)</td>
<td>Peer-Review: no

Funding source:

Redbridge Borough Council and Redbridge Clinical Commissioning Group have commissioned the Institute for Health and Human Development based at the University of East London (UEL)

Declaration of interest:

Not mentioned</td>
</tr>
</table>

<table>
<tr>
<td></td>
<td></td>
<td>
<table>
<tr><td>Living arrangements</td><td>Alone %</td><td>45.6</td></tr>
<tr><td>Employment status</td><td>Full-time %</td><td>2.3</td></tr>
<tr><td>other</td><td>Carer status: yes %</td><td>20.9</td></tr>
<tr><td></td><td>Long standing physical/mental illness (limited a lot) %</td><td>49.1</td></tr>
</table>
Population: General
</td>
<td></td>
<td>
<table>
<tr><td>Modifications</td><td>Redbridge CVS made changes to their team structure by recruiting four Social Prescribing Advisors (SPAs) in July 2019, to work alongside the two SP Coordinators and four Health and Wellbeing Buddies</td></tr>
</table>
Intervention: Holistic
</td>
<td>Process of Care measures at baseline and 6-months follow-up:
- GP visits
- A&E visits

Economic evaluation:
- - Social Return on Investment (SROI)</td>
<td></td>
</tr>
<tr>
<td>Bertotti 2020 (b) (44)</td>
<td>Uncontrolled before-and-after study

Study length: 14 months</td>
<td>
Inclusion criteria:-

Exclusion criteria:
- service users with a high level of vulnerability
- people facing eviction or with complex mental health problems

Baseline characteristics:
<table>
<tr><td>N</td><td></td><td>166</td></tr>
<tr><td>Age</td><td>Mean</td><td>48</td></tr>
<tr><td>Gender</td><td>Female %</td><td>66.3</td></tr>
<tr><td>Ethnicity</td><td>White british %</td><td>22.1</td></tr>
<tr><td>Living arrangement</td><td>With others (including family) %</td><td>49.1</td></tr>
<tr><td>Employment status</td><td>Full-time %</td><td>9.9</td></tr>
<tr><td>Other</td><td>Educational level: age 20 or over (age when respondent left education) %</td><td>39.4</td></tr>
</table>
Population: Unclear
</td>
<td>Primary care

Country:
UK (London Borough of Hackney and City, Inner London)</td>
<td>
<table>
<tr><td>Name</td><td>Social Prescribing Service in City and Hackney</td></tr>
<tr><td>Why</td><td>Growing pressure on GPs, long term conditions rising, growing health inequalities</td></tr>
<tr><td>What</td><td>Provide time, empathy and crucially support to access non-clinical services offered by the VCSE (Voluntary, Community and Social Enterprise) sector in City and Hackney (a total of 150 organisations in both voluntary and statutory sectors)</td></tr>
<tr><td>Who provided</td><td>Referrals from all 40 GP practices in Hackney and City (East London) to four Social Prescribing Link Workers (SPLWs)</td></tr>
<tr><td>How</td><td>Type of support offered by SPLWs is tailored made to each case and include not just support with managing health problems but, critically, support with social issues such as employment, housing or debt with the view of tackling health inequalities</td></tr>
<tr><td>Where</td><td>-</td></tr>
<tr><td>When and How Much</td><td>Meet SPLWs up to 6 times, for an average session of 30-40 minutes</td></tr>
<tr><td>Tailoring</td><td>Personalized recommendations</td></tr>
<tr><td>Modifications</td><td>Although the majority of referrals come through GP practices, the social prescribing scheme does now also receive referrals from other sources</td></tr>
</table>
Intervention: Holistic
</td>
<td>Patient outcomes at baseline and 3-/6-months follow-up collected by Family Action and the Institute for Health and Human

Development (IHHD):
- EuroQoL (EQ-5D-5L)
- Short Warwick Edinburgh Mental Wellbeing
- Scale (SWEMWBS)

Economic evaluation:
- - Social Return on Investment (SROI)</td>
<td>Peer Review: no

Funding source:
City and Hackney Clinical Commissioning Group, Health Foundation

Declaration of interest:
Not mentioned</td>
</tr>
</table>

<table>
<tr>
<td>Beynon 2020 (45)</td>
<td>Uncontrolled before-and-after study, mixed-methods

Study length:

32 months</td>
<td>Inclusion criteria:

People aged 50+, living in Bristol, social, practical or emotional needs e.g.: Social Isolation, Low level depression/anxiety/stress, Bereavement, Information, advice or guidance, Carers Support, Debt or benefit issues, Housing Issues, Exercise and Healthy Living, Mobility Support

Exclusion criteria:

Aggressive or violent, moderate or advanced dementia, moderate or severe depression or anxiety, otherwise unable to engage or have the ability to make decisions, seeing a CPN, drug or alcohol dependency, seeing a probation officer

Baseline characteristics:
<table>
<tr><td>N</td><td></td><td>1769</td></tr>
<tr><td>Age</td><td>Age group 80-89 %</td><td>27</td></tr>
<tr><td>Gender</td><td>Female %</td><td>63</td></tr>
<tr><td>Ethnicity</td><td>White British</td><td>29</td></tr>
<tr><td>Living arrangement</td><td>-</td><td>-</td></tr>
<tr><td>Employment status</td><td>-</td><td>-</td></tr>
<tr><td>Other</td><td>-</td><td>-</td></tr>
</table>
Population: General</td>
<td>Primary care

Country:

UK (Bristol, South West England)</td>
<td><table>
<tr><td>Name</td><td>Bristol Ageing Better Community Navigators Service</td></tr>
<tr><td>Why</td><td>Improve confidence, boost wellbeing and tackle loneliness & isolation</td></tr>
<tr><td>What</td><td>Referral, initial contact within 5 days. Initial signposting and letter sent where necessary. Coordinator enters details onto Lamplight and informs Navigator of new referral. Navigator contacts client when ready to open case and assesses whether home visit is required. Either simple signposting and 6 month follow-up phone call or Navigator completes risk assessment over the phone if not already complete. Navigator/ Volunteer conducts first home visit and completes Initial Assessment and Form of Authority. BAB forms left with client where appropriate. Referrals made to local services/groups/ organisation. Further home visits, BAB forms collected and signposting conducted. Assistes signposting if needed. Follow-up after 6 months via telephone + interims Feedback form</td></tr>
<tr><td>Who provided</td><td>Delivered by two lead delivery agencies (North Bristol Advice Centre and Bristol Community Health), in partnership with 7 other local organisations, navigators (volunteer or employed): person-centred approach,use of high level communication skills, empathy and patience, appropriate and motivational training is very important, as well as adequate support,reflective practice and debriefing, wide knowledge base and the ability to source up-to-date information about local activities, groups,entitlements and specific agencies, highly skilled in assessing the appropriateness for the service, the level of support needed and whether a referral to another agency is required in order to resolve a particular barrier first before a home visit occurs.</td></tr>
<tr><td>How</td><td>Face-to-face, telephone</td></tr>
<tr><td>Where</td><td>Remote, home visit</td></tr>
<tr><td>When and How Much</td><td>Depending on the needs</td></tr>
<tr><td>Tailoring</td><td>Holistic and individual</td></tr>
</table>
Intervention: Holistic</td>
<td>Patient outcomes at baseline and 6 months follow-up via questionnaire:

- De Jong Gierveld Loneliness Scale
- UCLA Loneliness Scale
- Short Warwick Edinburgh Mental Wellbeing
- Scale (SWEMWBS)
- EQ5D-Index, EQVAS
- Social participation, Volunteering (7 questions)</td>
<td>Peer Review: No

Funding source:

The National Lottery Community Fund Bristol Ageing Better (BAB)

Declaration of interest:

Not mentioned</td>
</tr>
<tr>
<td>Brandling 2011 (16)</td>
<td>Uncontrolled before-and-after study, mixed-methods

Study length:

17 months</td>
<td>Inclusion criteria: -

Exclusion criteria: -

Baseline characteristics:
<table>
<tr><td>N</td><td></td><td>90</td></tr>
<tr><td>Age</td><td>Between 40-70 years %</td><td>42</td></tr>
<tr><td>Gender</td><td>Female %</td><td>76</td></tr>
<tr><td>Ethnicity n=68</td><td>White british</td><td>68</td></tr>
<tr><td>Living arrangement</td><td>-</td><td>-</td></tr>
<tr><td>Employment status</td><td>Unknown n</td><td>72</td></tr>
<tr><td>other</td><td>Disability yes %</td><td>36</td></tr>
</table>
Population: Unclear</td>
<td>Primary care

Country:

UK</td>
<td><table>
<tr><td>Name</td><td>New routes</td></tr>
<tr><td>Why</td><td>Make links between health services and more social opportunities in local communities with a view to benefiting patients, helping patients to make sense of the broad array of support and interest groups available in the community</td></tr>
<tr><td>What</td><td>Health professionals refer patients who wish to enhance their engagement in support and interest groups available in the community</td></tr>
<tr><td>Who provided</td><td>Co-ordinators, The Care Forum, service commissioners</td></tr>
<tr><td>How</td><td>Face-to-face</td></tr>
<tr><td>Where</td><td>-</td></tr>
<tr><td>When and How Much</td><td>First appointment lasts one hour, three to five meetings with patient</td></tr>
<tr><td>Tailoring</td><td>Personalized</td></tr>
</table>
Intervention: Medium</td>
<td>Patient outcomes at baseline (pre intervention) and 6-12-weeks follow-up via questionnaire (postal):

- Warwick Edinburgh Mental Wellbeing
- Scale (WEMWBS)
- Adapted Measure Yourself Medical Outcome Profile (MYMOP)

Process of care measures at baseline (12 months before) and 6 months after via staff at surgery from patient records:

- referrals to secondary health care
- letters to secondary care
- GP/nurse practitioner appointments
- - diagnostic tests</td>
<td>Peer Review: no

Funding source:

Bath and North East Somerset Council

Declaration of interest:

Not mentioned</td>
</tr>
<tr>
<td>Bromley by Bow Centre 2019 (46)</td>
<td>Uncontrolled before-and-after study, mixed</td>
<td>Inclusion criteria: -

Exclusion criteria: -</td>
<td>Primary Care</td>
<td><table>
<tr><td>Name</td><td>Bromley by Bow Centre's Social Prescribing service</td></tr>
<tr><td>Why</td><td>To respond to the high levels of health inequality and deprivation in the local community, aims to support people with non-medical needs and address problems</td></tr>
</table></td>
<td>Patient outcomes at Baseline usually during a Level 2 intervention and 12 weeks/end of intervention</td>
<td>Peer-Review:

No</td>
</tr>
</table>

<table>
<tr>
<td></td>
<td>methods

Study length:

12 months</td>
<td>Baseline characteristics:
<table>
<tr><td colspan="2">N</td><td>583</td></tr>
<tr><td>Age</td><td>Modal age group</td><td>31-40</td></tr>
<tr><td>Gender</td><td>Female %</td><td>65</td></tr>
<tr><td>Ethnicity</td><td>White British %</td><td>-</td></tr>
<tr><td>Living arrangement</td><td>-</td><td>-</td></tr>
<tr><td>Employment status</td><td>-</td><td>-</td></tr>
<tr><td>Other</td><td>Mean number of needs (most common: anxiety/stress/depression/low mood % of all needs)</td><td>4.4 (18.4)</td></tr>
</table>

Population: Unclear</td>
<td>Country:

UK (Bromley-by-Bow, London Borough of Tower Hamlets, East Greater London)</td>
<td><table>
<tr><td></td><td>caused by the wider social determinants of health.</td></tr>
<tr><td>What</td><td>Direct referral – Link Worker directly refers client to service identified by referrer: Client receives no contact from the Link Worker,

Level 1: Telephone assessment and support via onward signposting and referral,

Level 2: Initial face to face session with a Link Worker with coaching, goal setting and further onward signposting and referrals,

Level 3: Two or more face to face sessions with coaching, goal setting and further onward signposting and referrals</td></tr>
<tr><td>Who provided</td><td>A service manager and two 0.4 FTE Social Prescribing Link Workers</td></tr>
<tr><td>How</td><td>Face-to-face, telephone</td></tr>
<tr><td>Where</td><td>-</td></tr>
<tr><td>When and How Much</td><td>Up to six hour-long, one-to-one sessions</td></tr>
<tr><td>Tailoring</td><td>Personalized</td></tr>
</table>

Intervention: Light/Medium</td>
<td>follow-up :

- Measure Yourself Concerns and Wellbeing (MYCaW)</td>
<td>
Funding source:

Tower Hamlets GP Care Group (CIC), Mile End East & Bromley by Bow (MEEBBB) primary care network and Tudor Trust

Declarations of interest:

Unknown</td>
</tr>
</table>

<table>
<tr>
<td>Brown 2018 (13)</td>
<td>Uncontrolled before-and-after study, mixed-methods

Study length:

8 months</td>
<td>Inclusion criteria:

- social isolation/loneliness
- practical support needs (including benefits, letter writing, housing, debt etc.)
- over-reliance on NHS services
- bereavement
- need for improved self-care (e.g. diet and exercise)
- low confidence and self-esteem (including mild-moderate depression/anxiety)
-referrals were also open to any other patients whom GPs/practice staff felt would benefit from non-medical sources of support

Exclusion criteria:

- under the age of 18
- a threat to themselves/others
- in a crisis situation
- suffering from uncontrolled mental health issues or addictions
- suffering from dementia

Baseline characteristics:
<table>
<tr><td>N</td><td></td><td>318</td></tr>
<tr><td>Age</td><td>Mean</td><td>54</td></tr>
<tr><td>Gender</td><td>Female %</td><td>67.3</td></tr>
<tr><td>Ethnicity</td><td>British %</td><td>94</td></tr>
<tr><td>Living arrangement</td><td>-</td><td>-</td></tr>
<tr><td>Employment status</td><td>-</td><td>-</td></tr>
<tr><td>other</td><td>-</td><td>-</td></tr>
</table>

Population: General</td>
<td>Primary care

Country:

UK (Bristol, South West England)</td>
<td>
<table>
<tr><td>Name</td><td>Community Webs</td></tr>
<tr><td>Why</td><td>Improve how health, social care and community services work together, supporting positive mental wellbeing, building community assets, reducing social isolation and ageing better

-provide patients with appropriate support to deal with non-medical issues through coaching and referrals to organisations in the local community (or beyond, as appropriate) and to reduce their use of GP time for non-medical issues</td></tr>
<tr><td>What</td><td>Person-centred supported referral/signposting service</td></tr>
<tr><td>Who provided</td><td>One Project Coordinator with two Community Webs Linkworkers who are based within a cluster of GP practices and receive direct referrals from GPs/practice staff</td></tr>
<tr><td>How</td><td>1. referral to Community Webs from GP practice staff 2. patients meet with Community Webs linkworker (Session 1: guided conversation around patient´s needs/wants/barriers, use motivational interviewing techniques, identity practical support needs, contact relevant organisations, begin to construct action plan of support)

3. sessions 2-4 as needed (construct action plan together, check up wether referrals have been actioned, ensure practical support needs have been/are being met, supported referral to organisations/groups/activities), Community Webs Link-Workers provided support to clients, often to help maintain confidence and commitments to engage with activities in the community</td></tr>
<tr><td>Where</td><td>Face-to-face after appointment by telephone</td></tr>
<tr><td>When and How Much</td><td>3 months, 2 - 4 sessions (each 1-1,5 hours)</td></tr>
<tr><td>Tailoring</td><td>Individual action plan</td></tr>
<tr><td>Modifications</td><td>The project has undergone several changes since it was originally planned. The tender for the project was won by a cluster group of practices in the south of Bristol, Greater Bedminster area. Initially the project was to be managed by a Community Webs Project Lead, employed by the Bristol Clinical Commissioning Group (BCCG), however it was not possible to recruit to the role. It was felt that this was partly due to the dual skill sets required of project management along with patient-facing signposting and navigation experience, which on reflection was considered to be an unlikely skill mix.</td></tr>
</table>
Intervention: Holistic</td>
<td>Patient outcomes at baseline (pre intervention), at exit and 3-months follow-up (via questionnaire):

- De Jong Gierveld Loneliness Scale
- UCLA Loneliness Scale
- Short Warwick Edinburgh Mental Wellbeing Scale (SWEMWBS)
- -MYCAW*</td>
<td>Peer Review: no

Funding source: Bristol Ageing Better (BAB) & Better Care Bristol (BCB), BAB is funded by the Big Lottery Fund

Declaration of interest:

Not mentioned</td>
</tr>
<tr>
<td>Carnes 2017 (94–96)</td>
<td>Non-randomized controlled study, mixed methods

Study length:

25 months</td>
<td>Inclusion criteria:

Intervention: Frequent attender and/or socially isolated, presenting with a social problem, mild-moderate mental health problems.

Control 1: Randomly selected from neighbouring areas not involved in the scheme, older than 23 and younger than 85 old suffering from at least one of the following: depression, anxiety, type 2 diabetes

Control 2: -

Exclusion criteria:

Intervention: Acute crisis, at risk to self and/or others, had uncontrolled addictions or uncontrolled mental health problems, got a care plan in action from another organization, unsuited for group related activities, housebound.

Control 1: Palliative care, housebound</td>
<td>Primary Care, general practices

Country:

UK (London Borough of Hackney and City, Eastern Greater London)</td>
<td>
<table>
<tr><td></td><td>I</td><td>C1</td><td>C2</td></tr>
<tr><td>Name</td><td>Family action social prescribing</td><td>General and mental health, wellbeing and active living</td><td>GP consultation and medication use</td></tr>
<tr><td>Why</td><td>Improve well-being and increase self-efficacy</td><td></td><td></td></tr>
<tr><td>What</td><td>Referral to a Social prescribing coordinator. A mutually determined well-being action plan was devised containing goals for improving patient wellbeing, in some cases this involved referring patients to community organisations and services. If necessary, a volunteer was assigned to help the patient achieve their goals.</td><td>A matched control group from six GP practices in City and Hackney</td><td>GP consultation rates over a two year period (July 2013-June 2015) from a matched control group from participating</td></tr>
</table>
</td>
<td>Patient outcomes at Baseline, 12 weeks and 8 months follow-up via postal questionnaire:

- General health score
- EQ-5D
- Hospital Anxiety and Depression Scale (HADS)
- Wellbeing (past week) (0-6)
- Active engagement in life score (0-20)
- Number of regular activities (0-6)
- Positive and Active Engagement in Life, Health Education Impact Questionnaire (heiQ)</td>
<td>Peer-Review:

Partly (1/3)

Funding source:

City and Hackney Clinical Commissioning Group and The Health Foundation

Declarations of interest:

None declared</td>
</tr>
</table>

<table>
<tr>
<td></td>
<td></td>
<td>Control 2: -

Baseline characteristics:

<table>
<tr><td></td><td></td><td>I</td><td>C1</td><td>C2</td></tr>
<tr><td>N</td><td></td><td>184</td><td>302</td><td>7540</td></tr>
<tr><td>Age</td><td>Median (IQR)</td><td>56 (22)</td><td>58 (20)</td><td>-</td></tr>
<tr><td>Gender</td><td>Female %</td><td>59</td><td>64</td><td>-</td></tr>
<tr><td>Ethnicity</td><td>White British %</td><td>49</td><td>58</td><td>-</td></tr>
<tr><td>Living arrangement</td><td>Living Alone %</td><td>60</td><td>37</td><td>-</td></tr>
<tr><td>Employment status</td><td>Work status Not Paid %</td><td>91</td><td>53</td><td>-</td></tr>
<tr><td>Other</td><td>Education Up to 16 years %</td><td>58</td><td>39</td><td>-</td></tr>
</table>

Population: General</td>
<td></td>
<td>
<table>
<tr><td></td><td></td><td></td><td>practices</td></tr>
<tr><td>Who provided</td><td>Three social prescribing coordinators (SPCs) trained in social work and employed by a third sector organization. Volunteers provided additional support to clients.</td><td></td><td></td></tr>
<tr><td>How</td><td>face-to-face</td><td></td><td></td></tr>
<tr><td>Where</td><td>22 GP surgeries</td><td></td><td></td></tr>
<tr><td>When and How Much</td><td>Up to six sessions with the SPC and as many contacts with the volunteer as required.</td><td></td><td></td></tr>
<tr><td>Tailoring</td><td>Personalised</td><td></td><td></td></tr>
</table>

Intervention: Holistic</td>
<td>Process of care measures at Baseline and 8 months follow-up via postal questionnaire:

- Accident and emergency visits in past 3 months
- Collected electronically and anonymously from patient health care records:
- Annual GP consultation rate before/after referral
- No. of medications (antidepressants, antipsychotics, anxiolytics, nonsteroidal anti-inflammatory drugs and opioid analgesics) 6 months before/after referral

Satisfaction with service at 12 weeks and 8 months follow-up via postal questionnaire</td>
<td></td>
</tr>
<tr>
<td>Crone 2018 (14,47,48)</td>
<td>Uncontrolled before-and-after study

Study length:

-</td>
<td>Inclusion criteria:

Patients could be referred for any of up to seven reasons:
- reduce stress/anxiety/depression
- improve self-esteem/confidence
- improve social networks
- help alleviate symptoms of chronic pain or illness
- distraction from behaviour related health issues
- improve overall wellbeing
- support following loss or major life change

Exclusion criteria: -

Baseline characteristics:

<table>
<tr><td></td><td></td><td>Overall</td><td>Subgroup: Multi-morbid</td></tr>
<tr><td>N</td><td></td><td>1297</td><td>103</td></tr>
<tr><td>Age</td><td>Mean (SD)</td><td>51.1 (15.87)</td><td>53.2 (14.08)</td></tr>
<tr><td>Gender</td><td>Female %</td><td>77.3</td><td>82.5</td></tr>
<tr><td>Ethnicity</td><td>-</td><td>-</td><td>-</td></tr>
<tr><td>Living arrangement</td><td>-</td><td>-</td><td>-</td></tr>
<tr><td>Employment status</td><td>Not working %</td><td>11.9</td><td>51</td></tr>
<tr><td>Other</td><td>-</td><td>-</td><td>-</td></tr>
</table>

Population: General</td>
<td>Primary care

Country:

UK (Gloucestershire, South West England)</td>
<td>
<table>
<tr><td>Name</td><td>Artlift arts-on-referral intervention</td></tr>
<tr><td>Why</td><td>Improve the health and well-being of patients</td></tr>
<tr><td>What</td><td>Range of visual and creative arts were offered (e.g. poetry, ceramics, drawing, mosaic and painting)</td></tr>
<tr><td>Who provided</td><td>8 different artists, mostly within surgeries</td></tr>
<tr><td>How</td><td>Patients were recruited to the intervention by their GP or other health professional, using a specifically designed referral form</td></tr>
<tr><td>Where</td><td>Within surgeries, some were based in community facilities</td></tr>
<tr><td>When and How Much</td><td>8 (10) weeks, group size between 3-10 patients</td></tr>
<tr><td>Tailoring</td><td>-</td></tr>
<tr><td>Modifications</td><td>Duration was 10 weeks from 2009 until August 2013 when it changed to 8 weeks to enable more patients to access the program</td></tr>
</table>

Intervention: Light</td>
<td>Patient outcomes at baseline (week 1) and 8-/10-weeks follow-up (collected by the artists through the anonymised patient referral form):

- Warwick Edinburgh Mental Wellbeing Scale (WEMWBS, but adopted for the study)

Subgroup analysis:

- completers
- partial-, Non-Completers and Non-attenders
- - engaged</td>
<td>Peer-Review: yes (all)

Funding source:

For the completion of this analysis update there has been no external funding sought or received. The original evaluation was funded by NHS Gloucestershire Public Health.

Declaration of interest:

None declared</td>
</tr>
</table>

<table>
<tr>
<td>Dayson 2014

(49,50,54,57,97)</td>
<td>Uncontrolled before-and-after study, mixed-methods

Study length:

31 months</td>
<td>Inclusion criteria:

- especially for patients with long term conditions (focus)

Exclusion criteria:-

Baseline characteristics:

<table>
<tr><td colspan="2">N</td><td>1991</td></tr>
<tr><td>Age</td><td>60 years or over %</td><td>87</td></tr>
<tr><td>Gender</td><td>Female %</td><td>62</td></tr>
<tr><td>Ethnicity</td><td>White British n</td><td>1488</td></tr>
<tr><td>Living arrangement</td><td></td><td>-</td></tr>
<tr><td>Employment status</td><td></td><td>-</td></tr>
<tr><td>Other</td><td></td><td>-</td></tr>
</table>

Population: Special (long term conditions)</td>
<td>Primary care: GP practice

Country:

UK (Rotherham, South Yorkshire, England)</td>
<td>
<table>
<tr><td>Name</td><td>Rotherham Social Prescribing Pilot</td></tr>
<tr><td>Why</td><td>Improving the health and well-being of people from marginalised and disadvantaged groups, reduce costly interventions in specialist care, increase the capacity of GPs to meet the non-clinical needs of patients with complex long term conditions (LTCs) who are the most intensive users of primary care resources</td></tr>
<tr><td>What</td><td>Part of a GP-led Integrated Case Management Pilot, a voluntary and community sector (VCS) liaison service for the whole borough which:
· enables patients and their carers to access support from local VCS organisations contributes a VCS perspective to the assessment of needs and care planning for patients referred to multi-disciplinary Integrated Case Management Teams (ICMTs)
· facilitates the development of new community-based services to fill gaps in provision, and funds additional capacity within existing VCS to meet the increase in demand created by Social Prescribing.</td></tr>
<tr><td>Who provided</td><td>Project manager, five Voluntary and Community Sector Advisors (VCSAs)</td></tr>
<tr><td>How</td><td>VCSAs provide the link between the pilot and multidisciplinary primary care teams, they receive referrals from GP practices of eligible patients and carers and make an assessment of their support needs before referring them on to appropriate VCS services</td></tr>
<tr><td>Where</td><td>First assessment at home</td></tr>
<tr><td>When and How Much</td><td>-</td></tr>
<tr><td>Tailoring</td><td>Personalized recommendations</td></tr>
<tr><td>Modifications</td><td>Duration was 10 weeks from 2009 until August 2013 when it changed to 8 weeks to enable more patients to access the program</td></tr>
</table>

Intervention: Holistic</td>
<td>Patient outcomes at baseline (pre intervention) and 3-4-months follow-up (tool was completed by VCSAs with patients):

Aspects of self-management at baseline (pre intervention) and 3-4-months follow-up

(well-being measurement tool developed specifically for the service):

- feeling positive
- lifestyle
- looking after yourself
- managing symptoms
- work, volunteering and social groups/other activities
- money
- where you live
- family and friends

Process of care measures at baseline (6/12 months before intervention) and 6/12-months follow-up provided by the NHS Data Management and Integration Centre (DMIC):

- number of non-elective inpatient episodes (FCEs)
- number of non-elective continuous inpatient spells
- number of non-elective bed days
- number of A&E attendances

- annual return on investment (ROI) from NHS cost reductions*</td>
<td>Peer-review: no

Funding source:

NHS Rotherham Clinical Commissioning Group

Declaration of interest:

Not mentioned</td>
</tr>
</table>

<table>
<tr>
<td>Dayson 2016 (51)</td>
<td>Uncontrolled before-and-after study, mixed-methods

Study length:

12 months</td>
<td>Inclusion criteria:-

Exclusion criteria:

- acute episodes of psychosis,
or primary issues with drug and alcohol

Baseline characteristics:
<table>
<tr><td>N</td><td></td><td>1058</td></tr>
<tr><td>Age</td><td>60 years or over %</td><td>55</td></tr>
<tr><td>Gender</td><td>Female %</td><td>59.9</td></tr>
<tr><td>Ethnicity</td><td>-</td><td>-</td></tr>
<tr><td>Living arrangement</td><td>-</td><td>-</td></tr>
<tr><td>Employment status</td><td>-</td><td>-</td></tr>
<tr><td>Other</td><td>Disability yes n</td><td>649</td></tr>
</table>

Population: Special (long term conditions)</td>
<td>Primary care

Country:

UK (Doncaster, South Yorkshire, England)</td>
<td>
<table>
<tr><td>Name</td><td>Doncaster Social Prescribing Service</td></tr>
<tr><td>Why</td><td>It aims to prevent worsening health for people with long term health conditions and reduce the number and intensity of costly interventions in urgent or specialist care.</td></tr>
<tr><td>What</td><td>Service's Advisors receive client referrals from GPs, community nurses and pharmacists and provide them with support to access a range of voluntary, community and statutory services that meet their needs</td></tr>
<tr><td>Who provided</td><td>Service's Advisors, a number of volunteers that support the delivery of the Service</td></tr>
<tr><td>How</td><td>Face-to-face</td></tr>
<tr><td>Where</td><td>Home visit</td></tr>
<tr><td>When and How Much</td><td>10-15 minutes for each home visit</td></tr>
<tr><td>Tailoring</td><td>Tailored plan</td></tr>
<tr><td>Modifications</td><td>The nature of referrals had changed since the pilot phase: cases had become more complex, often with multiple issues which need a number of individual referrals. There were more clients with severe or complex mental health issues</td></tr>
</table>

Intervention: Medium</td>
<td>Patient outcomes at baseline (pre intervention) and 3-6-months follow-up (monitoring data and self-evaluation questionnaires):

- Doncaster Outcome Tool (DOT) (includes: health: EQ-5D-3L*, social connectedness/contact: 'social isolation and loneliness' scale, financial management: scale from Office for National Statistics-ONS)

Process of care measures (collected by SYHA and Doncaster CVS):

- GP appointments*
- Nurse appointments*
- mental health* services*
- psychotherapy appointments*
- A&E attendance*
- Inpatient stays*

- contact with social worker*</td>
<td>Peer-Review: no

Funding source:

Community Fund Prospectus Innovation Fund, Better Care Fund

Declaration of interest:

Not mentioned</td>
</tr>
</table>

<table>
<tr>
<td>Dayson 2017

(52,53,58)</td>
<td>Uncontrolled before-and-after study, mixed-methods

Study length:

24 months</td>
<td>Inclusion criteria:

The service focusses on three service user care pathway clusters:
· Cluster 4: severe depression and/or anxiety and/or other non-psychotic disorders that increase the complexity of their needs
· Cluster 7: issues associated with long term anxiety and depression or other non-psychotic disorders. They will have received treatment for a number of years and although their symptoms are improved and stable, as a result of long term ill-health they are likely to have a level of social disability that affects their day to day functioning, and leads them to be over dependent on others.
· Cluster 11: a history of psychotic symptoms that are currently controlled and causing minor problems if any at all. They are likely to be experiencing a sustained period of recovery, but require support to regain confidence with day to day life skills, such as sustaining meaningful relationships, and re-entering the work place. They may also have some long term dependence issues.

Exclusion criteria:-

Baseline characteristics:
<table>
<tr><td>N</td><td>317</td></tr>
<tr><td>Age</td><td>-</td></tr>
<tr><td>Gender</td><td>-</td></tr>
<tr><td>Ethnicity</td><td>-</td></tr>
<tr><td>Living arrangement</td><td>-</td></tr>
<tr><td>Employment status</td><td>-</td></tr>
<tr><td>Other</td><td>-</td></tr>
</table>

Population: Special

(psychiatric diagnosis)</td>
<td>Primary care

Country:

UK (Rotherham, South Yorkshire, England)</td>
<td>
<table>
<tr><td>Name</td><td>Rotherham Social Prescribing Mental Health Service</td></tr>
<tr><td>Why</td><td>To help people with mental health conditions overcome the barriers which prevent discharge from secondary mental health care services, to create opportunities for mental health service users to sustain their health and well-being outside secondary mental health services, to create capacity within secondary mental health services, to improve efficiency within mental health services</td></tr>
<tr><td>What</td><td>Phase 1 (typically weeks 1 – 10): Referrals are made for individual service users by an RDaSH practitioner.

Phase 2 (typically weeks 11 – 18): A discharge review meeting is normally held between the RDaSH practitioner, the VCS Advisor and the service user to assess progress with social prescribing.

Phase 3 (typically weeks 19 – 26): During this phase, the service user's primary contact is the VCS Advisor or one of the voluntary sector organisations providing support through the programme.

Range of activities:

· Befriending plus, providing peer support for people lacking confidence to engage in community activities independently (five providers), including two services to support engagement in physical activity and one for vulnerable women

· Education and training opportunities, enabling people to build practical skills and confidence in areas of interest including working towards employment.· Community activity groups, providing opportunities for people to engage social activities base around a particular hobby or interest (five providers)

· Therapeutic services, enabling people to develop relaxation and mindfulness techniques in a supportive environment</td></tr>
<tr><td>Who provided</td><td>VCS Advisor</td></tr>
<tr><td>How</td><td>One-to-one</td></tr>
<tr><td>Where</td><td>-</td></tr>
<tr><td>When and How Much</td><td>Six months pathway</td></tr>
<tr><td>Tailoring</td><td>Personalized</td></tr>
</table>
Intervention: Holistic</td>
<td>Patient outcomes at baseline (pre intervention) and 4-6 months follow-up (baseline collected by VCSAs and follow-up via telephone):

Well-being measures (for each five point scale):

- where you live
- money
- work, volunteering and social groups
- managing symptoms
- looking after yourself
- lifestyle
- feeling positive
- family and friends
- Wider outcomes social benefits:
- employment*
- training and education*
- volunteering*
- physical activity*
- peer support*
- voluntary and community sector activities*

Economic evaluation:

- -Social return on Investment (SROI)*</td>
<td>Peer-Review: no

Funding source:

NHS Rotherham Clinical Commissioning Group

Declaration of interest: not mentioned</td>
</tr>
<tr>
<td>Dayson 2018 (56)</td>
<td>Uncontrolled before-and-after study, mixed-methods

Study length:

9 months</td>
<td>Inclusion criteria:

- patients who could benefit from additional socially focused support, including people who are isolated, feeling low or lacking in confidence

Exclusion criteria: -

Baseline characteristics:
<table>
<tr><td>N</td><td></td><td>703</td></tr>
<tr><td>Age</td><td>< 65 years %</td><td>78</td></tr>
<tr><td>Gender</td><td>Female %</td><td>69</td></tr>
<tr><td>Ethnicity</td><td>White British %</td><td>47</td></tr>
<tr><td>Living arrangement</td><td>-</td><td>-</td></tr>
<tr><td>Employment status</td><td>-</td><td>-</td></tr>
<tr><td>Other</td><td>Service users with at least one long-term</td><td>77</td></tr>
</table></td>
<td>Primary care

Country:

UK (Bradford, West Yorkshire, England)</td>
<td>
<table>
<tr><td>Name</td><td>Community Connectors Social Prescribing Service</td></tr>
<tr><td>Why</td><td>Improve the health, well-being and social connectedness of local people, reduce unplanned and unnecessary demand on primary and secondary health service</td></tr>
<tr><td>What</td><td>Referral from a GP of patients, followed by a home visit from a Community Connector during which they find out what people are interested in, identify what services and activities are available locally that fit those interests, and then help people access them

-if a patient is lacking in confidence the Connector will go along with them to a service or activity until they are confident enough to go on their own</td></tr>
<tr><td>Who provided</td><td>Community Connector</td></tr>
<tr><td>How</td><td>Face-to-face</td></tr>
<tr><td>Where</td><td>Home visit or meeting in a mutually agreed setting</td></tr>
<tr><td>When and How Much</td><td>-</td></tr>
</table></td>
<td>Patient outcomes at baseline (pre intervention) and 3-months follow-up (questionnaire completed with Community Connector services):

- EQ-VAS scale
- EQ-5D*
- Short Warwick Edinburgh Mental Well-being Scale (SWEMWBS)
- Trust (Likert scale 0-10)
- Connectedness and relationship*
- self-care*

Process of care measures 3 months before and 3/6 months after:</td>
<td>Peer-Review: no

Funding source:

Not mentioned

Declaration of interest:

Not mentioned</td>
</tr>
</table>

<table>
<tr>
<td></td>
<td></td>
<td>
<table>
<tr><td></td><td>condition %</td><td></td></tr>
<tr><td></td><td>Most common long-term condition: depression %</td><td>47</td></tr>
</table>
Population: General
</td>
<td></td>
<td>
<table>
<tr><td></td><td></td></tr>
<tr><td>Tailoring</td><td>Personalized recommendations</td></tr>
</table>
Intervention: Holistic
</td>
<td>- Number of Accident and Emergency Community Connector service users
- - Number of GP engagements by Community Connector service users</td>
<td></td>
</tr>
<tr>
<td>Dayson 2020 (a) (55)</td>
<td>Uncontrolled before-and-after study

Study length:

36 months</td>
<td>
Inclusion criteria: -

Exclusion criteria: -

Baseline characteristics:
<table>
<tr><td>N</td><td></td><td>1730</td></tr>
<tr><td>Age</td><td>Age group 80-89 %</td><td>98</td></tr>
<tr><td>Gender</td><td>Female %</td><td>60</td></tr>
<tr><td>Ethnicity</td><td>White British %</td><td>98</td></tr>
<tr><td>Living arrangement</td><td>-</td><td>-</td></tr>
<tr><td>Employment status</td><td>-</td><td>-</td></tr>
<tr><td>Other</td><td>-</td><td>-</td></tr>
</table>
Population: Unclear
</td>
<td>Primary care

Country:

UK (Rotherham, South Yorkshire, England)</td>
<td>
<table>
<tr><td>Name</td><td>Rotherham Social Prescribing Service (RSPS)</td></tr>
<tr><td>Why</td><td>To increase the capacity of GPs to meet the non-clinical needs of patients with complex long-term conditions (LTCs) who are the most intensive users of primary care resources</td></tr>
<tr><td>What</td><td>GP-led Integrated Case Management. It is delivered by Voluntary Action Rotherham (VAR) in partnership with more than 20 local voluntary and community organisations (VCOs). Specific support for the carers of case-managed patients is also provided voluntary and community sector (VCS) liaison service for the whole borough which: Enables patients and their carers to access support from local VCS organisations. Contributes a VCS perspective to the assessment of needs and care planning for patients referred to multi-disciplinary Integrated Case Management Teams (ICMTs). Facilitates the development of new community-based services to fill gaps in provision, and funds additional capacity within existing VCS to meet the increase in demand created by RSPS. make an assessment of their support needs before referring them on to appropriate VCS services.</td></tr>
<tr><td>Who provided</td><td>Core team consisting of a Service Manager and seven Voluntary and Community Sector Advisors (VCSAs)</td></tr>
<tr><td>How</td><td>-</td></tr>
<tr><td>Where</td><td>Home visit</td></tr>
<tr><td>When and How Much</td><td>-</td></tr>
<tr><td>Tailoring</td><td>Personalized</td></tr>
</table>
Intervention: Holistic
</td>
<td>Patient outcomes at baseline and 4-6 months after referral via questionnaire:

- Wellbeing outcome tool (eight components)

Process of care measures at 12 months before and 12 months after referral:

- Number of non-elective inpatient spells
- Number of A&E attendances</td>
<td>Peer-Review: no

Funding source:

NHS Rotherham Clinical Commissioning Group (CCG)

Declaration of interest: not mentioned</td>
</tr>
</table>

<table>
<tr>
<td>Dayson 2020 (b) (59)</td>
<td>Uncontrolled before-and-after study, mixed-methods

Study length:

18 months</td>
<td>Inclusion criteria:

- patients who could benefit from additional socially focused support, including people who are isolated, feeling low or lacking in confidence and those may need support to access other services in their community such as benefits advice, housing, bereavement and mental health services

Exclusion criteria:-

Baseline characteristics:
<table>
<tr><td colspan="2">N</td><td>1984</td></tr>
<tr><td>Age</td><td>< 65 years %</td><td>30</td></tr>
<tr><td>Gender (n=1329)</td><td>Female %</td><td>69</td></tr>
<tr><td>Ethnicity</td><td>White British %</td><td>51</td></tr>
<tr><td>Living arrangement</td><td>-</td><td>-</td></tr>
<tr><td>Employment status</td><td>-</td><td>-</td></tr>
<tr><td rowspan="2">Other</td><td>Referral reason anxiety and low mood %</td><td>53</td></tr>
<tr><td>Service users with at least one long-term condition %</td><td>67</td></tr>
</table>

Population: General</td>
<td>Primary care

Country:

UK (Bradford, West Yorkshire, England)</td>
<td>
<table>
<tr><td>Name</td><td>Community Connectors Social Prescribing Service</td></tr>
<tr><td>Why</td><td>Improve the health, well-being and social connectedness of local people, reduce unplanned and unnecessary demand on primary and secondary health services</td></tr>
<tr><td>What</td><td>Starts with a referral from a GP or other practice staff or healthcare professionals (including the Virtual Ward at Accident and Emergency) via System One or Care Navigation, followed by a home visit or meeting in a mutually agreed setting with a Community Connector who will work alongside the individual to establish what support is required and what they are interested in

- Community Connector would then help identify what services and activities are available locally that fit those interests and support the individual to access them</td></tr>
<tr><td>Who provided</td><td>Community Connector</td></tr>
<tr><td>How</td><td>Face-to-face</td></tr>
<tr><td>Where</td><td>Home visit</td></tr>
<tr><td>When and How Much</td><td>Usually offered for up to six sessions of up to an hour, although in some cases support has been provided over an extended period if it was believed that this would help sustain lasting change</td></tr>
<tr><td>Tailoring</td><td>Personalized recommendations</td></tr>
</table>

Intervention: Holistic</td>
<td>Patient outcomes at baseline (pre intervention) and endpoint (app. after 3 months) via monitoring data and questionnaire:

- EQ-VAS
- EQ-5D
- Short Warwick Edinburgh Mental Well-being Scale (SWEMWBS)
- - Trust (Likert scale 0-10)</td>
<td>Peer-Review: no

Funding source:

Not mentioned

Declaration of interest: not mentioned</td>
</tr>
</table>

<table>
<tr>
<td>Elston 2019 (60)</td>
<td>Uncontrolled before-and-after study

Study length: 12 months</td>
<td>Inclusion criteria:
- aged 50 years or over
- two or more long-term conditions
- considered as likely to benefit from a social intervention
Exclusion criteria: -
Baseline characteristics:
<table>
<tr><td>N</td><td></td><td>1046</td></tr>
<tr><td>Age</td><td>Mean</td><td>79.6</td></tr>
<tr><td>Gender (n=86)</td><td>Female %</td><td>73.3</td></tr>
<tr><td>Ethnicity</td><td>-</td><td>-</td></tr>
<tr><td>Living arrangement</td><td>-</td><td>-</td></tr>
<tr><td>Employment status</td><td>-</td><td>-</td></tr>
<tr><td>other</td><td>-</td><td>-</td></tr>
</table>
Population: Special (long-term conditions)</td>
<td>Primary care setting, community care, secondary care

Country:
UK (South Devon, Devon, South West England)</td>
<td>
<table>
<tr><td>Name</td><td>social prescribing ‘holistic’ link-worker</td></tr>
<tr><td>Why</td><td>Improve health and social wellbeing, patient activation and frailty levels, and that this would lead
to less use of primary, social and acute care services and reduced costs
- secondary objective was to explore what patient characteristics on programme entry were associated with positive outcomes</td></tr>
<tr><td>What</td><td>Initial 30–40 min strengths-based, guided conversation with all referrals, mostly in their homes (80%), to determine need and decide whether signposting, a short conversation or a more in-depth ‘holistic’ conversation is required</td></tr>
<tr><td>Who provided</td><td>Co-ordinators employed by 7 key voluntary sector organisations, embedded in local communities across the area
- Co-ordinators are based in a variety of settings, including NHS premises
- all Co-ordinators are non-health care staff (although some previously worked in the health service) and all received training in goal setting, use of tools and outcome measures, and in how to engage with users in a strengths-based way, co-produce a plan and manage risk
- key aspects of the role included: listening skills, emotional support, advice and practical assistance and coaching</td></tr>
<tr><td>How</td><td>Referrals were received mainly from statutory services – GPs, community and social care staff in multidisciplinary meetings, hospital discharge staff (acute and community) and housing staff – as well as local voluntary and community organisations
- Co-ordinators hold an initial 30–40 min strengths-based, guided conversation with all referrals, mostly in their homes (80%), to determine need and decide whether signposting, a short conversation or a more in-depth ‘holistic’ conversation is required, resilience-focused coaching and practical support and advocacy to navigate and access local health, social and economic services</td></tr>
<tr><td>Where</td><td>In a setting preferred by the person, mostly participants homes (80%)</td></tr>
<tr><td>When and How Much</td><td>Up to 12 weeks</td></tr>
<tr><td>Tailoring</td><td>Personalized</td></tr>
</table>
Intervention: Holistic</td>
<td>Patient outcomes at baseline (collected at either the first or second meeting with the Co-ordinator) and 12-weeks follow-up or point of exit:
- Warwick Edinburgh Mental Well-being Scale (WEMWBS)
- Well-being Star
- Patient Activation Measure (PAM)
- Rockwood Clinical Frailty Scale (RCFS)
- goal achievement

Process of care measures 12 months before and 12 months after intervention (from local IT systems):
- A&E visits and average costs
- in-patient visits and average costs
- outpatient visits
- community visits
- social care visits
- health costs
- social care costs
- total costs

(cost analysis was based on health and community service attendance 12 months before and after the intervention, it used the Trust’s attendance costs submitted to the National Tariff Payment System for 2016–17)</td>
<td>Peer-Review: yes

Funding source:
Torbay Medical Research Fund and Torbay and South Devon NHS Foundation Trust (TSDFT): TMRF project number 120

Declaration of interest:
This evaluation was supported by Torbay MedicalResearch Fund and Torbay and South Devon NHS Foundation Trust(TSDFT). TSDFT also commissioned the SP service (managed by Sue Wroeand Helen Harman) and part-funded the Researchers-in-Residence (JulianElston and Felix Gradinger) with Torbay Medical Research Fund (TMRFproject number 120). Julian Elston and Felix Gradinger are employed byPlymouth University.</td>
</tr>
<tr>
<td>ERS 2013 (61)</td>
<td>Uncontrolled before-and-after study, mixed-methods

Study length:
15 months</td>
<td>Inclusion criteria: people with long-term conditions
Exclusion criteria:-
Baseline characteristics:
<table>
<tr><td>N</td><td></td><td>124</td></tr>
<tr><td>Age</td><td>< 61 years %</td><td>71</td></tr>
<tr><td>Gender</td><td>Female %</td><td>70</td></tr>
<tr><td>Ethnicity</td><td>White British %</td><td>76</td></tr>
<tr><td>Living arrangement</td><td>-</td><td>-</td></tr>
<tr><td>Employment status</td><td>-</td><td>-</td></tr>
<tr><td>other</td><td>-</td><td>-</td></tr>
</table></td>
<td>Primary care

Country:
UK (Newcastle upon Tyne, Northern England)</td>
<td>
<table>
<tr><td>Name</td><td>Newcastle Social Prescribing Project</td></tr>
<tr><td>Why</td><td>Enable health professionals to refer (vulnerable) people with Long Term Conditions to non-clinical community services and networks
- raise awareness and equip health professionals to be able to use the service through roll out of care planning training and support to all local practices
- develop and maintain a comprehensive web-based information resource to support the project</td></tr>
<tr><td>What</td><td>The linkworker role was to support patients referred with:
- personalised goal setting and buddying
- selfcare so that people can self-care/self- manage their condition
- signposting to sources of information, advice and support</td></tr>
<tr><td>Who provided</td><td>Strategic Link worker, project Coordinator, project Manager
In addition other voluntary and community sector organisations and networks were involved in the project. The Quality of Life Partnership, HAREF and Involve North East supported the engagement of local people in the project.</td></tr>
</table></td>
<td>Patient outcomes at baseline (pre intervention) and after intervention:
- - Short Warwick Edinburgh Mental Well-being Scale (SWEMWBS)</td>
<td>Peer-Review: no

Funding source:
Involve North East
NESTA
(National Endowment for Science, Technology and the Arts)

Declaration of interest:
not mentioned</td>
</tr>
</table>

		Population: Special (long term conditions)		<table><tr><td>How</td><td>One-to-one</td></tr><tr><td>Where</td><td>-</td></tr><tr><td>When and How Much</td><td>-</td></tr><tr><td>Tailoring</td><td>Personalized</td></tr><tr><td>Modifications</td><td>The delivery model outlined in the initial NESTA application with a lead organisation managing a 3 person project team was not delivered<br><br>- the strategic link worker role was to be provided by a project team member, it was agreed the project link worker function could be delivered through the consortium of existing providers</td></tr></table><br>Intervention: Holistic		

<table>
<tr>
<td>Ferguson 2018 (62)</td>
<td>Uncontrolled before-and-after study, mixed-methods

Study length:

8 months</td>
<td>Inclusion criteria:

- clients who are registered with a Tower Hamlets GP have expressed a non-clinical support need and
- aged 18 and over (with Network 1 also seeing clients aged 16-18)
- parents with issues related to their children

Exclusion criteria:-

Baseline characteristics:

<table>
<tr><td colspan="2">N</td><td>2270</td></tr>
<tr><td>Age</td><td>Age group: 30-64 %</td><td>70</td></tr>
<tr><td>Gender</td><td>Female %</td><td>60</td></tr>
<tr><td>Ethnicity</td><td>White %</td><td>23</td></tr>
<tr><td>Living arrangement</td><td>-</td><td>-</td></tr>
<tr><td>Employment status</td><td>-</td><td>-</td></tr>
<tr><td>other</td><td>Clients with at least one long-term health condition %</td><td>52</td></tr>
</table>

Population: General</td>
<td>Primary care

Country:

UK (London Borough of Tower Hamlets, East London)</td>
<td>
<table>
<tr><td>Name</td><td>Tower Hamlets social precribing</td></tr>
<tr><td>Why</td><td>Residents in Tower Hamlets experience poorer health outcomes than the general population
- in deprived areas patients often visit their GP for reasons other than clinical problems as a result of not knowing where to get support for their wider social issues, which have a significant, negative impact on their health</td></tr>
<tr><td>What</td><td>Social prescribing scheme

- needs are discussed during the course of the consultation and referrals or sign-posts are made to organisations which can support those needs

- majority of support organisations are in the voluntary and community sector or informal support groups, although some referrals are made to statutory organisations</td></tr>
<tr><td>Who provided</td><td>10 Social Prescribers
- 1 Social Prescriber in each of the GP Networks (8 networks, overall 37 GP practices), with an extra part-time Social Prescriber in Networks 1 and 6

All of the Social Prescribers are required to undertake the following training as part of their contract:
· Motivational Interviewing, Making Every Contact Count (MECC), Information Governance, Basic Life Support, Safeguarding
Social Prescribers are line managed by the Network Manager in their GP Network. The multi-agency strategic Steering Group oversees service delivery, providing expertise and project management, driving and overseeing service developments, including around data recording, helping to rectify any operational issues and supporting service evaluation</td></tr>
<tr><td>How</td><td>Where Social Prescribers have access to EMIS19, referrals generally come via EMIS, otherwise hard copy referral forms are used, self-referrals are also accepted and can be made over the phone, via email, or via reception staff

- Client consultations are conducted either on the telephone or in face-to-face appointments, depending on need

- Social Prescribers use a range of motivational interviewing, goal-setting and coaching skills in their discussions with clients</td></tr>
<tr><td>Where</td><td>Face-to-face or via telephone, in three Networks, where felt necessary on an individual client basis

- Social Prescribers offer home visits</td></tr>
<tr><td>When and How Much</td><td>Each consultation: 30-60 min

- most Networks place no restriction on the number of consultations a client may have with a Social Prescriber, but two apply a guide to the maximum number, ranging from 4 to 6

- in five of the Networks, in exceptional circumstances, Social Prescribers accompany clients to services</td></tr>
<tr><td>Tailoring</td><td>Referral process differs slightly across the different GP Networks</td></tr>
</table>

Intervention: Light</td>
<td>Patient outcomes at baseline (pre intervention via Social Prescriber) and 12-weeks follow-up (via follow-up calls):

- Measure Yourself Concerns and Wellbeing (MyCaW)

Process of care measures 6 months before and 6 months after (data from case management system on referrals by practice and professional):

- - number of GP appointments</td>
<td>Peer-Review: no

Funding source:

Tower Hamlets Clinical Commissioning Group

Declaration of interest:

Not mentioned</td>
</tr>
</table>

<table>
<tr>
<td>Friedli 2012 (63)</td>
<td>Uncontrolled before-and-after study, mixed methods

Study length:

16 months</td>
<td>Inclusion criteria:

- Poor mental wellbeing affected by their social circumstances e.g. socially isolated, recently bereaved, lone parents, low income

- Mild to moderate depression and anxiety

- Long term physical/mental conditions

- Frequent attenders in primary care

Exclusion criteria:

- People experiencing acute episodes of psychosis.

- People with issues of drug or alcohol misuse.

Baseline characteristics:
<table>
<tr><td colspan="2">N</td><td>123</td></tr>
<tr><td>Age</td><td>Median (Fully engaged)</td><td>44</td></tr>
<tr><td>Gender</td><td>Female % (Fully Engaged)</td><td>57.4</td></tr>
<tr><td>Ethnicity</td><td>-</td><td>-</td></tr>
<tr><td>Living arrangement</td><td>-</td><td>-</td></tr>
<tr><td>Employment status</td><td>-</td><td>-</td></tr>
<tr><td>other</td><td>-</td><td>-</td></tr>
</table>

Population: General</td>
<td>Primary Care

Country (UK, Dundee, Maryfield)</td>
<td>
<table>
<tr><td>Name</td><td>Equally well: Sources of Support</td></tr>
<tr><td>Why</td><td>Adressing the high economic, social health burden imposed by mental illness and the corresponding requirement to improve mental wellbeing.</td></tr>
<tr><td>What</td><td>Social prescribing scheme, referrals by GPs, initial consultation aimed at exploring the psychosocial needs of patients. Depending on patient need, link workers facilitate access to a range of local sources of support, activities and opportunities in the community. Appropriate referral, engagement, signposting and supported access.</td></tr>
<tr><td>Who provided</td><td>Link worker</td></tr>
<tr><td>How</td><td>Contacted patient to arrange an initial consultation after referral by GP</td></tr>
<tr><td>Where</td><td>Face-to-face or via telephone</td></tr>
<tr><td>When and How Much</td><td>Up to four consultations</td></tr>
<tr><td>Tailoring</td><td>Depending in complexity of social and support needs and levels of distress</td></tr>
</table>

Intervention: Medium</td>
<td>Patient outcomes at baseline and post intervention (unknown time-frame:

- Warwick Edinburgh Mental Well-being Scale (WEMBWS)
- Work Social Adjustment Scale (WSAS)</td>
<td>Peer-Review: No

Funding sources: Dundee Healthy Living Initiative (DHLI)

Declaration of interest: not mentioned</td>
</tr>
</table>

<table>
<tr>
<td>Foster 2020 (64,65)</td>
<td>Uncontrolled before-and-after study, mixed-methods

Study length: 32 months</td>
<td>Inclusion criteria:

18 years or older, there was no specific eligibility criteria in relation to loneliness, although the service did target specific population groups (called trigger groups) including young parents, individuals with health and/or mobility issues and people recently bereaved, retired or had children leaving home.

Exclusion criteria: -

Baseline characteristics:

<table>
<tr><td colspan="2">N</td><td>10643</td></tr>
<tr><td>Age</td><td>Mean (SD)</td><td>65.5 (19.3)</td></tr>
<tr><td>Gender</td><td>Female %</td><td>65.8</td></tr>
<tr><td>Ethnicity</td><td>White British %</td><td>70.2</td></tr>
<tr><td>Living arrangement</td><td>Living alone %</td><td>65.4</td></tr>
<tr><td>Employment status</td><td>-</td><td>-</td></tr>
<tr><td>Other</td><td>Health issues %</td><td>49.3</td></tr>
</table>

Population: General</td>
<td>Primary Care, Secondary Care

Country: UK</td>
<td>
<table>
<tr><td>Name</td><td>Social prescribing service</td></tr>
<tr><td>Why</td><td>Reduce people's loneliness</td></tr>
<tr><td>What</td><td>Delivering personalised support, developing a supportive relationship with service-users, assessing their needs and providing person-tailored care, focused on developing service-users' confidence so they felt able to socialise and to facilitate access to appropriate community activities and services (signposting) such as craft groups, adult learning and leisure facilities.
While link workers primarily delivered support, volunteers were also recruited to increase service capacity. For example, a link worker may undertake a service-user's initial assessment and then a volunteer accompanies them to a community activity.</td></tr>
<tr><td>Who provided</td><td>Skilled, paid link workers alongside volunteers</td></tr>
<tr><td>How</td><td>Face-to-face</td></tr>
<tr><td>Where</td><td>Across 37 different sites throughout the UK</td></tr>
<tr><td>When and How Much</td><td>Up to 12 weeks</td></tr>
<tr><td>Tailoring</td><td>Personalized</td></tr>
</table>

Intervention: Holistic</td>
<td>Patient outcomes at baseline, 12-week endpoint and 3-month follow-up via questionnaire:

- UCLA Loneliness Scale

Process of care measures:

- Economic evaluation*
- - SROI analysis*</td>
<td>Peer-Review: partly (1/2)

Funding source: Co-op and British Red Cross charity partnership

Declaration of interest: none declared</td>
</tr>
<tr>
<td>Grant 2000 (20)</td>
<td>RCT, parallel group

Study length: -</td>
<td>Inclusion criteria:

- aged 16 years or over with psychosocial problems who general practitioners thought might benefit from contact with the voluntary sector

Exclusion criteria:

- patients who are unable to complete questionnaires owing to language difficulties, illiteracy, or learning disability

Baseline characteristics:

<table>
<tr><td></td><td></td><td>I</td><td>C</td></tr>
<tr><td colspan="2">N</td><td>90</td><td>71</td></tr>
<tr><td>Age</td><td>Mean (SD)</td><td>40.8 (15.5)</td><td>71, 45.6 (16.8)</td></tr>
<tr><td>Gender</td><td>Female %)</td><td>72</td><td>79</td></tr>
<tr><td>Ethnicity</td><td>-</td><td>-</td><td>-</td></tr>
<tr><td>Living arrangement</td><td>-</td><td>-</td><td>-</td></tr>
<tr><td>Employment status</td><td>Marital status: married or cohabiting %</td><td>44</td><td>-</td></tr>
</table>

Population: General</td>
<td>Primary Care, General practice

Country: UK (Avon, West England)</td>
<td>
<table>
<tr><td>Name</td><td>Amalthea group (intervention)</td><td>General practitioner group
(control)</td></tr>
<tr><td>Why</td><td>Improved access to the numerous voluntary organisations with a potentially useful role in the management of psychosocial problems, to improve patients' quality of life and to decrease time spent by healthcare professionals dealing with psychosocial problems</td><td>Control group in a randomized controlled trial setting</td></tr>
<tr><td>What</td><td>Initial assessment within seven days of referral and follow-up on one or more occasions to provide support and to encourage attendance at recommended local and national voluntary organisations</td><td>Routine care</td></tr>
<tr><td>Who provided</td><td>Three project facilitators from different backgrounds were trained and supervised by the organisation</td><td>General practitioners</td></tr>
<tr><td>How</td><td>Patients were offered an initial assessment within seven days of referral and were followed up on one or more occasions to provide support and to encourage attendance at recommended local and national voluntary organisations</td><td>-</td></tr>
<tr><td>Where</td><td>-</td><td>-</td></tr>
<tr><td>When and How Much</td><td>Initial assessment and one or more follow-ups</td><td>-</td></tr>
<tr><td>Tailoring</td><td>-</td><td>-</td></tr>
<tr><td>Modifications</td><td>-</td><td>-</td></tr>
</table>

Intervention: Medium</td>
<td>Patient outcomes at baseline (via questionnaires in the practice) and 1- and 4-months follow-up (mailed questionnaires):

- hospital anxiety and depression scale (HADS)
- social support (DUKE-UNC functional social support questionnaire)
- facets of quality of life (COOP/WONCA functional health assessment charts)
- delighted-terrible faces scale

Process of care measures (collected during follow-up period):

- number of contacts with primary healthcare team
- cost of contacts with primary healthcare team (£)
- number of prescriptions
- cost of prescriptions (£)
- number of referrals
- number of mental health referrals
- cost of referrals (£)
- total cost of primary healthcare team contacts, prescribing, and referrals (£)
- cost of time of Amalthea</td>
<td>Peer-Review: yes

Funding source: Avon Health Authority

Declaration of interest: none declared</td>
</tr>
</table>

<table>
<tr><td></td><td></td><td></td><td></td><td></td><td>Project facilitator (£)
- - total cost (£)</td><td></td></tr>
<tr>
<td>Grayer 2008 (66)</td>
<td>Uncontrolled before-and-after study

Study length:

12 months</td>
<td>Inclusion criteria:

- 18 years old or over
- with a psychosocial problem (broad: included common mental health problems such as anxiety and depression, and social problems such as isolation, relationship, housing and financial difficulties which might impact negatively upon patients' psycho-logical wellbeing)

Exclusion criteria:

- a current episode of acute psychosis or in crisis, being housebound, people who are, or who should be, under the care of other services, e.g. community mental health team (CMHT) or social services, active suicidal ideation, assessed as follows:
– if the patient scored three or over on items 6, 9, 22, 24 or 34 or scored one or over on item 16 of the CORE – Outcome Measure questionnaire (CORE-OM), then the GPCMHW asked the patient about suicidal intent. If clear intent was present, then the patient would be informed by the GPCMHW that they would have to break confidentiality and inform the GP. If the risk posed was judged by the GPCMHW to be immediate, then the patient would be asked to wait while the GPCMHW discussed this with the GP

Baseline characteristics:

<table>
<tr><td colspan="2">N</td><td>108</td></tr>
<tr><td>Age</td><td>Mean (SD)</td><td>43.14 (14.56)</td></tr>
<tr><td>Gender</td><td>Female %</td><td>62</td></tr>
<tr><td>Ethnicity</td><td>White %</td><td>67</td></tr>
<tr><td>Living arrangement</td><td>-</td><td>-</td></tr>
<tr><td>Employment status</td><td>Employed %</td><td>26.6</td></tr>
<tr><td>Other</td><td>-</td><td>-</td></tr>
</table>

Population: General</td>
<td>Primary care, General practice

country:

UK (London Boroughs of Camden and Islington, Inner London)</td>
<td>
<table>
<tr><td>Name</td><td>-</td></tr>
<tr><td>Why</td><td>Patients with psychosocial problems may benefit from a variety of community, educational, recreational and voluntary sector resources, but GPs often under-refer to these through lack of knowledge and time</td></tr>
<tr><td>What</td><td>Supported the patient's attendance at recommended organisations, e.g. making contact with or accompanying the patient to their initial meeting with the organization (in collaboration with voluntary sector staff as appropriate), or discuss alternatives if initial plans had been unsuccessful</td></tr>
<tr><td>Who provided</td><td>GPCMHWs (graduate primary care mental health worker): two recent psychology graduates

- once in post the workers received, in-house training and ongoing supervision from two clinical psychologists</td></tr>
<tr><td>How</td><td>Mostly referral from GPs to GPCMHW: to provide a link between the local voluntary and community sectors and inner-city primary healthcare staff across a number of practices

- GPCMHW researched the potential resources available for patients either during the initial session or thereafter, through a combination of paper and electronic directories, telephone enquiries, peer guidance, and supervision from a locally based clinical psychologist</td></tr>
<tr><td>Where</td><td>- 13 practices volunteered and participated in the study over a one year period

- in one Borough a 'hub and spoke' model was adopted, whereby the GPCMHW was based at four practices (for half a day/week each), but accepted referrals from an additional two local surgeries

- in the second Borough the GPCMHW was based for a half-day a week or fortnight at each of seven GP practices</td></tr>
<tr><td>When and How Much</td><td>The modal number of patient appointments was 2 (range 1 – 3, with the exception of 4 patients, who were seen on 4 or more occasions).</td></tr>
<tr><td>Tailoring</td><td>In some practices it was decided that only GPs were able to refer, while at others all clinicians could refer, and at some practices nonclinical staff such as the practice</td></tr>
</table>
</td>
<td>Patient outcomes at baseline (pre intervention, during initial appointment the GPCMHW administered questionnairs) and 3-months follow-up (research assistant met with the patient to administer questionnaires, if the patient did not want a face-to-face follow up appointment or defaulted ,the questionnaires were posted):

- General Health Questionnaire-1 (GHQ-12)
- Clinical Outcomes in Routine Evaluation-Outcomes Measure (CORE-OM)
- Work and Social Adjustment Scale (WSAS)
- clinical satisfaction questionnaire (CSQ, likert scale 1-4)

Process of care measures (obtained from patient medical records for the three month period prior to the date of referral and for the three month period following the patient's first appointment with the GPCMHW):

- PHC consultations
- PHC consultations with a psychosocial aspect
- onwards mental health</td>
<td>Peer-Review:

Yes

Funding source:

Camden and Islington HealthAction Zone, Camden PCT and Islington PCT

Declaration of interest: non declared</td>
</tr>
</table>

<table>
<tr>
<td></td>
<td></td>
<td></td>
<td></td>
<td><table><tr><td></td><td>manager made indirect referrals by making suggestions to clinical staff</td></tr></table>
Intervention: Holistic</td>
<td>related referrals
- - psychotropic medication</td>
<td></td>
</tr>
<tr>
<td>Healthy Dialogues Ltd 2018 (18)</td>
<td>Uncontrolled before-and-after study, mixed-methods

Study length:

12 months</td>
<td>Inclusion criteria:

- frequent attendance to GP services
- social isolation
- mild/moderate mental health issues
- social needs
- recent hospital admissions

Exclusion criteria:-

Baseline characteristics:
<table>
<tr><td>N</td><td></td><td>316</td></tr>
<tr><td>Age</td><td>40-49 years %</td><td>15</td></tr>
<tr><td>Gender</td><td>Female %</td><td>71</td></tr>
<tr><td>Ethnicity</td><td>White %</td><td>55</td></tr>
<tr><td>Living arrangement</td><td>-</td><td>-</td></tr>
<tr><td>Employment status</td><td>-</td><td>-</td></tr>
<tr><td>Other</td><td>-</td><td>-</td></tr>
</table>
Population: General</td>
<td>Primary care

Country:

UK (London Borough of Merton, South West London)</td>
<td><table>
<tr><td>Name</td><td>East Merton Social Prescribing Pilot</td></tr>
<tr><td>Why</td><td>Free up GP professional time while connecting people to their community and community resources, promote self-help, social engagement and resilience to its population in East Merton, improve the health and wellbeing of patients by providing access to non-medical support
- reduce avoidable costs including A&E attendances and hospital admissions</td></tr>
<tr><td>What</td><td>Initial consultation appointment, needs assessment structured around the Wellbeing STAR, plan, referral or signposting to activities provided by the local voluntary and community sector, basic assistance with form filling, benefits eligibility checks or engagement with mental health services</td></tr>
<tr><td>Who provided</td><td>One Social Prescribing Coordinator (SPC) (worked at both practices for two days a week each)</td></tr>
<tr><td>How</td><td>Face-to-face</td></tr>
<tr><td>Where</td><td>GP practices</td></tr>
<tr><td>When and How Much</td><td>One-hour initial consultation, where needed the SPC offers a follow-up appointment at three-monthly intervals</td></tr>
<tr><td>Tailoring</td><td>Personalized</td></tr>
</table>Intervention: Holistic</td>
<td>Patient outcomes (at each Social Prescribing appointment, the SPC asks the patient to fill in):

- Wellbeing Star

Process of care measures (3/6 months before and 3/6 months after intervention):

- number of GP appointments
- - A&E attendance</td>
<td>Peer-Review:

No

Funding source: Merton Partnership, Merton CCG and Merton Council Public Health

Declaration of interest: not mentioned</td>
</tr>
<tr>
<td>Holt 2020 (67)</td>
<td>Uncontrolled before-and-after study, multi-level, repeated measures design

Study length:

23 months</td>
<td>Inclusion criteria: -

Exclusion criteria:-

Baseline characteristics:
<table>
<tr><td>N</td><td></td><td>66</td></tr>
<tr><td>Age</td><td>Mean</td><td>47</td></tr>
<tr><td>Gender</td><td>Female %</td><td>87.9</td></tr>
<tr><td>Ethnicity</td><td>-</td><td>-</td></tr>
<tr><td>Living arrangement</td><td>-</td><td>-</td></tr>
<tr><td>Employment status</td><td>-</td><td>-</td></tr>
<tr><td>Other</td><td>-</td><td>-</td></tr>
</table>
Population: Unclear</td>
<td>Setting unclear

Country:

UK (Bristol, South West England)</td>
<td><table>
<tr><td>Name</td><td>Arts-on-prescription</td></tr>
<tr><td>Why</td><td>Evaluate the extent to which dimensions of mood (hedonic tone, tense arousal and energetic arousal) are affected by art-making, how these change over time, and whether this predicts any long-term changes in wellbeing

- test a novel evaluation tool (mood tracking) that is easy to deliver and useful for inferring mechanisms of change. to improve the mental health of patients and to reduce the burden on the National Health Service (NHS), social prescription is being explored as a route to reducing the financial burden of patientcare, decreasing visits to GPs</td></tr>
<tr><td>What</td><td>Range of art techniques and ideas to help patients explore their own creativity and were supported to learn and explore new artistic skills at their own pace throughout the programme

-activities are structured according to individual need and interest, and may include clay work, water colour, mixed media, drawing, print work, collage, felting, and so on</td></tr>
<tr><td>Who provided</td><td>Two skilled arts and health practitioner</td></tr>
<tr><td>How</td><td>Face-to-face</td></tr>
<tr><td>Where</td><td>-</td></tr>
<tr><td>When and How Much</td><td>12 weeks, typically weekly meetings for about 2 hours</td></tr>
<tr><td>Tailoring</td><td>-</td></tr>
</table>
Intervention: Medium</td>
<td>Patient outcomes at the start of the programme,

before a half-term break, on their return

from this break and at the end of the

programme. Participants were also

invited to complete the SMS at the start

and end of each art workshop via questionnaire:

- Short Mood Scale (SMS)*
- Warwick Edinburgh Mental Wellbeing Scale (WEMWBS)</td>
<td>Peer-Review:

Yes

Funding source: No financial support

Declaration of interest: None declared</td>
</tr>
</table>

<table>
<tr>
<td>Howarth 2020 (68)</td>
<td>Uncontrolled before-and-after study, mixed-methods

Study length:

-</td>
<td>Inclusion criteria: -

Exclusion criteria:-

Baseline characteristics:
<table>
<tr><td>N</td><td></td><td>47</td></tr>
<tr><td>Age</td><td>Range</td><td>30-85</td></tr>
<tr><td>Gender</td><td>Female %</td><td>-</td></tr>
<tr><td>Ethnicity</td><td>-</td><td>-</td></tr>
<tr><td>Living arrangement</td><td>-</td><td>-</td></tr>
<tr><td>Employment status</td><td>-</td><td>-</td></tr>
<tr><td>Other</td><td>-</td><td>-</td></tr>
</table>

Population: General</td>
<td>Primary Care

Country:

UK (Salford, Greater Manchester, England)</td>
<td>
<table>
<tr><td>Name</td><td>Bridgewater Therapeutic Garden</td></tr>
<tr><td>Why</td><td>Create a sanctuary that offers space to grow, space to reflect and space to meet others and get moving

-help people to heal while reversing the challenges and potential negative health and environmental impacts of the philosophical challenge of the nature deficit disorder, to provide a place of belonging in order to reverse the challenges of social isolation</td></tr>
<tr><td>What</td><td>Community Nurses in the locality refer patients to the link worker and help promote this asset based, salutogenic approach to wellbeing.</td></tr>
<tr><td>Who provided</td><td>Link worker</td></tr>
<tr><td>How</td><td>Face-to-face</td></tr>
<tr><td>Where</td><td>-</td></tr>
<tr><td>When and How Much</td><td>12 weeks</td></tr>
<tr><td>Tailoring</td><td>-</td></tr>
</table>

Intervention: Medium</td>
<td>Patient outcomes at baseline and unspecified endpoint (unclear timepoint):

- Short Warwick Edinburgh Mental Wellbeing Scale (SWEMWBS)</td>
<td>Peer-Review:

Yes

Funding source: -

Declaration of interest: None declared</td>
</tr>
<tr>
<td>Jones 2018 (a) (69)</td>
<td>Uncontrolled before-and-after study, mixed-methods

Study length:

17 months</td>
<td>Inclusion criteria: -

Exclusion criteria: -

Baseline characteristics:
<table>
<tr><td>N</td><td></td><td>78</td></tr>
<tr><td>Age</td><td>-</td><td>-</td></tr>
<tr><td>Gender</td><td>-</td><td>-</td></tr>
<tr><td>Ethnicity</td><td>-</td><td>-</td></tr>
<tr><td>Living arrangement</td><td>-</td><td>-</td></tr>
<tr><td>Employment status</td><td>-</td><td>-</td></tr>
<tr><td>Other</td><td>-</td><td>-</td></tr>
</table>

Population: Unclear</td>
<td>Primary care

UK (South West Cardiff, City and County of Cardiff, Wales)</td>
<td>
<table>
<tr><td>Name</td><td>SPICE Time Credit Social Prescribing Model</td></tr>
<tr><td>Why</td><td>The Welsh health and care system is struggling to balance increased demands with reduced expenditure

- around 20% of patients consult their GP for psychosocial problems, it has been argued that psychosocial issues and long-term conditions can be better managed in the community

-s.p. can offer an alternative to the traditional medical models and reduce the burden on the NHS

- increased levels of community and social participation has a positive impact on health behaviours, physical and emotional health and self-confidence, especially among disadvantaged populations</td></tr>
<tr><td>What</td><td>‘Social Prescribing Time Credit Model´:

- patients and community members are ‘prescribed’ a small number of TC by the social prescriber(s) based in the GP practice(s) pledging their time to earn in the future, this enables them to access a wide range of activities that they are interested in, spending time credits, and to identify a way they can play a positive role in the community through earning by volunteering

- this supports the voluntary sector to work with increased numbers of patients signposted via social prescribing</td></tr>
<tr><td>Who provided</td><td>2 social prescriber</td></tr>
<tr><td>How</td><td>Face-to-face</td></tr>
<tr><td>Where</td><td>-</td></tr>
<tr><td>When and How Much</td><td>-</td></tr>
<tr><td>Tailoring</td><td>Personalized</td></tr>
</table></td>
<td>Patient outcomes (pre and post intervention collected by the social prescribers anonymised with coded unique patient numbers):

- Warwick Edinburgh, Mental Wellbeing Scale* (WEMWBS)

Process of care measures (12 months prior to intervention and at the end of the pilot from the GP Vision system (anonymised with unique patient numbers)):

- GP appointments per person per year (extrapolated) Change*
- current condition*
- medication use*
- - unplanned hospital admission *</td>
<td>Peer-Review: no

Funding source:

Welsh Government, I2S fund

SPICE: social enterprise founded

Declaration of interest:

Not mentioned</td>
</tr>
</table>

<table>
<tr><td></td><td></td><td></td><td></td><td>Intervention: Light</td><td></td><td></td></tr>
<tr>
<td>Jones 2018 (b) (70)</td>
<td>Uncontrolled before-and-after study, mixed-methods

Study length:

7 months</td>
<td>Inclusion criteria:

- patients with low level anxiety and depression and psychosocial issues

Exclusion criteria: -

Baseline characteristics:
<table>
<tr><td colspan="2">N</td><td>31</td></tr>
<tr><td>Age</td><td>> 45 years %</td><td>56,3</td></tr>
<tr><td>Gender</td><td>-</td><td>-</td></tr>
<tr><td>Ethnicity</td><td>-</td><td>-</td></tr>
<tr><td>Living arrangement</td><td>-</td><td>-</td></tr>
<tr><td>Employment status</td><td>-</td><td>-</td></tr>
<tr><td>Other</td><td>-</td><td>-</td></tr>
</table>
Population: Special (mental health issues)</td>
<td>Primary care

UK</td>
<td><table>
<tr><td>Name</td><td>Grow Cardiff Gardening Social Prescribing pilot</td></tr>
<tr><td>Why</td><td>Regular contact with nature can promote positive health behaviours and a regular contact with nature is a form of preventive medicine

- around 20% of patients consult their GP for psychosocial problems, it has been argued that

psychosocial issues and long-term conditions can be better managed in the community</td></tr>
<tr><td>What</td><td>6-months SP pilot in a local community garden or a garden attached to the surgery

- patients were supervised by a gardener and attended regular gardening sessions once a week</td></tr>
<tr><td>Who provided</td><td>Directly through gardener</td></tr>
<tr><td>How</td><td>patients identified by GP's and practice staff in 3 surgeries in the SWC GP Cluster were referred directly</td></tr>
<tr><td>Where</td><td>Local community garden</td></tr>
<tr><td>When and How Much</td><td>Once a week, delivered over a 5-months period</td></tr>
<tr><td>Tailoring</td><td>Personalized</td></tr>
</table>
Intervention: Light</td>
<td>Patient outcomes pre and post intervention (collected by gardeners):

- Warwick Edinburgh, Mental Wellbeing Scale* (WEMWBS)
- UCLA Loneliness Scale*
- Self-Efficacy for Management of Chronic Disease scale*

Self-reported levels of:

- Levels of Physical Activity*
- Number of Fruit and Vegetables Consumed*
- sleep*

Process of care measures in previous 12 months and over the 5 months of the intervention:

- GP appointments*
- prescription dispensed*
- - cost analysis pre-intervention and cost savings over the 5 months of intervention*</td>
<td>Peer-Review: no

Funding source:

Innovate to Save Pilot (I2S)

Declaration of interest: not mentioned</td>
</tr>
<tr>
<td>Kellezi 2019 (71,72)</td>
<td>Uncontrolled before-and-after study, mixed-methods</td>
<td>Inclusion criteria:

Over 18 years, live in the relevant area in Nottingham (or are registered with a GP in the area), are managing one or more long-term health conditions and feel isolated, lonely or anxious (from protocol)

Exclusion criteria: -</td>
<td>Primary Care

Country:

UK (East Midlands)</td>
<td><table>
<tr><td>Name</td><td>Social prescribing pathway</td></tr>
<tr><td>Why</td><td>Reduce the economic burden of loneliness, increase patients' illness self-management, address their psychosocial and health needs, and through this to reduce primary healthcare usage</td></tr>
<tr><td>What</td><td>Referred by GPs during consultations, by third-sector organisations operating within Nottingham and by self-referral after seeing promotional materials in GP surgeries,</td></tr>
</table></td>
<td>Patient outcomes at baseline, 4 and 6 months endpoint:

- EQ-5D
- UCLA Loneliness Scale
- Community belonging
- Number of group memberships</td>
<td>Peer-Review:

Yes

Funding source:</td>
</tr>
</table>

<table>
<tr>
<td></td>
<td>Study length:

16 months</td>
<td>Baseline characteristics:
<table><tr><td>N</td><td></td><td>630</td></tr><tr><td>Age</td><td>Mean (SD)</td><td>52.74 (14.79)</td></tr><tr><td>Gender</td><td>Female %</td><td>54</td></tr><tr><td>Ethnicity</td><td>-</td><td>-</td></tr><tr><td>Living arrangement</td><td>-</td><td>-</td></tr><tr><td>Employment status</td><td>-</td><td>-</td></tr><tr><td>Other</td><td>Relationship %</td><td>59.2</td></tr></table>
Population: General</td>
<td></td>
<td><table><tr><td></td><td>libraries and community centres. Once recruited onto the pathway, patients have an initial meeting and needs assessment with an HC, who either prescribes self-care management or refers to an LW, who in turn connects the patient with relevant third-sector groups. HC/LWs regularly check on patients' progress.</td></tr><tr><td>Who provided</td><td>Health coaches (HC) and link workers (LW)</td></tr><tr><td>How</td><td>Face-to-face</td></tr><tr><td>Where</td><td>GP surgeries</td></tr><tr><td>When and How Much</td><td>The aim of the pathway was to support each patient weekly for up to 8 weeks. The length of support depended on the specific paths offered. The initial appointment lasted over 1 hour, and further appointments ranged in length based on the activities in which the patients were involved.</td></tr><tr><td>Tailoring</td><td>Personalized</td></tr></table>
Intervention: Holistic</td>
<td>- Social support

Process of care measures at baseline and 4 months endpoint:

- Primary care use
- Primary care services use</td>
<td>ImROC (Implementing Recovery Through Organisational Change). The study was sponsored by Nottingham Trent University

Declarations of interest:

None declared</td>
</tr>
<tr>
<td>Kensington & Chelsea Social Council 2018 (73)</td>
<td>Uncontrolled before-and-after study, mixed-methods

Study length:

11 months</td>
<td>Inclusion criteria:

Tier 0:+65 years of age and are mostly healthy.

Tier 1:+65 years of age and have one well-managed Long-Term Condition (LTC).

Tier 2:+65 years of age and have two LTCs, mental health or social care needs.

Tier 3:+65 years of age and have three or more LTCs, mental health or social care needs

In the beginning Tier 2+3, expanded to Tier 1

Exclusion criteria: -

Baseline characteristics:
<table><tr><td>N</td><td></td><td>807</td></tr><tr><td>Age</td><td>-</td><td>-</td></tr><tr><td>Gender</td><td>-</td><td>-</td></tr><tr><td>Ethnicity</td><td>-</td><td>-</td></tr><tr><td>Living arrangement</td><td>-</td><td>-</td></tr><tr><td>Employment status</td><td>-</td><td>-</td></tr><tr><td>Other</td><td>Tier 2/3 %</td><td>52.8/34.6</td></tr></table>
Population: Special (Geriatric age group)</td>
<td>Primary Care

Country:

UK (Royal Borough of Kensington and Chelsea, West Central London)</td>
<td><table><tr><td>Name</td><td>Self-Care Social Prescribing</td></tr><tr><td>Why</td><td>Increase patient confidence in making informed decisions about their health. It is expected to positively contribute to patients' confidence and motivation, which in turn is expected to contribute towards a long-term reduction in use of primary, secondary, and some tertiary services.</td></tr><tr><td>What</td><td>Social prescribing model links patients in primary care with sources of health and wellbeing support from specialist voluntary and community services,

Step 1: Patient is allocated to a practice-based Health and Social Care Assistant (HSCA) or Case Manager (CM),

Step 2: Patient assessment, completion of referral form with patient's requirements and notes on their situation - Direct referral to an appropriate service from the Self-Care directory of services,

Step 3: Referral completion.</td></tr><tr><td>Who provided</td><td>Practice-based Health and Social Care Assistant (HSCA) or Case Manager (CM)</td></tr><tr><td>How</td><td>-</td></tr><tr><td>Where</td><td>GP surgeries</td></tr><tr><td>When and How Much</td><td>Six service sessions after initial patient assessment</td></tr><tr><td>Tailoring</td><td>Personalized</td></tr></table>
Intervention: Medium</td>
<td>Patient outcomes at baseline and unclear endpoint:

- EQ-5D
- Warwick Edinburgh Mental Wellbeing Scale
- Patient Health Questionnaire (PHQ-9)
- Investigating Choice Experiments for the Preferences of Older People Capability Measure for Adults
- The Short Form (12) Health Survey

Process of care measures:

- Economic evaluation
- Social Return on Investment</td>
<td>Peer-Review:

No

Funding source:

Kensington & Chelsea Social Council and NHS West London Clinical Commissioning Group

Declarations of interest:

Unknown</td>
</tr>
</table>

<table>
<tr>
<td>Kimberlee 2014 (74,75)</td>
<td>Uncontrolled before-and-after study, mixed-methods

Study length:

12 months</td>
<td>Inclusion criteria: -

Exclusion criteria: -

Baseline characteristics:
<table>
<tr><td>N</td><td></td><td>128</td></tr>
<tr><td>Age</td><td>Median age</td><td>36-40</td></tr>
<tr><td>Gender</td><td>Female %</td><td>48.8</td></tr>
<tr><td>Ethnicity</td><td>White %</td><td>83</td></tr>
<tr><td>Living arrangement</td><td>Living Alone %</td><td>37.9</td></tr>
<tr><td>Employment status</td><td>Unemployed %</td><td>40</td></tr>
<tr><td>Other</td><td>-</td><td>-</td></tr>
</table>

Population: Unclear</td>
<td>Primary Care

Country:

UK (Bristol, South West England)</td>
<td>
<table>
<tr><td>Name</td><td>Wellspring Healthy Living Centre's (WHLC) Wellbeing Programme (WP)</td></tr>
<tr><td>Why</td><td>Patients presenting with complex mental health needs challenge traditional delivery in general practice, relocate services closer to individuals and the communities where they live.</td></tr>
<tr><td>What</td><td>Branching Out -- 1:1 sessions based on the support-worker model and best practice from the Deep Value project of Community Links in East London i.e. the development of a strong, trusting and functioning relationship with a support health worker. Service-users are supported to identify and achieve realistic goals that will improve their self-esteem and expand their life choices, including attending peer-support groups and activities, volunteering, accessing training and employment, and by referring and supporting service-users to counselling, debt advice, parent & family support and housing.

Time-Out Groups -- a weekly session whereby local residents engage in an activity with the aim of improving self-confidence and resilience, forming friendships for mutual support and to reduce isolation. Service-users are then supported to form their own groups around a shared interest such as cooking, physical activity or crafts/art activity. This aims to lessen reliance on workers and helps build resilience, personal responsibility and social wealth back into the community.</td></tr>
<tr><td>Who provided</td><td>Male and a female support worker</td></tr>
<tr><td>How</td><td>1:1 sessions, group sessions</td></tr>
<tr><td>Where</td><td>-</td></tr>
<tr><td>When and How Much</td><td>Over 9 weeks, weekly group get-togethers</td></tr>
<tr><td>Tailoring</td><td>Personalized</td></tr>
</table>

Intervention: Holistic</td>
<td>Patient outcomes at baseline and at least 3 months later:

- Patient Health Questionnaire (PHQ-9)
- Generalised anxiety disorder-7 scale
- Friendship scale
- Office for National Statistics Wellbeing Scale
- International Physical Activity Questionnaire
- Wellspring Wellbeing Questionnaire

Process of care measures 12 months before and after BO enrolment, subgroups analysed:

- Number of GP telephone contacts
- Number of GP face-to-face contacts
- Employment

Economic evaluation:

- Social Return on Investment</td>
<td>Peer-Review:

Partly (1/2)

Funding source:

Predominantly by the Henry Smith and Tudor Trust

Declarations of interest:

Unknown</td>
</tr>
<tr>
<td>Kimberlee 2016 (76)</td>
<td>Uncontrolled before-and-after study, mixed-methods

Study length:

-</td>
<td>Inclusion criteria:

Initially social prescribing was aimed at patient groups who were perceived as frequent attenders in primary care. These were vulnerable and at risk groups and people with long-term conditions.

Exclusion criteria: -

Baseline characteristics:
<table>
<tr><td>N</td><td></td><td>2047</td></tr>
<tr><td>Age</td><td>Mean (SD)</td><td></td></tr>
<tr><td>Gender</td><td>Female %</td><td>60.2</td></tr>
<tr><td>Ethnicity</td><td>White %</td><td>98.8</td></tr>
<tr><td>Living arrangement</td><td>-</td><td>-</td></tr>
<tr><td>Employment status</td><td>-</td><td>-</td></tr>
<tr><td>Other</td><td>Referral reason mental health and well being %</td><td>48</td></tr>
</table>

Population: General</td>
<td>Primary Care

Country:

UK (Gloucestershire, South West England)</td>
<td>
<table>
<tr><td>Name</td><td>Gloucestershire social prescribing</td></tr>
<tr><td>Why</td><td>Ensure individuals are able to make informed choices to manage their self-care and wellbeing needs, advise individuals how to access support networks, support and enable positive risk management and risk taking to maximize independence and choice, reduce use of statutory services, where appropriate.</td></tr>
<tr><td>What</td><td>A hub coordination model was advised with coordinators based in GP Practices. However, the intention was that the model going forward should be a mixture of formal referral and signposting. This would involve formal referral to larger third sector partners with paid staff who could then potentially act as caseworkers and informal signposting to a range of smaller, community level organisations and groups.</td></tr>
<tr><td>Who provided</td><td>Hub coordinator</td></tr>
<tr><td>How</td><td>Face-to-face</td></tr>
<tr><td>Where</td><td>GP surgeries</td></tr>
<tr><td>When and How Much</td><td>Coordinators' time with a patient once referred also varies.</td></tr>
<tr><td>Tailoring</td><td>Personalized</td></tr>
</table>

Intervention: Medium</td>
<td>Patient outcomes:

- Warwick Edinburgh Mental Wellbeing Scale
- Patient Health Questionnaire (PHQ-9)

Process of care measures:

- A&E admissions
- A&E attendance
- GP visits
- Home visits
- Telephone calls

Six months before and after referral.

- Economic evaluation
- Social Return on Investment</td>
<td>Peer-Review:

No

Funding source:

Gloucestershire Clinical Commissioning Group, Prime Minister's Challenge Fund

Declarations of interest:

Unknown</td>
</tr>
<tr>
<td>Liles 2017 (77)</td>
<td>Uncontrolled before-and-after study, mixed methods</td>
<td>Inclusion criteria: -

Exclusion criteria: -

Baseline characteristics:</td>
<td>Primary Care

Country:</td>
<td>
<table>
<tr><td>Name</td><td>Social Prescribing in Wessex, 8 unique projects</td></tr>
<tr><td>Why</td><td>Understand and address people's needs in a holistic way and to support them to</td></tr>
</table></td>
<td>Patient outcomes at Baseline and after referral:

- R-Outcomes (howRu)</td>
<td>Peer-Review:

No</td>
</tr>
</table>

<table>
<tr>
<td></td>
<td>Study length:

-</td>
<td>
<table>
<tr><td>N</td><td></td><td>-</td></tr>
<tr><td>Age</td><td>-</td><td>-</td></tr>
<tr><td>Gender</td><td>-</td><td>-</td></tr>
<tr><td>Ethnicity</td><td>-</td><td>-</td></tr>
<tr><td>Living arrangement</td><td>-</td><td>-</td></tr>
<tr><td>Employment status</td><td>-</td><td>-</td></tr>
<tr><td>Other</td><td>-</td><td>-</td></tr>
</table>

Population: Unclear
</td>
<td>UK (Wessex, South England)</td>
<td>
<table>
<tr><td></td><td>take greater control of their own life, improve their health and wellbeing</td></tr>
<tr><td>What</td><td>1. Older vulnerable patients, with whom they develop a holistic plan covering care coordination and connection with the local voluntary sector, Half of the patients are referred by the primary care team and half come from telephoning all older people discharged from hospital, provide a 7-day service, with one CN being on duty at the weekend,

2. people are referred by primary care teams and other statutory and public services, provide information and coordinate care to reduce social isolation and improve planned uptake of services,

3. focus of providing signposting and support for people aged over 70, 60% of referrals come from GPs and the remainder from a wide range of sources in primary care and other local services,

4. provide a blend of holistic care planning and social prescribing for people aged over 70 identified as being at risk of deterioration, holistic assessment that will involve social prescribing and referral to health and care services,

5. Most patients are referred by their GP (65%) or the locality Integrated Care Team (25%), guided conversations, complete wellbeing assessments and develop personalised support plans,

6. recruited by members of their local community. Introductions (not referrals) can come from anywhere, professionals, community members or individuals, Support is based upon a conversation about what a ‘Good Life’ would like, focusing on a person’s gifts, skills, interest and experiences,

7. provide a blend of holistic care planning, medicines review and social prescribing for older people, undertake the holistic assessments; supported by a team of Assistant Practitioners who support delivery of the plan with patients and carers, majority of referrals come from GP’s and most patients will be visited twice,

8. volunteer delivered, Signposters are attached to a local General Practice and two-thirds of referrals come from primary care with the remainder being self referrals or from a wide range of other local organisations, service is aimed at people struggling to manage a long term condition, who need support following a diagnosis, are socially isolated or need help with housing and benefits</td></tr>
<tr><td>Who provided</td><td>1. GP Federation, 5 Care Navigators, with one attached to each GP,

2. Care Navigators,

3. Care Navigators,

4. Proactive Care Nurses and Care Coordinators,

5. Coordinators,

6. Coordinators,

7. nurse specialist, community pharmacist,

8. trained volunteers</td></tr>
<tr><td>How</td><td>Telephone</td></tr>
<tr><td>Where</td><td>3+4 in GP</td></tr>
<tr><td>When and How Much</td><td>1. Patients are supported for as long as they need it, which on average is for 6.5 weeks,

2. up to 6 visits,

3. typically over a 2-4 week period,

4. initial 40 minute home visit, typically two home visits,

5. no time limit for supporting</td></tr>
<tr><td>Tailoring</td><td>Personalized</td></tr>
</table>
</td>
<td>Process of care measures:

- Economic evaluation

Staff outcome:

- R-Outcomes</td>
<td>Funding source:

-

Declarations of interest:

Unknown</td>
</tr>
</table>

<table>
<tr><td></td><td></td><td></td><td></td><td>Intervention: Light/Medium/Holistic</td><td></td><td></td></tr>
<tr>
<td>Loftus 2017 (19)</td>
<td>Uncontrolled before-and-after study

Study length:

9.1 months</td>
<td>Inclusion criteria:

Over 65 years of age with a chronic condition (including falls, social isolation, depression/anxiety) and either evidence of polypharmacy (defined as five or more repeat medications) or viewed by the GP as a frequent GP attender

Exclusion criteria: -

Baseline characteristics:
<table>
<tr><td>N</td><td></td><td>68</td></tr>
<tr><td>Age</td><td>Mean (SD)</td><td>72.9 (7.3)</td></tr>
<tr><td>Gender</td><td>Female %</td><td>70.6</td></tr>
<tr><td>Ethnicity</td><td>-</td><td>-</td></tr>
<tr><td>Living arrangement</td><td>-</td><td>-</td></tr>
<tr><td>Employment status</td><td>-</td><td>-</td></tr>
<tr><td>Other</td><td>Diagnosis anxiety or depression %</td><td>26.5</td></tr>
</table>

Population: Special (geriatric age group)</td>
<td>Primary Care

Country:

UK (Derry, Northern Ireland)</td>
<td><table>
<tr><td>Name</td><td>-</td></tr>
<tr><td>Why</td><td>Provide health and wellbeing, emotional and practical support, education and self-help groups</td></tr>
<tr><td>What</td><td>Referrals came from local GPs in one practice, program included social clubs, Men's Shed, counselling, arts program, falls prevention, exercises classes, crochet classes, personal development, craft classes, befriending and computer courses.</td></tr>
<tr><td>Who provided</td><td>Social prescribing coordinator</td></tr>
<tr><td>How</td><td>Telephone</td></tr>
<tr><td>Where</td><td>-</td></tr>
<tr><td>When and How Much</td><td>12 weeks</td></tr>
<tr><td>Tailoring</td><td>Personalized</td></tr>
</table>

Intervention: Holistic</td>
<td>Process of care measures 3 Months before and 3 months during or after referral, intention-to-treat and per-protocol analysis:

- Number of patients > 1 GP surgery visits
- Number of patients > 1 GP home visits
- Number of patients > 1GP telephone calls
- Number of patients with 0 or 1 GP contact
- Number of patients with no new repeat medications</td>
<td>Peer-Review:

Yes

Funding source:

Western Health and Social Care Trust

Declarations of interest:

None declared</td>
</tr>
<tr>
<td>Longwill 2014 (78)</td>
<td>Uncontrolled before-and-after study, mixed methods

Study length:

-</td>
<td>Inclusion criteria: -

Exclusion criteria: -

Baseline characteristics:
<table>
<tr><td>N</td><td></td><td>1089</td></tr>
<tr><td>Age</td><td>Mode age group</td><td>41-55</td></tr>
<tr><td>Gender</td><td>Female %</td><td></td></tr>
<tr><td>Ethnicity</td><td>-</td><td>-</td></tr>
<tr><td>Living arrangement</td><td>Living Alone %</td><td></td></tr>
<tr><td>Employment status</td><td>Unemployed %</td><td>49</td></tr>
<tr><td>Other</td><td>-</td><td>-</td></tr>
</table>

Population: Unclear</td>
<td>Primary Care

Country:

UK (London Borough of Hackney, East London</td>
<td><table>
<tr><td>Name</td><td>Hackney WellFamily service</td></tr>
<tr><td>Why</td><td>To promote health and social wellbeing, improve whole family wellbeing by reducing stress, improving health, providing support to individuals to end abusive relationships and improving family relationships</td></tr>
<tr><td>What</td><td>GPs can refer to the service easily through written referrals or using the EMIS clinical information system. Provides recovery-focused and holistic interventions include a mix of individually targeted and flexible practical and emotional support:

1. Advice and information (e.g. regarding housing, debt, welfare benefits, employment support

2) Counselling for emotional problems including anxiety, depression, bereavement and relationship difficulties

3) Promotion of leisure, social and physical activities and volunteering opportunities

4) Signposting and referral to other services

5) Carer support and peer support</td></tr>
<tr><td>Who provided</td><td>Team of 5.4 w.t.e workers and 2.00 w.t.e senior practitioners.</td></tr>
<tr><td>How</td><td>Face-to-face</td></tr>
<tr><td>Where</td><td>Familiar, accessible and non-stigmatised setting</td></tr>
<tr><td>When and How Much</td><td>Flexible over 6-12 weeks with 6-8 sessions but duration is linked to assessed level of need.</td></tr>
<tr><td>Tailoring</td><td>-</td></tr>
</table></td>
<td>Patient outcomes at Baseline and post-intervention:

- Generalised anxiety disorder-7 scale
- Patient Health Questionnaire (PHQ-9)
- Clinical Outcomes in Routine Evaluation system (CORE10)
- Recovery Star

Satisfaction with service at follow-up via questionnaire.</td>
<td>Peer-Review:

No

Funding source:

Primary Care Trust

Declarations of interest:

Unknown</td>
</tr>
</table>

<table>
<tr><td></td><td></td><td></td><td></td><td>Intervention: Holistic</td><td></td><td></td></tr>
<tr>
<td>Massie 2019 (79)</td>
<td>Uncontrolled before-and-after study, mixed methods

Study length:

20.5 months</td>
<td>Inclusion criteria: -

Exclusion criteria: -

Baseline characteristics:
<table>
<tr><td>N</td><td></td><td>676</td></tr>
<tr><td>Age</td><td>Mean (SD)</td><td>66.4 (18.6)</td></tr>
<tr><td>Gender</td><td>Female %</td><td>64</td></tr>
<tr><td>Ethnicity</td><td>White British %</td><td>68</td></tr>
<tr><td>Living arrangement</td><td>-</td><td>-</td></tr>
<tr><td>Employment status</td><td>Retired %</td><td>67</td></tr>
<tr><td>Other</td><td>Referral reason reduction in social isolation and loneliness %</td><td>35</td></tr>
</table>
Population: Unclear</td>
<td>Primary Care

Country:

UK (Wolverhampton, West Midlands, Western-central England)</td>
<td><table>
<tr><td>Name</td><td>Wolverhampton Social Prescribing Service</td></tr>
<tr><td>Why</td><td>The service aims to help people with non-clinical needs access a wide variety of services and activities provided by voluntary and statutory organisations and community groups.</td></tr>
<tr><td>What</td><td>Upon receiving a referral, link workers make contact with the patient within three days, to introduce themselves and the service, and arrange a face-to-face meeting within 10 days. From these conversations the link worker will identify the issues and co-design a solution with the service user. This may include the link worker accompanying an individual to the first session of a group activity and follow up phone calls to check on progress. The link worker makes a judgement as to when the service user is ready to exit the social prescribing service – this can vary dependent upon the support required, and in some instances are open for longer due to complex lives and multiple issues.</td></tr>
<tr><td>Who provided</td><td>Three link workers and a 0.5 WTE Project Manager to oversee the implementation of the pilot supported by a 0.5 WTE Administrator.</td></tr>
<tr><td>How</td><td>Face-to-face</td></tr>
<tr><td>Where</td><td>-</td></tr>
<tr><td>When and How Much</td><td>The link worker makes a judgement as to when the service user is ready to exit the social prescribing service – this can vary dependent upon the support required.</td></tr>
<tr><td>Tailoring</td><td>Personalized</td></tr>
</table>
Intervention: Holistic</td>
<td>Patient outcomes at baseline and after 1-7 months:
- Office for National Statistics Wellbeing Scale
- De Jong Gierveld Loneliness scale

Process of care measures 6 months before and after referral to social prescribing:
- GP visits
- Nurse visits
- Primary care
- A&E attendance
- Urgent care visits
- Emergency admissions
- Hospital bed days
- Secondary care

Economic evaluation:
- Social Return on Investment</td>
<td>Peer-Review:

No

Funding source:

Wolverhampton Clinical Commissioning Group (CCG)

Declarations of interest:

Unknown</td>
</tr>
<tr>
<td>Maund 2019 (80)</td>
<td>Uncontrolled before-and-after study, mixed methods

Study length:

-</td>
<td>Inclusion criteria:

1) Aged 18 years or older;

2) be registered with the community mental wellbeing service;

3) diagnosed with depression and/or anxiety (as categorised by mental health support workers);

4) physically able to take part in the range of outdoor activities; and

5) deemed able to provide informed consent

Exclusion criteria: -

Baseline characteristics:
<table>
<tr><td>N</td><td></td><td>18</td></tr>
<tr><td>Age</td><td>> 50 years %</td><td>50</td></tr>
<tr><td>Gender</td><td>Female %</td><td>50</td></tr>
<tr><td>Ethnicity</td><td>White British %</td><td>81</td></tr>
<tr><td>Living arrangement</td><td>-</td><td>-</td></tr>
<tr><td>Employment status</td><td>Unemployed %</td><td>63</td></tr>
<tr><td>Other</td><td>-</td><td>-</td></tr>
</table>
Population: Special (mental health issues)</td>
<td>Secondary Care

Country:

UK (Gloucestershire, South West England)</td>
<td><table>
<tr><td>Name</td><td>Wetlands for Wellbeing</td></tr>
<tr><td>Why</td><td>To improve the mental health of individuals experiencing anxiety and/or depression through engagement with nature.</td></tr>
<tr><td>What</td><td>Participants were recruited through a community mental wellbeing service. This non-governmental organisation offers

one-to-one and group therapy, as well as signposting individuals to suitable health promoting

interventions delivered by third-party organisations. A structured six-week nature-based health intervention (NBI) consisted of a broad range of nature-focused activities that took place within a wetland site.</td></tr>
<tr><td>Who provided</td><td>Community mental wellbeing service: mental health support workers</td></tr>
<tr><td>How</td><td>Face-to-face</td></tr>
<tr><td>Where</td><td>Wildfowl & Wetlands Trust (WWT) wetland site in Gloucestershire, UK</td></tr>
<tr><td>When and How Much</td><td>Participants took part in a two-hour session per week for six consecutive weeks.</td></tr>
<tr><td>Tailoring</td><td>-</td></tr>
</table>
Intervention: Medium</td>
<td>Patient outcome at baseline and after 6 weeks:
- Warwick Edinburgh Mental Wellbeing Scale
- Generalised anxiety disorder-7 scale
- Perceived Stress Scale
- Positive and Negative Affect Schedule</td>
<td>Peer-Review:

Yes

Funding source:

Horizon 2020 (Consolidator Grant No. 726104), Scottish Government's Rural and Environment Sciences and Analytical Services Division (RESAS)

Declarations of interest:

None declared</td>
</tr>
<tr>
<td>Mercer 2019 (92,93)</td>
<td>Cluster randomized</td>
<td>Inclusion criteria:

Intervention:</td>
<td>Primary Care</td>
<td></td>
<td>Patient outcomes at baseline and 9 months follow-up via</td>
<td>Peer-Review:

Partly (1/2)</td>
</tr>
</table>

<table>
<tr>
<td></td>
<td>controlled trial, quasi-experimental, mixed-methods

Study length:

-</td>
<td>Adult patients (aged 18 years or older).

Control:

- 1,000 adult patients (aged 18 years or older) registered with a comparator practice were randomly selected for invitation to participate in the evaluation.

- Practices were eligible for the program if they were located in Glasgow and in the 100 practices in Scotland serving the most-deprived patients (15% most-deprived postcodes in Scotland)

Exclusion criteria:

Intervention: -

Control:

A senior GP in each practice reviewed the list to remove patients they considered inappropriate for health or social reasons (such as terminal illness or family or other social crisis).

Baseline characteristics:
<table>
<tr><td></td><td></td><td>I</td><td>C</td></tr>
<tr><td>N</td><td></td><td>288</td><td>612</td></tr>
<tr><td>Age</td><td>Mean (SD)</td><td>49 (15)</td><td>56 (15)</td></tr>
<tr><td>Gender</td><td>Female %</td><td>59.2</td><td>61.1</td></tr>
<tr><td>Ethnicity</td><td>-</td><td>-</td><td>-</td></tr>
<tr><td>Living arrangement</td><td>Lives alone %</td><td>67.5</td><td>45.9</td></tr>
<tr><td>Employment status</td><td>Any employment (%)</td><td>24.1</td><td>48.7</td></tr>
<tr><td>Other</td><td>Deprived %</td><td>79.3</td><td>58.1</td></tr>
</table>

Population: General</td>
<td>Country:

UK (Glasgow, Scotland)</td>
<td>
<table>
<tr><td></td><td>I</td><td>C</td></tr>
<tr><td>Name</td><td>Glasgow Deep End Links Worker Programme (LWP)</td><td>Comparator</td></tr>
<tr><td>Why</td><td>Closer links between general practices and community organizations, and support to access to available community resources, could mitigate the effects of deprivation.</td><td></td></tr>
<tr><td>What</td><td>CLPs made links between practices and community organizations in the local area (eg, walking groups, debt management support, welfare rights, drug and alcohol management support, lunch clubs, befriending schemes, crafting clubs, bereavement support). Each practice devised its own system for GPs and PNs to identify and refer patients who would benefit from help from a CLP who would link them to community-based resources. CLPs met patients, elicited patients' main needs, and worked flexibly, making links with community organizations for patients and, if necessary, supporting patients to attend the organizations' services. Services depended on patients' needs, their enthusiasm to engage, and the availability of local services accessible to patients.</td><td>Comparison practices were not allocated a CLP or a practice development fund.</td></tr>
<tr><td>Who provided</td><td>A practice-attached CLP with a previous working background in community development.

Support included an experienced program director; a community links manager; a learning and evaluation officer, administrative staff and a clinical lead.</td><td></td></tr>
<tr><td>How</td><td>One-to-one, face-to-face, telephone contacts could occur.</td><td></td></tr>
<tr><td>Where</td><td>Usually in the practice, although some home visits could occur, and the CLPs could accompany patients to support their contact with a community organization</td><td></td></tr>
<tr><td>When and How Much</td><td>CLPs and patients could meet as many times they thought necessary</td><td></td></tr>
<tr><td>Tailoring</td><td>Flexible</td><td></td></tr>
</table>

Intervention: Holistic</td>
<td>questionnaire. Subgroup analyses by number of times seen by CLP:

- EQ-5D-5L
- Investigating Choice Experiments for the Preferences of Older People Capability Measure for Adults
- Work and Social Adjustment Scale
- Hospital Anxiety and Depression Scale
- Self-reported exercise
- Number of medications</td>
<td>
Funding source:

NHS Health Scotland (contract 66450/1, 13/08/2014)

Declarations of interest:

None declared</td>
</tr>
<tr>
<td>Morton 2015 (12)</td>
<td>Uncontrolled before-and-after study

Study length:

18 months</td>
<td>Inclusion criteria:

Clients with mild to moderate

mental health difficulties such as anxiety/stress, depression

and low self-esteem.

Exclusion criteria: -

Baseline characteristics:
<table>
<tr><td>N</td><td></td><td>262</td></tr>
<tr><td>Age</td><td>Mean (SD)</td><td>52(11)</td></tr>
<tr><td>Gender</td><td>Female %</td><td>63</td></tr>
<tr><td>Ethnicity</td><td>-</td><td>-</td></tr>
<tr><td>Living arrangement</td><td>-</td><td>-</td></tr>
<tr><td>Employment status</td><td>-</td><td>-</td></tr>
</table></td>
<td>Secondary Care

Country:

UK (Fife, Scotland)</td>
<td>
<table>
<tr><td>Name</td><td>Social prescribing initiative</td></tr>
<tr><td>Why</td><td>Social Prescribing may be of particular benefit to isolated, marginalised and vulnerable social groups including people with mild to moderate mental health problems by addressing some of the consequences of social, economic and health inequalities.</td></tr>
<tr><td>What</td><td>Referral through secondary care or self-referral. A series of free courses were offered to clients with mild to moderate mental health difficulties. Participants could then self-refer to a class of their choosing.</td></tr>
<tr><td>Who provided</td><td>Fife Cultural trust</td></tr>
<tr><td>How</td><td>-</td></tr>
<tr><td>Where</td><td>Courses were delivered by Fife Cultural Trust</td></tr>
<tr><td>When and How Much</td><td>-</td></tr>
<tr><td>Tailoring</td><td>Personalized</td></tr>
</table>

Intervention: Light</td>
<td>Patient outcomes at baseline and after the intervention, subgroups included. Feedback information was posted anonymously:

- Hospital Anxiety and Depression Scale
- General Self-Efficacy Scale
- Warwick Edinburgh Mental Wellbeing Scale</td>
<td>Peer-Review:

Yes

Funding source:

Creative Scotland and Fife Cultural Trust

Declarations of interest:

None declared</td>
</tr>
</table>

<table>
<tr>
<td></td>
<td></td>
<td><table><tr><td>Other</td><td>Attending a psychologist or counsellor %</td><td>34</td></tr></table>
Population: Special (mental health issues)</td>
<td></td>
<td></td>
<td></td>
<td></td>
</tr>
<tr>
<td>Palmer 2017 (81)</td>
<td>Uncontrolled before-and-after study, mixed-methods

Study length:

-</td>
<td>Inclusion criteria:

All participants must have satisfied two or more of the following criteria:

- Aged 18+ years

- Socially isolated (i.e. seeing friends or family less than once a week)

- A frequent user of primary care services or A&E

- Struggling to manage a significant life change

- Struggling to manage their health conditions

- or a carer for somebody else with a long term condition.

Exclusion criteria:

- Active suicidal ideas

- A current or lifetime diagnosis of psychosis, personality disorder or organic mental disorder

Baseline characteristics:
<table>
<tr><td>N</td><td></td><td>245</td></tr>
<tr><td>Age</td><td>Mean Age (Range)</td><td>77 (29-93)</td></tr>
<tr><td>Gender</td><td>Female %</td><td>69</td></tr>
<tr><td>Ethnicity</td><td>-</td><td>-</td></tr>
<tr><td>Living arrangement</td><td>-</td><td>-</td></tr>
<tr><td>Employment status</td><td>-</td><td>-</td></tr>
<tr><td>Other</td><td>Referral reason Struggling with significant life change %</td><td>63</td></tr>
</table>
Population: General</td>
<td>Primary Care

Country:

UK (London Borough of Bexley, South-eastern Greater London)</td>
<td><table>
<tr><td>Name</td><td>Social Prescribing in Bexley</td></tr>
<tr><td>Why</td><td>Develop collaborative working with community groups and move away from a reactive, disease focused, fragmented model of care towards one that is more proactive, holistic and preventative, in which voluntary sector organisations and residents are encouraged to play a greater role in managing care provision</td></tr>
<tr><td>What</td><td>Provides a mix of formal referring and informal signposting according to individual need. Coordinator has access to a range of voluntary and community services and activities who have signed up to be part of the initiative. Additional voluntary and community services which had not formally

signed up to the programme were identified by the coordinator, and formed the basis of informal signposting, where appropriate.</td></tr>
<tr><td>Who provided</td><td>One social prescribing coordinator</td></tr>
<tr><td>How</td><td>Face-to-face</td></tr>
<tr><td>Where</td><td>Coordinator meets clients at a mutually beneficial location, which includes home visits, GP surgeries, and community locations</td></tr>
<tr><td>When and How Much</td><td>-</td></tr>
<tr><td>Modifications</td><td>The pilot included nine GP surgeries, and was initially aimed at clients who were 65 years and over. After the initial three months, the eligibility criteria was widened to include those aged 18 years and over and also included the opportunity for individuals to self-refer. Following a mid-programme review of the pilot, the coordinator identified a barrier to the service; namely that some clients found it difficult to attend the social prescribing assessments at GP surgeries primarily due to mobility. As a result, home visits and assessments in community locations were included at the assessment stage.</td></tr>
<tr><td>Tailoring</td><td>Personalized</td></tr>
</table>
Intervention: Holistic</td>
<td>Patient outcome 6 months before and after referral to social prescribing:

- Warwick Edinburgh Mental Wellbeing Scale

Process of care measures 6 months before and after referral to social prescribing:

- A&E visits
- Length of hospital stay
- London ambulance service calls
- GP visits
- Non-elective admissions</td>
<td>Peer-Review:

No

Funding source:

Bexley Clinical Commissioning Group (CCG) and the London Borough of Bexley

Declarations of interest:

None declared</td>
</tr>
<tr>
<td>Pescheny 2019 (82,83)</td>
<td>Uncontrolled before-and-after study, mixed-methods

Study length:

26 months</td>
<td>Inclusion criteria:

People (18+ years old) with high risk for or diagnosis with type 2 diabetes and chronic obstructive pulmonary disease, people with mild to moderate mental health issues, people who are experiencing loneliness and/or social isolation and carers.

Exclusion criteria: -

Baseline characteristics:
<table><tr><td>N</td><td></td><td>448</td></tr></table></td>
<td>Primary Care

Country:

UK (Luton, Bedfordshire, Eastern England)</td>
<td><table>
<tr><td>Name</td><td>Luton Social prescribing programme</td></tr>
<tr><td>Why</td><td>To reduce health inequalities within Luton.

To promote good health and independence for adults and older people with LTCs and carers.

To empower individuals and communities to have greater control over their own health

To strengthen community development.

To develop and improve the integration of health and social care services.</td></tr>
<tr><td>What</td><td>Referral from a GP to a link worker. Individual assessment to identify the non-medical needs of service users, motivational interviewing and continuous personalised support, and to link service users with non-medical sources of support,</td></tr>
</table></td>
<td>Patient outcomes at Baseline and at follow-up directly post-intervention through link worker. Subgroup analyses for gender, employment status and age groups:

- Short Warwick Edinburgh Mental Wellbeing Scale

At Baseline and at follow-up directly post-intervention:

- International Physical Activity Questionnaire Short Form (IPAQ-SF),</td>
<td>Peer-Review:

Partly (1/2)

Funding source:

Luton Borough Council

Declarations of interest:

None declared</td>
</tr>
</table>

<table>
<tr>
<td></td>
<td></td>
<td>
<table>
<tr><td>Age</td><td>Mean (SD)</td><td>50.3 (16.8)</td></tr>
<tr><td>Gender</td><td>Female %</td><td>65.2</td></tr>
<tr><td>Ethnicity</td><td>-</td><td>-</td></tr>
<tr><td>Living arrangement</td><td>-</td><td>-</td></tr>
<tr><td>Employment status</td><td>Not working %</td><td>59.4</td></tr>
<tr><td>Other</td><td>-</td><td>-</td></tr>
</table>
Population: General
</td>
<td></td>
<td>
<table>
<tr><td></td><td>to help to improve their health and well-being. Re-assessment after a number of sessions and exit interview</td></tr>
<tr><td>Who provided</td><td>Link worker/navigator</td></tr>
<tr><td>How</td><td>-</td></tr>
<tr><td>Where</td><td>-</td></tr>
<tr><td>When and How Much</td><td>Flexible, up to 12 free sessions</td></tr>
<tr><td>Tailoring</td><td>Personalized</td></tr>
</table>
Intervention: Holistic
</td>
<td>MET</td>
<td></td>
</tr>
<tr>
<td>Polley 2019 (84)</td>
<td>Uncontrolled before-and-after study, non-randomized controlled design for process of care measures mixed-methods

Study length:

25 months</td>
<td>
Inclusion criteria:

Initially the inclusion criteria was agreed as systematic referral of people at risk of CVD, i.e. Qrisk2 score of ≥10% and opportunistic referral of people who were socially isolated or lonely. Later on, the inclusion criteria of the opportunistic group was widened.

Exclusion criteria: -

Baseline characteristics:
<table>
<tr><td></td><td></td><td>I</td><td>C</td></tr>
<tr><td>N</td><td></td><td>134</td><td>85</td></tr>
<tr><td>Age</td><td>Mean (SD)</td><td>61.31 (12.6)</td><td>63.3</td></tr>
<tr><td>Gender</td><td>Female %</td><td>53</td><td>47</td></tr>
<tr><td>Ethnicity</td><td>White %</td><td>97.7</td><td>-</td></tr>
<tr><td>Living arrangement</td><td>-</td><td>-</td><td>-</td></tr>
<tr><td>Employment status</td><td>Unemployed %</td><td>17</td><td>-</td></tr>
<tr><td>Other</td><td>Systematic referral %</td><td>67</td><td>-</td></tr>
</table>
Population: Special (CVD risk)
</td>
<td>Primary Care, Primary Care, GP surgeries

Country:

UK (Shropshire, West Midlands)</td>
<td>
<table>
<tr><td></td><td>I</td><td>C</td></tr>
<tr><td>Name</td><td>Shropshire social prescribing service</td><td></td></tr>
<tr><td>Why</td><td>Improve long term health and wellbeing, reduce health inequalities within the community</td><td></td></tr>
<tr><td>What</td><td>Invited by a letter from their GP practice, contacted by the social prescribing advisor. Offered an appointment to review ways of reducing their risk of CVD in the future. Secondly, individuals who met any of the Social Prescribing Service eligibility criteria (loneliness, mental health issues, long term conditions) could also be opportunistically referred via the GP surgeries as well as organisations such as Job centres, and Oswestry library. Referral to a number of organizations.</td><td>Anonymous patient records who had a CVD

Qrisk2 score of 10% or more. They were matched to by age and gender to the social prescribing

group. Control patients may have been invited to use the social prescribing service and not

attended or might not have been included in the initial retrospective list of invitations.</td></tr>
<tr><td>Who provided</td><td>Social prescribing advisor = non-clinical person</td><td></td></tr>
<tr><td>How</td><td>-</td><td></td></tr>
<tr><td>Where</td><td>GP surgery</td><td></td></tr>
<tr><td>When and How Much</td><td>-</td><td></td></tr>
<tr><td>Tailoring</td><td>-</td><td></td></tr>
</table>
Intervention: Medium
</td>
<td>
Patient outcomes at Baseline and 3 months follow-up via questionnaire:

- Measure Yourself Concerns and Wellbeing (MYCaW)
- Patient Activation Measure questionnaire (PAM)
- De Jong Gierveld Loneliness Scale

At Baseline and 3 months follow-up at GP surgeries:

- Physiological data (e.g. blood pressure, cholesterol)

Process of care measures 3 months before and after referral compared to matched control:

- Health services use (GP, Nurse, Unplanned, Inpatient, Outpatient, Total)

At Baseline and 3 months follow-up via questionnaire.

- Hours worked
- Days Ill
- Weeks unemployed

Satisfaction with service at follow-up, anonymously via separate questionnaire.
</td>
<td>Peer-Review:

No

Funding source:

Shropshire Council

Declarations of interest:

Unknown</td>
</tr>
<tr>
<td>Smith 2017 (85)</td>
<td>Uncontrolled before-and-after study, mixed-methods

Study length:

17 months</td>
<td>
Inclusion criteria:

Patients with nonmedical issues, such as social isolation, from GPs who feel the patient could benefit from accessing interventions within the voluntary sector, open to any individual over the age of 18 regardless of their age, gender or medical and social issues

Exclusion criteria: -

Baseline characteristics:
</td>
<td>Primary Care, GP surgeries, Social prescribing clinic

Country:

UK (Loughborough, Leicestershire, East Midlands)</td>
<td>
<table>
<tr><td>Name</td><td>Voluntary Action LeicesterShire's (VAL) social prescribing service</td></tr>
<tr><td>Why</td><td>Social Prescribing aims to:

1. Improve the health and wellbeing of vulnerable adults who are frequent attendees at GP practices

2. Reduce unnecessary repeat appointments thereby saving time and money for GP practices / healthcare systems</td></tr>
</table>
</td>
<td>
Patient outcomes at baseline and unknown follow-up (after referral) via questionnaire:

- Short Warwick and Edinburgh Mental Wellbeing scale

Process of care measures 6
</td>
<td>Peer-Review:

No

Funding source:

West Leicestershire Clinical Commissioning Group (WLCCG)</td>
</tr>
</table>

<table>
<tr>
<td></td>
<td></td>
<td>
<table>
<tr><td>N</td><td></td><td>63</td></tr>
<tr><td>Age</td><td>Mode age group</td><td>45-54</td></tr>
<tr><td>Gender</td><td>-</td><td>-</td></tr>
<tr><td>Ethnicity</td><td>-</td><td>-</td></tr>
<tr><td>Living arrangement</td><td>-</td><td>-</td></tr>
<tr><td>Employment status</td><td>-</td><td>-</td></tr>
<tr><td>Other</td><td>-</td><td>-</td></tr>
</table>
Population: General
</td>
<td></td>
<td>
<table>
<tr><td>What</td><td>Creating a formal way for GPs to refer patients with social, emotional or practical needs to a variety of holistic, local, non-clinical services. Social prescribing clinics receive patients with nonmedical issues, such as social isolation, from GPs who feel the patient could benefit from accessing interventions within the voluntary sector, they are discussing their issues in detail with volunteer social prescribers, then referred to local voluntary sector services which can provide the necessary interventions to resolve the patient's issues</td></tr>
<tr><td>Who provided</td><td>Volunteers from the Patient Participation Group (PPG)</td></tr>
<tr><td>How</td><td>Face-to-face</td></tr>
<tr><td>Where</td><td>Within the GP practice</td></tr>
<tr><td>When and How Much</td><td>30-minute appointment</td></tr>
<tr><td>Tailoring</td><td>Personalized</td></tr>
</table>
Intervention: Medium
</td>
<td>months before and after referral. Subgroup analyses:
- GP appointments
12months before and after referral. Subgroup analyses:
- A&E admissions
- Social Return on Investment</td>
<td>Declarations of interest:
Unknown</td>
</tr>
<tr>
<td>Thomson 2020 (86)</td>
<td>Uncontrolled before-and-after study, mixed methods

Study length:
3 months</td>
<td>
Inclusion criteria: -
Exclusion criteria: -
Baseline characteristics:
<table>
<tr><td>N</td><td></td><td>20</td></tr>
<tr><td>Age</td><td>Mean (Range)</td><td>53 (44-70)</td></tr>
<tr><td>Gender</td><td>Female %</td><td>45</td></tr>
<tr><td>Ethnicity</td><td>White %</td><td>60</td></tr>
<tr><td>Living arrangement</td><td>-</td><td>-</td></tr>
<tr><td>Employment status</td><td>-</td><td>-</td></tr>
<tr><td>Other</td><td>-</td><td>-</td></tr>
</table>
Population: Unclear
</td>
<td>Secondary Care

Country:
UK (Manchester, North West England)</td>
<td>
<table>
<tr><td>Name</td><td>GROW: Art, Park & Wellbeing</td></tr>
<tr><td>Why</td><td>It was hypothesised for the quantitative phase of the study that measures of wellbeing would increase significantly reflecting positive improvements</td></tr>
<tr><td>What</td><td>They were recruited on the basis of accessing local mental health or social services through a community mental health nurse or day centre providing support to vulnerable and disadvantaged adults. Group project of dual engagement in green activity outdoors (including planting and clearing) and creative, arts-based activities indoors responding to collections with broad links to nature themes (including painting, print making and ceramics).</td></tr>
<tr><td>Who provided</td><td>Community partners, mental health nurse, day centre for disadvantaged and vulnerable adults</td></tr>
<tr><td>How</td><td>Face-to-face</td></tr>
<tr><td>Where</td><td>Whitworth Park and used the museum spaces to connect the indoors with the outdoors and nature</td></tr>
<tr><td>When and How Much</td><td>Every Tuesday for 10 weeks for 2-h sessions</td></tr>
<tr><td>Tailoring</td><td>-</td></tr>
</table>
Intervention: Medium
</td>
<td>Patient outcome at Baseline and 10-week endpoint:
- UCL Museum Wellbeing Measure (6 individual mood items)</td>
<td>Peer-Review:
Yes

Funding source:
Not So Grim Up North project funded by Arts Council England (2015–2018; Grant: 29250851). The GROW Project and the Whitworth Gallery's creation of the Art Garden has been funded by Jo Malone, London.

Declarations of interest:
None declared</td>
</tr>
<tr>
<td>Vogelpoel 2014 (87)</td>
<td>Uncontrolled before-and-after study, mixed methods

Study length:
-</td>
<td>
Inclusion criteria:
Older people experiencing social isolation and associated health problems who have single or multi-sensory impairment
Exclusion criteria: -
Baseline characteristics:
<table>
<tr><td>N</td><td></td><td>12</td></tr>
<tr><td>Age</td><td>Mean (Range)</td><td>80 (61-95)</td></tr>
<tr><td>Gender</td><td>Female %</td><td>75</td></tr>
<tr><td>Ethnicity</td><td>-</td><td>-</td></tr>
<tr><td>Living arrangement</td><td>-</td><td>-</td></tr>
</table>
</td>
<td>Primary Care

Country:
UK (Rotherham, South Yorkshire)</td>
<td>
<table>
<tr><td>Name</td><td>Social prescription arts program</td></tr>
<tr><td>Why</td><td>Social isolation is a significant issue for people who are both old and sensory impaired.</td></tr>
<tr><td>What</td><td>Three-stage referral process linking local GPs with the agency.

Participants were invited to the first session, and regular contact was maintained throughout; with reminders for transport arrangements and upcoming developments signposted to participants throughout the process. This aspect became an important part of the engagement and implementation phase, because it enabled participants to implement important choice-making and autonomy building processes. Disability and sensory adapted accessibility was ensured. Sense support staff and communicator guides assisted a visual and tactile arts facilitator, throughout the sessions.</td></tr>
<tr><td>Who provided</td><td>Referral officers, Sense support staff and communicator guides</td></tr>
</table>
</td>
<td>Patient outcome self-reported at Baseline and 12 weeks follow-up:
- Warwick Edinburgh Mental Wellbeing Scale</td>
<td>Peer-Review:
Yes

Funding source:
Voluntary Action Rotherham

Declarations of interest:
Unknown</td>
</tr>
</table>

<table>
<tr>
<td></td>
<td></td>
<td>
<table>
<tr><td>Employment status</td><td>-</td><td>-</td></tr>
<tr><td>Other</td><td>Vision impairment %</td><td>58</td></tr>
</table>
Population: Special (Sensory Impairment)
</td>
<td></td>
<td>
<table>
<tr><td>How</td><td>Face-to-face</td></tr>
<tr><td>Where</td><td>The practical workshop was delivered at a fully accessible resource centre in central Rotherham. Participants were provided travel support.</td></tr>
<tr><td>When and How Much</td><td>12 weeks</td></tr>
<tr><td>Tailoring</td><td>Personalized</td></tr>
</table>
Intervention: Medium
</td>
<td></td>
<td></td>
</tr>
<tr>
<td>Weld 2015 (88)</td>
<td>Uncontrolled before-and-after study, mixed-methods

Study length:

Aug 13-Nov 14 (15 months)</td>
<td>
Inclusion criteria:

- adults (18 years +) living in South Ward who are experiencing low to moderate mental health difficulties (depression, low mood, anxiety) or isolation

Exclusion criteria: -

Baseline characteristics:
<table>
<tr><td>N</td><td></td><td>79</td></tr>
<tr><td>Age</td><td>Mean (Range)</td><td>45 (14.5)</td></tr>
<tr><td>Gender</td><td>Female %</td><td>78.5</td></tr>
<tr><td>Ethnicity</td><td>White british %</td><td>87.3</td></tr>
<tr><td>Living arrangement</td><td>-</td><td>-</td></tr>
<tr><td>Employment status</td><td>Unemployed %

Longterm sick and disabled %</td><td>31.6

21.5</td></tr>
<tr><td>Other</td><td>At least one long term condition or disability %</td><td>63.3</td></tr>
</table>
Population: Special (mental health problems)
</td>
<td>Primary care

Country: UK</td>
<td>
<table>
<tr><td>Name</td><td>Healthier Way to Live Programme (HWTL)</td></tr>
<tr><td>Why</td><td>Improving the mental health and wellbeing, and social support and resilience</td></tr>
<tr><td>What</td><td>Self-referral and referral from partner agencies

- it uses a solution focussed approach to support participants to explore their situation, identify future action to improve their situation and develop an action plan to achieve health and wellbeing goals

- also includes referral and introduction to other elements of the FAHLCs' Wellbeing Project when appropriate, and to other local agencies for specialist support

- 8 local projects deliver a broad base of linked social, non-medical alternatives to positive health promotion that include lunch clubs, community kitchens, weight management groups, community allotments, befriending groups, collective arts and creative activities

- Wellbeing group: weekly group for more isolated people providing mutual contact and support

- Volunteering: support to introduce and support people from the project to existing volunteering opportunities in the centre including; café work, allotment group, 'welcomers'

- Physical activity: supporting and developing existing activity groups; walking, swimming, local gym and developing new activities for people with weight management issues and diabetes

- Café time: working with local people to increase information and experience of healthy eating options in the FAHLC café

- group sessions: develop an action plan with Wellbeing Worker</td></tr>
<tr><td>Who provided</td><td>Wellbeing Worker</td></tr>
<tr><td>How</td><td>Face-to-face</td></tr>
<tr><td>Where</td><td>Healthy Living Centre</td></tr>
<tr><td>When and How Much</td><td>One introductory session and up to six 1:1 sessions after this, wellbeing group weekly, group sessions for 8 weeks</td></tr>
<tr><td>Tailoring</td><td>Personalized</td></tr>
</table>
Intervention: Holistic
</td>
<td>Patient outcome (for all participants at baseline and matched group at baseline, endpoint 8 weeks and follow-up 3 months after first endpoint):

- Shortened seven item version of the Centre for Epidemiological Studies Depression Scale (CES-D)
- Healthy Connections wellbeing questionnaire
- (two questions mental ill health: anxiety)
- overall life satisfaction (questionnaire with a 10 point rating)
- SWEMWBS (Short Warwick Edinburgh Mental Wellbeing Scale)
- mental social wellbeing (5 questions)
- physical activity*
- Exit Questionnaire*
- longer term outcomes*

Economic evaluation:

- - SROI</td>
<td>Peer-Review:

No

Funding source: Big Lottery

Declaration of interest: not mentioned</td>
</tr>
<tr>
<td>Wellbeing Enterprises CIC 2019 (89)</td>
<td>Uncontrolled before-and-after study

Study length:

-</td>
<td>
Inclusion criteria: -

Exclusion criteria: -

Baseline characteristics:
<table>
<tr><td>N</td><td></td><td>3892</td></tr>
</table>
</td>
<td>Primary Care

Country:

UK (Halton, St. Helens and Knowsley,</td>
<td>
<table>
<tr><td>Name</td><td>Ways to Wellbeing</td></tr>
<tr><td>Why</td><td>The program aims to support participants to take control of their own health and to support medical professionals to see patients in the context in which they live their lives and how this affects their health and wellbeing.</td></tr>
<tr><td>What</td><td>Step 1: referral from a variety of sources</td></tr>
</table>
</td>
<td>Patient outcome At Baseline and follow-up:

- Overall Wellbeing
- Depression symptoms
- Loneliness and social isolation
- Medication</td>
<td>Peer-Review:

No

Funding source:

St. Helens Council,</td>
</tr>
</table>

<table>
<tr>
<td></td>
<td></td>
<td><table>
<tr><td>Age</td><td>Age group 50+ %</td><td>66</td></tr>
<tr><td>Gender</td><td>Female %</td><td>75</td></tr>
<tr><td>Ethnicity</td><td>-</td><td>-</td></tr>
<tr><td>Living arrangement</td><td>-</td><td>-</td></tr>
<tr><td>Employment status</td><td>Unemployed %</td><td>39</td></tr>
<tr><td>Other</td><td>Existing disability</td><td>60</td></tr>
</table>
Population: Unclear</td>
<td>Liverpool, North West England)</td>
<td><table>
<tr><td></td><td>Step 2: wellbeing assessment to identify social issues impacting their health and wellbeing, and to identify strengths and assets, develop and implement a Personalised Wellbeing Plan to help people move forward
Step 3: Community Navigation: signpost to additional support
Step 4: Social Prescribing Activity access to over 400 different social prescribing activities
Step 5: Continued Support: Once participants feel confident enough, they are supported into a variety of routeways including, volunteering, employment / self-employment, further training and education or alternative provision</td></tr>
<tr><td>Who provided</td><td>Community Wellbeing Officers (CWO's)</td></tr>
<tr><td>How</td><td>Face-to-face</td></tr>
<tr><td>Where</td><td>-</td></tr>
<tr><td>When and How Much</td><td>-</td></tr>
<tr><td>Tailoring</td><td>Personalized</td></tr>
</table>
Intervention: Medium</td>
<td></td>
<td>Knowsley Council, Halton Borough Council, St. Helens Clinical Commissioning Group, Halton Clinical Commissioning Group, Knowsley Clinical Commissioning Group, Community Fund
Declarations of interest:
Unknown</td>
</tr>
<tr>
<td>Woodall 2018 (90)</td>
<td>Uncontrolled before-and-after study, mixed methods
Study length:
18 months</td>
<td>Inclusion criteria:
14 years and over, registered with a GP surgery
Exclusion criteria: -
Baseline characteristics:
<table>
<tr><td>N</td><td></td><td>436</td></tr>
<tr><td>Age</td><td>Mean (SD)</td><td>53.1 (18.02)</td></tr>
<tr><td>Gender</td><td>Female %</td><td>63.9</td></tr>
<tr><td>Ethnicity</td><td>White British %</td><td>86.9</td></tr>
<tr><td>Living arrangement</td><td>-</td><td>-</td></tr>
<tr><td>Employment status</td><td>-</td><td>-</td></tr>
<tr><td>Other</td><td>-</td><td>-</td></tr>
</table>
Population: General</td>
<td>Primary Care
Country:
UK (Leeds, West Yorkshire, Northern England)</td>
<td><table>
<tr><td>Name</td><td>Wellbeing Coordinators</td></tr>
<tr><td>Why</td><td>To manage patients presenting conditions that can be addressed without medical intervention</td></tr>
<tr><td>What</td><td>Individuals can self-refer into the social prescribing service or GP's, health, social care and other relevant professionals can make a referral. Service users social support needs are explored and Wellbeing coordinators provide them with information, support and guidance in relation to accessing local community activity to improve their health and wellbeing.</td></tr>
<tr><td>Who provided</td><td>Wellbeing Coordinator</td></tr>
<tr><td>How</td><td>Via telephone, face-to-face (if necessary)</td></tr>
<tr><td>Where</td><td>-</td></tr>
<tr><td>When and How Much</td><td>Up to 6 sessions</td></tr>
<tr><td>Tailoring</td><td>Personalized, flexible program</td></tr>
</table>
Intervention: Medium</td>
<td>Patient outcomes at Baseline and 6-16 weeks follow-up via questionnaire. Subgroup analyses by age and gender:
- Warwick Edinburgh Mental Wellbeing Scale
- EQ-5D
- Campaign to End Loneliness Tool
Process of care measures at Baseline and 6-16 weeks follow-up via questionnaire. Subgroup analyses by age:
- GP usage</td>
<td>Peer-Review:
Yes
Funding source:
Leeds South & East Clinical Commissioning Group
Declarations of interest:
None declared</td>
</tr>
<tr>
<td>York CVS 2019 (91)</td>
<td>Uncontrolled before-and-after study, mixed-methods
Study length:
12 months</td>
<td>Inclusion criteria: -
Exclusion criteria: -
Baseline characteristics:
<table>
<tr><td>N</td><td></td><td>71</td></tr>
<tr><td>Age</td><td>Mean (Range)</td><td>54 (24-90)</td></tr>
<tr><td>Gender</td><td>Female %</td><td>69</td></tr>
<tr><td>Ethnicity</td><td>-</td><td>-</td></tr>
<tr><td>Living arrangement</td><td>-</td><td>-</td></tr>
<tr><td>Employment status</td><td>-</td><td>-</td></tr>
</table></td>
<td>Primary Care, GP surgeries
Country:
UK (York, Northern England)</td>
<td><table>
<tr><td>Name</td><td>Ways to Wellbeing, York</td></tr>
<tr><td>Why</td><td>It tackles increasing pressure and stretched resources of GPs by connecting people with voluntary and community organisations</td></tr>
<tr><td>What</td><td>Identify potential networks of support, work with individuals to achieve the outcomes that are important to them, identify and challenge barriers to improving wellbeing. Help individuals to make simple changes to their lifestyle and their home environment, to improve independence and quality of life, help identify their strengths and develop new ways of doing things.
Offer a volunteering pathway to facilitate embedding of new skills and confidence building</td></tr>
<tr><td>Who provided</td><td>Ways to Wellbeing (W2W) coordinator</td></tr>
</table></td>
<td>Patient outcomes at Baseline and 3 months follow-up via questionnaire:
- Short Warwick Edinburgh Mental Wellbeing Scale
- Confidence question (from the original Warwick Edinburgh Mental Wellbeing Scale
- Campaign to End Loneliness Tool, Q2
- Number of people engaging in physical activity as a result of the service</td>
<td>Peer-Review:
No
Funding source:
Unknown
Declarations of interest:
Unknown</td>
</tr>
</table>

<table>
<tr>
<td></td>
<td></td>
<td>
<table>
<tr><td>Other</td><td>Referral reason emotional and mental well being %</td><td>56</td></tr>
</table>

Population: Unclear
</td>
<td></td>
<td>
<table>
<tr><td>How</td><td>One-to-one</td></tr>
<tr><td>Where</td><td>Within GP surgeries</td></tr>
<tr><td>When and How Much</td><td>-</td></tr>
<tr><td>Tailoring</td><td>Personalized, flexible program</td></tr>
</table>

Intervention: Medium
</td>
<td>Process of care measures:

- GP appointments before and 3 months later
- Number of volunteers and hours given
- Social Return on Investment</td>
<td></td>
</tr>
</table>
