# Supplementary figures and images for: Social Prescribing: Systematic Review of the Effectiveness of Psychosocial Community Referral Interventions in Primary Care
Source: Int J Integr Care. 2022 Aug 19;22(3):11. doi: 10.5334/ijic.6472 (PMC9389950; doi:10.5334/ijic.6472)

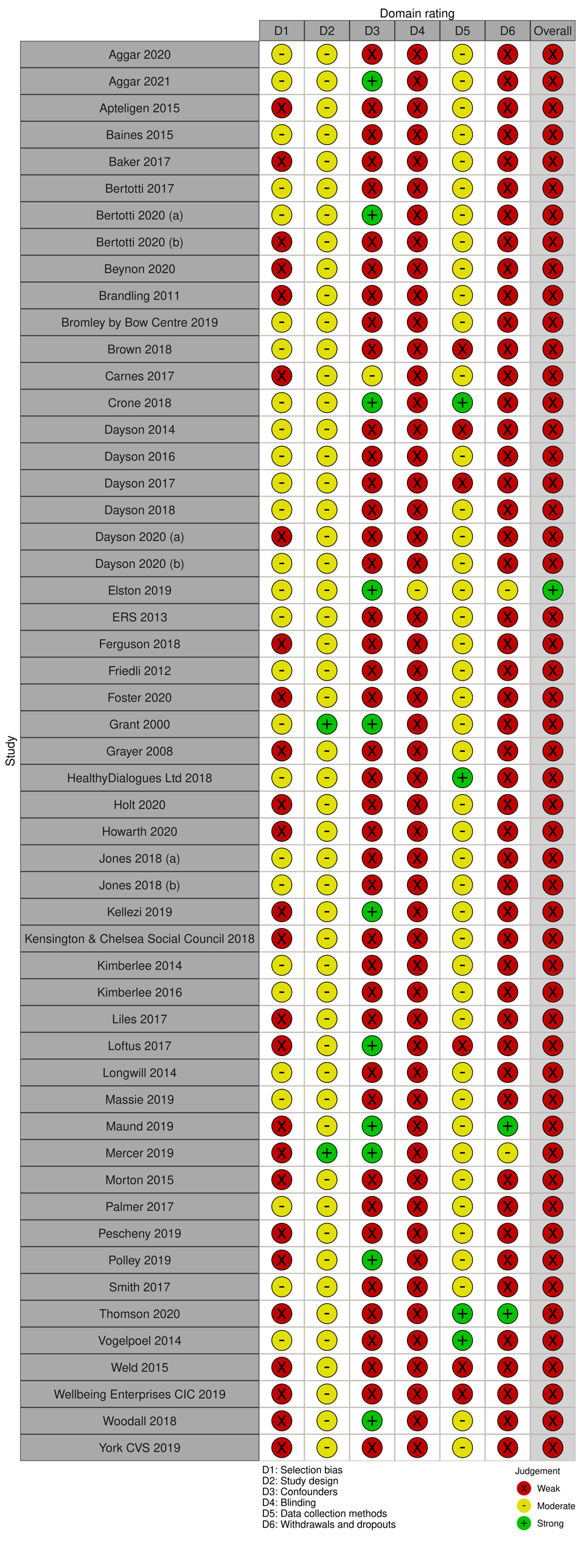

Supplement: Appendix 4. — EPHPP: traffic light plot. [file ijic-22-3-6472-s4.png]
